# Synthesis and In Vitro Cytotoxic Properties of Polycarbo-Substituted 4-(Arylamino)quinazolines

**DOI:** 10.3390/molecules21101366

**Published:** 2016-10-14

**Authors:** Hugues Kamdem Paumo, Tshepiso Jan Makhafola, Malose Jack Mphahlele

**Affiliations:** 1 Department of Chemistry, College of Science, Engineering and Technology, University of South Africa, Private Bag X06, Florida 1710, South Africa; tkamdeh@unisa.ac.za; 2 Department of Life and Consumer Sciences, College of Agriculture and Environmental Sciences, University of South Africa, Private Bag X06, Florida 1710, South Africa; makhat@unisa.ac.za

**Keywords:** 2-aryl-6-bromo-4-chloro-8-iodoquinazolines, amination, cross-coupling, polycarbo-substituted 4-anilinoquinazolines, cytotoxicity, apoptosis

## Abstract

Herein, we describe the synthesis of novel unsymmetrical polycarbo-substituted 4-anilinoquinazolines derived from the 2-aryl-6-bromo-8-iodoquinazolines via one-pot three-step reaction sequences involving initial amination and subsequent double cross-coupling (bis-Suzuki, Sonogashira/Stille or Sonogashira/Suzuki-Miyaura) reactions with different cross coupling partners for the two carbon–carbon bond formation steps. The 4-anilinoquinazolines were evaluated for potential cytotoxicity against three cancer cell lines, namely, human breast adenocarcinoma (MCF-7) cells, human cervical cancer (HeLa) and human lung cancer (A549) cells. The most active compounds, **2b**, **2c**, **3c**, **4a**, **4c** and **5a**, were found to be more selective against the MCF-7 and HeLa cell lines than the human lung carcinoma (A549) cells. We selected compounds **2c**, **3c** and **7a** as representatives for further evaluation for potential to induce apoptosis and/or necrotic properties in the three cancer cell lines. Compound **2c** induced apoptosis of MCF-7 cells through cell membrane alteration. Treatment of Hela and A549 cell lines with compounds **3c** and **7a**, respectively, led to caspase-3 activation in both cell lines. Compound **3c**, on the other hand, caused more necrosis than apoptosis induction in the membrane alteration assay.

## 1. Introduction

**Figure 1:**
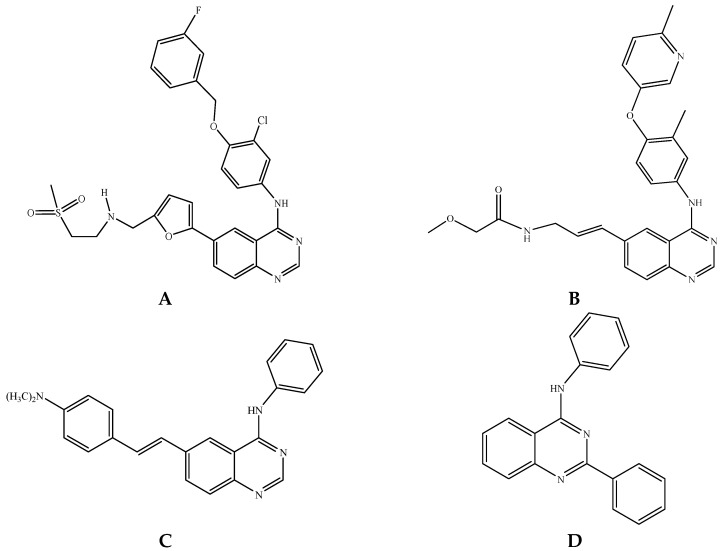
Structures of lapatinib (**A**); CP-724,714 (**B**); (*E*)-6-(4-dimethylaminostyryl)-*N*-phenylquinazolin-4-amine (**C**) and 2-phenyl-4-(phenylamino)quinazoline (**D**).

Quinazolines constitute an important class of compounds with a wide-range of biological properties including anticancer [1,2,3], antibacterial [4], antimicrobial [5] and antihypertensive [6] activities. Among this class of heterocycles, the 4-anilinoquinazolines have established themselves as selective inhibitors of the epidermal growth factor receptor (EGFR) tyrosine kinase phosphorylation, which results from competitive binding at the ATP site [7]. Alkynyl, alkenyl and aryl/heteroaryl substituents are prevalent in biologically relevant 4-anilinoquinazolines such as potent EGFR tyrosine kinase inhibitors [8] and liver X-receptor modulators [9]. These π-conjugated groups when linked to the 4-anilinoquinazoline scaffold have been found to produce potent “turn-on” fluorescent ligands that target the ATP-binding pocket of the EGFR/ERBB family of receptor tyrosine kinases [10]. Lapatinib **A**, a 6-heteroaryl substituted 4-anilinoquinazoline derivative, for example, is an oral dual tyrosine kinase inhibitor (TKI) that targets both EGFR and HER2 to inhibit the proliferation of breast cancer cells (Figure 1) [11]. Optical spectroscopy and DFT calculations recently demonstrated that lapatinib also functions as an environmentally responsive “turn-on” fluorophore [12]. The 6-vinyl–substituted 4-anilinoquinazoline derivative **B** (CP-724,714), on the other hand, is a selective ERBB2 angiogenesis inhibitor under investigation for the treatment of breast, ovarian and other types of cancer (Figure 1) [13]. The analogous (*E*)-6-(4-dimethylaminostyryl)-*N-*phenylquinazolin-4-amine **C** was found to exhibit ERBB2-induced fluorescence thus demonstrating its utility as a turn-on fluorescence kinase inhibitor in human cervical cancer (MCF-7) cells [14]. 2-Phenyl-4-(phenylamino)quinazoline **D**, on the other hand, has emerged as a potent photosensitizer with anti-proliferative activity and DNA damage in L1210 cells [15]. This compound was also found to induce programmed cell death in leukemia cells through mitochondrial/caspase 9/caspase 3-dependent pathway upon UVA irradiation [15].

Based on the structure of the ATP-binding pocket of protein kinases, 4-anilinoquinazolines as ATP-mimics are well established as an effective template for the design of inhibitors of EGFR and other members of the ERBB kinase family [16,17]. Given the importance of carbon-substituted primary 4-anilino substituted quinazolines in pharmaceutical compounds or materials [18], we decided to develop efficient methods for the synthesis of novel unsymmetrical polycarbo-substituted 4-anilinoquinazolines based on the 2-aryl-6-bromo-4-chloro-8-iodoquinazolines. Herein we describe single-pot three-step sequential reactions involving initial amination of the 2-aryl-6-bromo-4-chloro-8-iodoquinazolines followed by two-step (bis-Suzuki-Miyaura, Sonogashira/Stille or Sonogashira/Suzuki-Miyaura) cross-coupling reaction to afford the requisite unsymmetrical 2,6,8-trisubstituted 4-(arylamino)quinazolines. The prepared compounds were screened for potential in vitro cytotoxicity against the human breast adenocarcinoma (MCF-7), human cervical cancer (HeLa) and human lung cancer (A549) cells. The most active compounds were further evaluated for potential to induce apoptosis to identify their mode of anti-proliferative activity.

## 2. Results and Discussion

### 2.1. Amination of ***1a**–**d*** with 3-Fluoroaniline

**Scheme 1:**
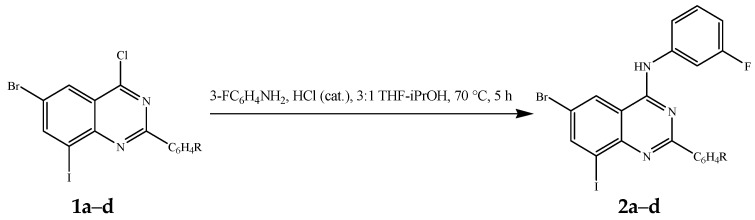
Dechloro-amination of **1a**–**d** with 3-fluoroaniline.

The presence of a halogen atom (F, Cl, and Br) on the 3-position of the aniline ring was previously found to enhance the potency of the 4-anilinoquinazolines as non-competitive antagonists of metabotropic glutamate receptor 5 (mGlu_5_) compared to unsubstituted aniline derivatives [19]. Based on this literature precedent, we decided to incorporate a 3-fluoroaniline moiety on the 4-position of the 2-aryl-6-bromo-4-chloro-8-iodoquinazoline framework to serve as a template for the design of unsymmetrical polycarbo-substituted 4-anilinoquinazolines. Although the C-4 position of 4-chloroquinazoline moiety is known to be the most electronegative due to α-nitrogen effect [17], attempts to an effective amination of the known 2-aryl-6-bromo-4-chloro-8-iodoquinazolines **1a**–**d** [20] with 3-fluoroaniline in various solvents (e.g., DMSO, DMF, and THF-DCM) at RT or under reflux led to the recovery of the starting material. However, the traces of the desired product, 4-anilinoquinazoline, were detected in tetrahydrofuran-isopropyl alcohol (THF-iPrOH) mixture under reflux. Since the quinazoline ring undergoes protonation at *N*-1 in dilute acidic solution [21,22], we envisaged that protonation at this position would render the heterocyclic ring more electron deficient and therefore C-4 more electrophilic. Based on this assumption, we subjected compounds **1a**–**d** to 3-fluoroaniline in THF-iPrOH mixture in the presence of hydrochloric acid as a catalyst at 65 °C for 5 h (Scheme 1). To our delight, we isolated the corresponding 4-(3-fluorophenylamino)quinazolines **2a**–**d** in high yield and purity (Table 1) without the need for column chromatography. These compounds are easily distinguished from the corresponding substrates by the presence of additional signals in the aromatic region of their proton and carbon-13 nuclear magnetic resonance (NMR) spectra (^1^H- and ^13^C-NMR) and the NH signal at δ_H_ ca. 10.10 ppm. The presence of the latter is further confirmed by a band in the region ν_max_ 3413–3452 cm^−1^ of their IR spectra. Moreover, the molecular ion region of their mass spectra revealed the absence of the M+ and M + 2 peaks in the ratio 3:1 due to the ^35^Cl and ^37^Cl isotope observed in the spectra of the corresponding substrates.

We decided to take advantage of the trend in reactivity of the C-sp^2^-X bonds in transition metal catalyzed cross-coupling (trend: C-sp^2^-I > C(4)-Cl > C-sp^2^-Br > C(2)-Cl > C-sp^2^-Cl) [18] and subjected compounds **1a**–**d** to one-pot successive two- and three-step reaction sequences involving initial amination and subsequent Pd catalyzed cross-coupling reactions to afford novel unsymmetrical polycarbo-substituted 4-anilinoquinazolines.

### 2.2. One-Pot Two-Step Sequential Amination and Cross-Coupling of Substrates ***1a**–**d***

#### 2.2.1. One-Pot Sequential Amination and Suzuki-Miyaura Cross-Coupling of **1a**–**d**

**Scheme 2:**
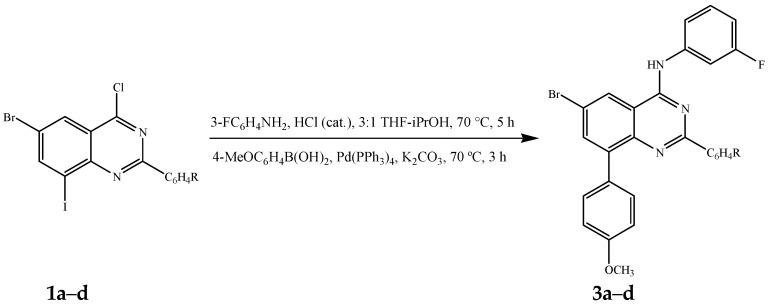
One-pot chemoselective amination and Suzuki-Miyaura cross-coupling of **1a**–**d**.

**Figure 2:**
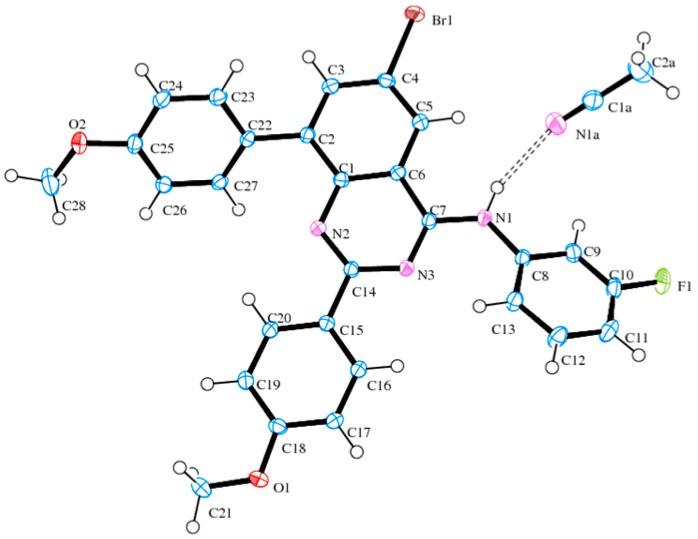
Oak Ridge Thermal Ellipsoid Plot (ORTERP) diagram (50% probability level) of compound **3d**. Hydrogen bond is shown as dashed line.

Initial amination of **1a**–**d** with 3-fluoroaniline under the same reaction conditions described in Scheme 1 followed by the Suzuki-Miyaura cross-coupling with 4-methoxyphenylboronic acid (1.2 equivalent) in the presence of commercially available tetrakis(triphenylphosphine)palladium(0) (Pd(PPh_3_)_4_, purchased from Sigma-Aldrich, Schnelldorf, Germany) as a source of active Pd(0) afforded the 8-aryl substituted derivative **3a**–**d**, exclusively (Scheme 2, Table 2). Hitherto, the 4-alkynyl-2-aryl-6,8-dibromoquinazolines [23] and the 6,8-dibromo-2,4-diaminoquinazoline [24,25] have been found to undergo Suzuki-Miyaura cross-coupling at the 6- and 8-positions without selectivity due to comparable Csp^2^-Br bond dissociation energies. Crystals of quality suitable for X-ray diffraction were obtained for **3d** by slow evaporation of acetonitrile and the molecular structure of compounds **3** was further confirmed by single crystal X-ray crystallography. In the crystal lattice, compound **3d** adopts the anticipated anti-orientation (anti-5) of the 4-(3-fluorophenyl)amino group with respect to the quinazoline 5-position (Figure 2) [26]. The molecule is hydrogen bonded to the solvate, acetonitrile, through σ bonding [27] involving N-H and the lone-pair electrons of nitrogen atom of the solvent with N-H^…^N≡CCH_3_ bond angle and N-H^…^N bond distance of 159.3° and 2.23 Å, respectively. The 8-aryl ring and the 2-aryl ring are distorted from the plane of the quinazoline framework with average torsion angles of ca. 138.65° and ca. 173.22°, respectively.

#### 2.2.2. One-Pot Sequential Amination and Sonogashira Cross-Coupling of **1a**–**d**

**Scheme 3:**
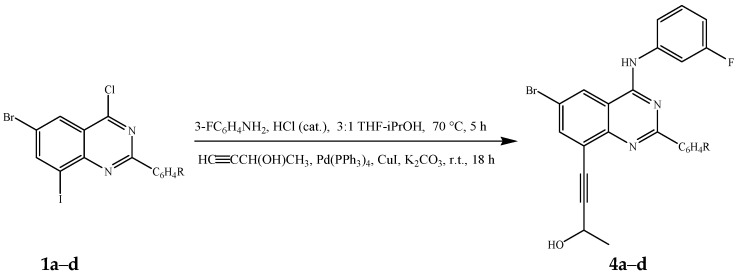
One-pot chemoselective amination and Sonogashira cross-coupling of **1a**–**d**.

Amination of compounds **1a**–**d** with 3-fluorophenylaniline was conducted as described above. In each case, the reaction mixture was cooled to r.t. followed by addition of 3-butyn-2-ol (1.2 equivalent), PdCl_2_(PPh_3_), CuI and K_2_CO_3_ under argon atmosphere (Scheme 3). The reaction mixtures were stirred at r.t. for 18 h followed by aqueous work-up and column chromatography on silica gel. In each case, we isolated in sequence traces of the homocoupled dimer and a single cross-coupled product characterized using a combination of NMR and IR spectroscopic techniques as the 8-alkynylated derivatives **4a**–**d** (Scheme 3, Table 3). Their high resolution mass spectral data revealed the presence of the ^79^Br and ^81^Br containing molecular ions in the ratio 1:1.

The success of the above one-pot two-step sequential amination and cross-coupling reactions encouraged us to evaluate the reactivity of **1a**–**d** in single-pot three-step reaction sequences involving initial amination and subsequent double cross-coupling (bis-Suzuki, Sonogashira/Stille, and Sonogashira/Suzuki-Miyaura) with different coupling partners. In each case, we opted for the use of the same catalyst complex or mixture for the two cross-coupling steps and only varied the reaction conditions as described below.

### 2.3. One-Pot Sequential Three-Step Amination and Double Cross-Coupling of ***1a**–**d***

#### 2.3.1. One-Pot Sequential Amination and Bis-Suzuki Cross-Coupling of **1a**–**d**

**Scheme 4:**
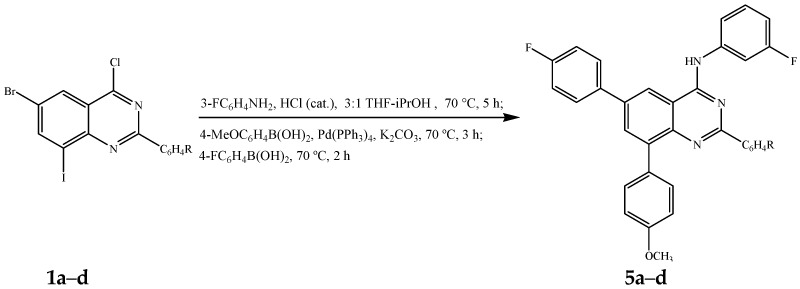
One-pot sequential amination and bis-Suzuki cross-coupling of **1a**–**d**.

The first two steps involving amination of **1a**–**d** with 3-fluoroaniline and Suzuki-Miyaura cross-coupling with 4-methoxyphenylboronic acid (1.2 equivalent) were conducted as described in Scheme 2. After 2 h (thin layer chromatography monitoring), each reaction mixture was treated with 4-fluorophenylboronic acid (1.3 equivalent) and then heated at 70 °C for additional 2 h to afford upon column chromatography on silica gel the unsymmetrical polyaryl-substituted 4-anilinoquinazolines **5a**–**d** exclusively (Scheme 4, Table 4).

#### 2.3.2. One-Pot Sequential Amination, Sonogashira and Stille Cross-Coupling of **1a**–**d**

**Scheme 5:**
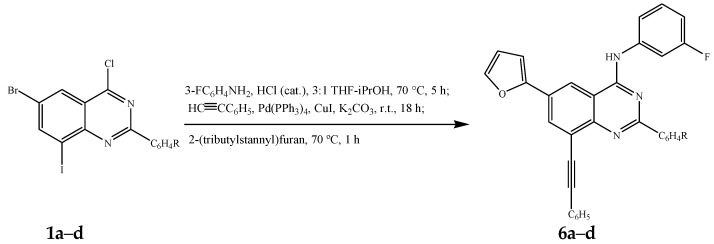
One-pot successive amination, Sonogashira and Stille cross-coupling of **1a**–**d**.

The Stille cross-coupling step (step iii) was achieved by addition of 2-(tributylstannyl)furan (1.2 equivalent) in THF-iPrOH to the amination-Sonogashira cross-coupling mixture (steps i and ii) prepared as described in Scheme 3 followed by heating at 70 °C for 1 h to afford the corresponding unsymmetrical polycarbo-substituted quinazolines **6a**–**d** in a single-pot operation albeit in moderate yields (Table 5, Scheme 5).

#### 2.3.3. One-Pot Sequential Amination, Sonogashira and Suzuki-Miyaura Cross-Coupling of **1b**–**d**

**Scheme 6:**
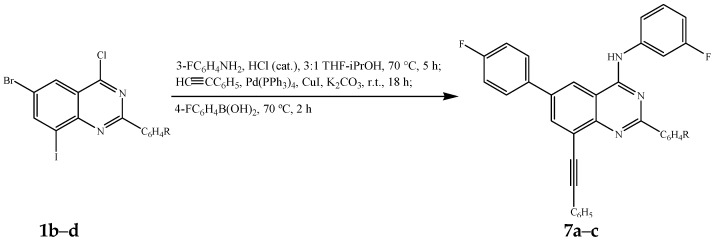
One-pot successive amination, Sonogashira and Suzuki cross-coupling of **1b**–**d**.

Amination of **1b**–**d** with 3-fluoroaniline under reflux was followed by the Sonogashira cross-coupling step with phenylacetylene (1.2 equivalent) at RT under the same reaction conditions outlined in Scheme 3. The third step was achieved by addition of an aqueous solution of 4-fluorophenylboronic acid (1.3 equivalent) followed by heating at 70 °C for 2 h to afford compounds **7a**–**c** (Scheme 6, Table 6). One-pot reaction involving sequential Sonogashira and Suzuki-Miyaura cross-coupling reactions are very rare in the literature [28].

As a guide to polycarbo-substituted 4-anilinoquinazoline derivatives with potential antitumor activity, we decided to evaluate the compounds prepared in this investigation for potential in vitro anti-proliferative properties to correlate between both structural variations and cytotoxic activity.

### 2.4. Biological Evaluation

#### 2.4.1. In Vitro Cytotoxicity Evaluation of Compounds **2a**–**d**, **3a**–**d**, **4a**–**d**, **5a**–**d**, **6a**–**d** and **7a**–**c**

These compounds were evaluated for their in vitro cytotoxicity against the breast cancer (MCF-7), human cervical cancer (HeLa) and human lung carcinoma (A549) cell lines using the 3-(4,5-dimethylthiazole-2-yl)-2,5-diphenyltetrazoliumbromide based colorimetric cell viability (MTT) assay [29]. The compounds were assayed at concentrations ranging from 0.1 to 100 μg/mL with DMSO and Doxorubicin hydrochloride as the negative and positive control, respectively (0.01% DMSO was used as the negative control). DMSO at 1% or less has been reported to have no apparent effect on proliferation of HeLa and Caco2 cells for up to 48 h [30,31]. The LC_50_ (lethal concentration at which 50% of the cells are killed) values of compounds **2**–**4** and **5**–**7** (average from three independent experiments) against Doxorubicin hydrochloride as a reference drug are represented in Table 1 and Table 2, respectively (see Supplementary Materials for the corresponding cell viability percentages and graphs for each compound). The results are presented in μM concentrations, taking into account the molecular weights of the compounds. Within series **2**, compounds **2b** and **c** were found to exhibit selectivity and significant in vitro cytotoxicity against the breast cancer (MCF-7) cells with LC_50_ values of 3.12 and 2.96 μM, respectively. However, these compounds exhibit moderate cytotoxicity against the other two cell lines. On the other hand, a phenyl group at the 2-position of **2a** resulted in moderate activity against MCF-7 cells. A bulky methoxy group at the para-position of the 2-aryl substituent of **2d** resulted in complete loss of activity against the three cell lines. However, replacement of iodine with a 4-methoxyphenyl group in compound **3c** substituted with a 4-chlorophenyl group at position 2 resulted in the highest activity against the HeLa cell lines (LC_50_ < 0.10 μM) and significant cytotoxicity against the MCF-7 and A549 cells with LC_50_ values of 6.55 μM and LC_50_ 10.88 μM, respectively. Compounds **4a** and **4c** substituted with a 3-hydroxybutyn-1-yl group at the 8-position exhibit selectivity and significant cytotoxicity against the HeLa cell line with LC_50_ values of 6.29 μM and 1.73 μM, respectively. For the three series of compounds shown in Table 7, a combination of the 2-(4-chlorophenyl) group and 8-bromo substituent resulted in highest cytotoxicity against MCF-7 and HeLa cells as observed for **2c**, **3c** and **4c**.

We observed that replacement of bromine atom with a carbon-based substituent in the series **5a**–**d**, **6a**–**d** and **7a**–**c** diminished activity against all the three cell lines except for compounds **5a** and **7a** bearing the 6-(4-fluorophenyl) ring and a small phenyl or 4-fluorophenyl ring at the 2-position, respectively (Table 8). Compound **5a** exhibits selectivity and higher activity than the reference standard against the HeLa cell line with LC_50_ value of 1.45 μM. The presence of the 4-fluorophenyl group at the 2- and 6-position in compound **7a** also resulted in significant cytotoxicity against the A549 cells (LC_50_ = 6.04 μM) relative to Doxorubicin, but moderate activity against the MCF-7 (LC_50_ = 9.48 μM) and HeLa (LC_50_ = 15.49 μM) cell lines. The observed cytotoxicity of compounds **5a** and **7a** is presumably the consequence of the presence of the 6-(4-fluorophenyl) ring due to its enhanced lipophilicity [32], resulting from the non-polarizability of the Csp^2^-F bond, which involves compatible 2s and 2p orbital overlap [33]. Moreover, fluorine atom is also known to exhibit strong polar interaction with the protein cavity [34].

From these preliminary in vitro cytotoxicity results and SAR, it is observed that the presence of a relatively bulky 4-methoxyphenyl group at the 2-position of the 4-(arylamino)quinazolines generally leads to diminished cytotoxicity. However, significant cytotoxicity was observed for compounds **3c** and **5a** bearing the 4-methoxyphenyl substituent at the 8-position. A bulky aryl group such as 4-chlorophenyl- or 4-methoxyphenyl substituent at the 2-position of the polycarbo-substituted quinazolines **5**–**7** resulted in loss of activity compared to derivatives bearing relatively smaller phenyl or 4-fluorophenyl group at this position. Since cytotoxicity does not define a specific cellular death mechanism and considering the literature precedent on the pro-apoptotic properties of the 2-substituted 4-anilinoquinazoline derivatives [35], we decided to evaluate how compounds **2b**, **2c**, **3c**, **4a**, **4c**, **5a** and **7a** induce cell death in cancer cells. We selected compounds **2c**, **3c** and **7a** as representatives for further evaluation for potential to induce apoptosis and/or necrotic properties in the three cancer cell lines.

#### 2.4.2. Evaluation of Cell Death Pathways

##### Flow Cytometric Analysis

**Figure 3:**
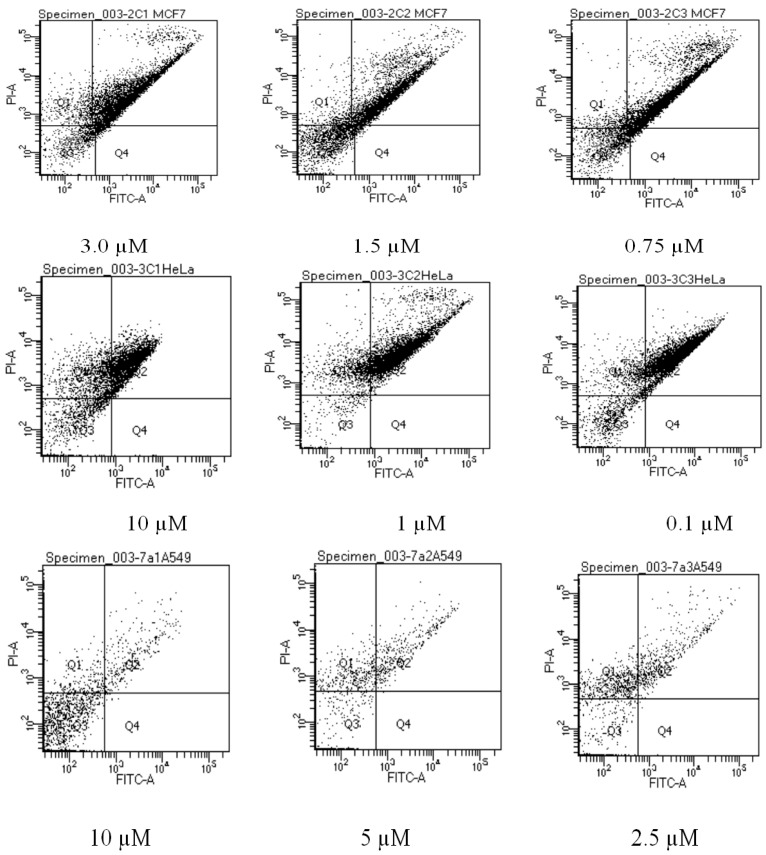
Effects of compound **2c**, **3c**, and **7a** on the induction of apoptosis in MCF-7, HeLa and A549 cells stained with Annexin V-FITC and propidium iodide (PI), respectively.

The population of apoptotic cells was determined using flow cytometry, which is one of the tools for the investigation of molecular and morphological events occurring during cell death and cell proliferation. For this purpose, we used the Annexin-V-FLOUS staining kit and the results from flow cytometry are summarized in Figure 3 and Table 9. Annexin-V is a member of the Annexin family of intracellular proteins that binds to phosphatidylserine (PS). Propidium iodide (PI) is widely used in conjunction with Annexin-V to determine if cells are viable, apoptotic, or necrotic through differences in plasma membrane integrity and permeability. PS is normally confined to the inner plasma membrane in healthy cells but is translocated to the outer membrane leaflet in response to stimuli. This membrane flip flop is caspase depended [36]. PI, on the other hand, does not stain live or early apoptotic cells due to the presence of an intact plasma membrane [37]. In these assays, healthy cells are doubly negative to Annexin-V and PI, whereas cells in the early phases of apoptosis are Annexin-V-positive but PI-negative, and secondary necrotic cells are doubly positive to Annexin-V and PI. In Figure 3, Q1 contains necrotic cells (cells stained predominantly with PI), Q2 has late apoptotic cells (cells stained by both PI and Annexin-V), Q3 has healthy cells (not stained by either PI or Annexin-V) and Q4 has early apoptotic cells (stained predominantly by Annexin-V). As shown in Figure 3 and Table 3, the population of apoptotic cells in MCF-7 cells treated with compound **2c** increased with an increase in concentration. The percentage cell population in Q2 were 30.4%, 47.7% and 67.1% at 0.75 µM, 1.5 µM and 3.00 µM, respectively. This increase in apoptotic cells is comparable to the positive control, doxorubicin hydrochloride. At all tested concentrations, there was up to 30% of cells that stained doubly negative for both Annexin-V and PI (Q3), an indication of unaffected cells.

At the highest concentration tested, compound **3c** had a higher percentage of Hela cells that stained positive for PI, followed by cells that stained positive for both PI and Annexin-V. More cells are necrotic at the highest concentration tested. Even though a considerable percentage of the cell population is undergoing apoptosis, especially at lower concentrations (Q2), more cells seem to be necrotic. This is further proved by fewer cells in Q3 at all tested concentrations a clear sign a necrotic response. The lung cancer A549 cells did not respond to Accuatase^®^ enzyme detachment medium and were therefore scraped from the surface of the tissue culture plates. This process caused cell membrane damage as evident by 16.45% of healthy cells (Q3) in the negative control (Table 9). Lung cancer A549 cells treated with compound **7a** gave inconclusive results. Based on the flow cytometry results in Table 9, the percentage of unaffected cells increased with an increase in concentration, which is contrary to the MTT assay findings. Furthermore, necrotic cells decreased at higher concentrations tested. The observed discrepancies between the MTT and flow cytometry results for this compound is presumably due to cell membrane damage that occurred during detachment of cells from the culture plates which led to nonspecific staining to produce mixed fluorescence signals in this assay.

In the last part of this investigation, we evaluated compounds **2c**, **3c** and **7a** for caspase-3 activation in MCF-7, HeLa and A549 cells.

##### Caspase-3 Activation Assay

**Figure 4:**
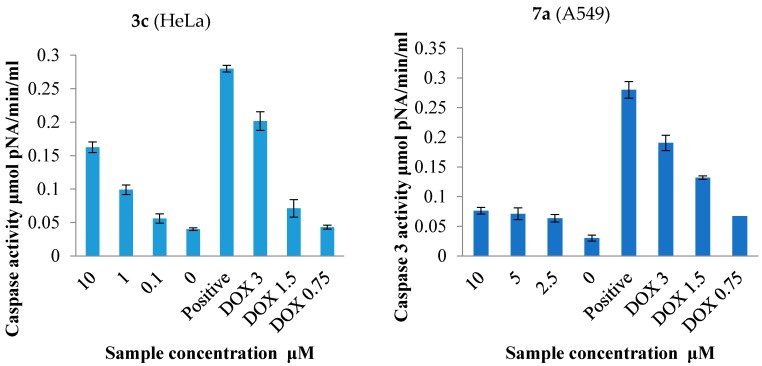
Effects of compound **3c** and **7a** on caspase-3 activity in HeLa and A549 cell lines compared to the negative control (untreated cells) and doxorubicin hydrochloride.

Caspase-3 is a cysteine protease involved in programmed cell death (apoptosis) and monitoring caspase-3 enzyme activity is a fundamental means to measure apoptosis in cells [38,39]. Caspase-3 is required for DNA fragmentation and morphological changes associated with apoptosis [40]. Although the MCF-7 cells undergo morphological apoptosis after treatment with a variety of agents [41], we observed no change in caspase-3 activity in MCF-7 cells treated with compound **2c** (results not included). It has been reported in the literature that the breast cancer cell line MCF-7 lacks a functional caspase-3 gene product [40] and thus apoptosis induction in these cells may be through other molecular pathways that do not involve caspase activation. The results from caspase-3 activation (Figure 4) reveal that compound **3c** and more so **7a** do not induce significant apoptosis compared to the reference standard, doxorubicin hydrochloride.

## 3. Experiments

### 3.1. General

Melting points were recorded on a Thermocouple digital melting point apparatus (Stuart, Staffordshire, UK) and are uncorrected. IR spectra were recorded as powders using a Bruker VERTEX 70 FT-IR Spectrometer (Bruker Optics, Billerica, MA, USA) with a diamond ATR (attenuated total reflectance) accessory by using the thin-film method. For column chromatography, Merck kieselgel 60 (0.063–0.200 mm) (Merck KGaA, Frankfurt, Germany) was used as stationary phase. NMR spectra were obtained as CDCl_3_ solutions using Agilent 500 MHz NMR (Agilent Technologies, Oxford, UK) spectrometer and the chemical shifts are quoted relative to the TMS peak. Low- and high-resolution mass spectra were recorded using Waters Synapt G2 Quadrupole Time-of-flight mass spectrometer (Waters Corp., Milford, MA, USA) at the University of Stellenbosch Mass Spectrometry Unit. The synthesis and characterization of compounds **1a**–**d** have been described before [20].

### 3.2. Typical Procedure for the Dechloro-Aminination of ***1a**–**d***

A stirred mixture of **1** (1 equivalent), 3-fluoroaniline (1.1 equivalent) and concentrated HCl (0.01 g, 0.27 mmol) in 3:1 THF-isopropanol (5 mL/mmol of **1**) was heated at 70 °C for 5 h and then allowed to cool to RT The reaction mixture was quenched with an ice-cold water and the product was extracted into ethyl acetate. The organic layers were washed with an aqueous solution of NaHCO_3_, dried over anhydrous MgSO_4_, filtered and evaporated under reduced pressure to afford **2** as a solid. The following products (**2a**–**d**) were prepared in this fashion:

*6-Bromo-N-(3-fluorophenyl)-8-iodo-2-phenylquinazolin-4-amine* (**2a**). Yellow solid (1.07 g, 92%), mp. 199–200 °C; ν_max_ (ATR) 445, 551, 701, 770, 957, 1128, 1306, 1397, 1448, 1541, 1590, 1616, 3424 cm^−1^; δ_H_ (500 MHz, DMSO-*d*_6_) 7.02 (td, *J* = 2.5 and 8.0 Hz, 1H), 7.50 (q, *J* = 8.0 Hz, 1H), 7.55–7.58 (m, 3H), 7.75 (d, *J* = 8.5 Hz, 1H), 7.95 (dt, *J* = 2.0 and 11.5 Hz, 1H), 8.48–8.49 (m, 2H), 8.57 (d, *J* = 1.5 Hz, 1H), 8.89 (d, *J* = 1.5 Hz, 1H), 10.13 (s, 1H); δ_C_ (125 MHz, DMSO-*d*_6_) 105.2, 109.3 (d, ^2^*J*_CF_ = 26.5 Hz), 110.8 (d, ^2^*J*_CF_ = 20.9 Hz), 115.7, 118.2 (d, ^4^*J*_CF_ = 2.8 Hz), 119.4, 126.3, 128.5, 129.1, 130.6 (d, ^3^*J*_CF_ = 8.5 Hz), 131.5, 137.9, 141.2 (d, ^3^*J*_CF_ = 11.3 Hz), 145.2, 149.2, 157.9, 160.5, 162.4 (d, ^1^*J*_CF_ = 239.8 Hz); *m*/*z* 520 (100, MH^+^); HRMS (ES): MH^+^, found 519.9230. C_20_H_13_BrFIN_3_^+^ requires 519.9243.

### 3.3. Typical Procedure for the One-Pot Two-Step Sequential Amination and Suzuki–Miyaura Cross-Coupling of ***1a**–**d***

*6-Bromo-N-(3-fluorophenyl)-8-(4-methoxyphenyl)-2-phenylquinazolin-4-amine* (**3a**). A stirred mixture of **1a** (0.50 g, 1.12 mmol), 3-fluoroaniline (0.14 g, 1.23 mmol) and concentrated HCl (0.01 g, 0.27 mmol) in 3:1 THF-isopropanol (*v*/*v*, 10 mL) was heated at 70 °C for 5 h until the starting material was completely consumed (thin layer chromatography monitoring) and then cooled to RT. The cooled mixture was treated sequentially with Pd(PPh_3_)_4_ (0.064 g, 0.056 mmol) and a solution of K_2_CO_3_ (0.46 g, 3.36 mmol) in water (5 mL) followed by purging with argon gas for 30 min. A solution of 4-methoxyphenylboronic acid (0.20 g, 1.34 mmol) in THF (5 mL) was then injected using a syringe. The reaction mixture was stirred at 70 °C for additional 3 h and then quenched with an ice-cold water. The product was extracted with ethyl acetate and the combined organic layers were washed with water, dried over Mg_2_SO_4_, filtered and evaporated under reduced pressure. The residue was purified by column chromatography on silica gel to afford **3a** as a yellow solid (0.45 g, 82%), *R*_f_ (toluene) 0.40, mp. 210–211 °C; ν_max_ (ATR) 450, 501, 565, 676, 717, 773, 829, 975, 1061, 1147, 1289, 1351, 1395, 1440, 1557, 1607, 2846, 3018, 3426 cm^−1^; δ_H_ (500 MHz, DMSO-*d*_6_) 3.86 (s, 3H), 7.00 (td, *J* = 2.0 and 8.5 Hz, 1H), 7.10 (d, *J* = 8.5 Hz, 2H), 7.50 (m, 4H), 7.77 (d, *J* = 8.5 Hz, 2H), 7.79 (d, *J* = 8.5 Hz, 1H), 7.97 (d, *J* = 2.5 Hz, 1H), 8.00 (dt, *J* = 2.0 and 8.5 Hz, 1H), 8.30–8.32 (m, 2H), 8.82 (d, *J* = 2.0 Hz, 1H), 10.04 (s, 1H); δ_C_ (125 MHz, DMSO-*d*_6_) 55.6, 109.1 (d, ^2^*J*_CF_ = 26.6 Hz), 110.3 (d, ^2^*J*_CF_ = 20.8 Hz), 113.7, 116.2, 118.0 (d, ^4^*J*_CF_ = 2.7 Hz), 119.0, 124.5, 128.3, 129.0, 129.3, 129.7, 130.5 (d, ^3^*J*_CF_ = 9.5 Hz), 131.0, 132.5, 135.9, 138.5, 141.3 (d, ^3^*J*_CF_ = 10.3 Hz), 147.3, 157.7, 158.9, 159.5, 162.5 (d, ^1^*J*_CF_ = 239.0 Hz); *m*/*z* 500 (100, MH^+^); HRMS (ES): MH^+^, found 500.0780. C_27_H_20_BrFN_3_O^+^ requires 500.0774.

### 3.4. Typical Procedure for the One-Pot Two-Step Sequential Amination and Sonogashira Cross-Coupling of ***1a**–**d***

*4-[6-Bromo-4-(3-fluorophenylamino)-2-phenylquinazolin-8-yl]but-3-yn-2-*ol (**4a**). A stirred mixture of 1a (0.40 g, 0.89 mmol), 3-fluoroaniline (0.11 g, 0.98 mmol) and concentrated HCl (0.01 g, 0.27 mmol) in 3:1 THF-isopropanol (*v*/*v*, 10 mL) in a two necked flask equipped with rubber septum and a condenser fitted with calcium chloride tube was heated at 70 °C for 5 h. After cooling, Pd(PPh_3_)_4_ (0.051 g, 0.044 mmol), CuI (0.008 g, 0.044 mmol) K_2_CO_3_ (0.37 g, 2.67 mmol) and 3-butyn-2-ol (0.081 g, 1.15 mmol) in THF (5 mL) were introduced to the mixture under argon atmosphere. A balloon filled with argon was fitted to the top of the condenser and the mixture was stirred for additional 18 h at RT under argon atmosphere. The mixture was quenched with an ice-cold water and the product was extracted with ethyl acetate. The combined organic layers were washed with water, dried over anhyd. MgSO_4_, filtered and evaporated under reduced pressure. The residue was purified by column chromatography on silica gel to afford 4a as an orange solid (0.26 g, 63%), *R*_f_ (9:1 toluene–ethyl acetate) 0.26, mp. 172–174 °C; ν_max_ (ATR) 539, 697, 712, 782, 861, 1071, 1142, 1368, 1470, 1524, 1556, 1612, 2924, 3070, 3308, 3478 cm^−1^; δ_H_ (500 MHz, DMSO-*d*_6_) 1.51 (d, *J* = 6.5 Hz, 3H), 4.76 (d, *J* = 6.0 Hz, 1H), 5.60 (d, *J* = 6.0 Hz, 1H), 7.00 (td, *J* = 2.5 and 8.0 Hz, 1H), 7.51 (q, *J* = 8.0 Hz, 1H), 7.52–7.59 (m, 3H), 7.74 (d, *J* = 8.0 Hz, 1H), 7.95 (dt, *J* = 2.0 and 11.5 Hz, 1H), 8.05 (d, *J* = 2.0 Hz, 1H), 8.46–8.49 (m, 2H), 8.83 (d, *J* = 2.0 Hz, 1H), 10.06 (s, 1H); δ_C_ (125 MHz, DMSO-*d*_6_) 24.9, 57.1, 78.6, 101.2, 109.0 (d, ^2^*J*_CF_ = 25.6 Hz), 110.5 (d, ^2^*J*_CF_ = 20.8 Hz), 115.5, 117.8, 117.9 (d, ^4^*J*_CF_ = 2.8 Hz), 119.1, 128.2, 128.3, 128.7, 130.3 (d, ^3^*J*_CF_ = 9.5 Hz), 131.1, 137.8, 138.9, 140.9 (d, ^3^*J*_CF_ = 11.3 Hz), 150.9, 157.3, 159.5, 162.2 (d, ^1^*J*_CF_ = 239.0 Hz); *m*/*z* 462 (100, MH^+^); HRMS (ES): MH^+^, found 462.0620. C_24_H_18_BrFN_3_O^+^ requires 462.0617.

### 3.5. Typical Procedure for the One-Pot Three-Step Sequential Amination and Bis-Suzuki-Miyaura Cross-Coupling of ***1a**–**d***

*6-(4-Fluorophenyl)-N-(3-fluorophenyl)-8-(4-methoxyphenyl)-2-phenylquinazolin-4-amine* (**5a**). A stirred mixture of **1a** (0.50 g, 1.12 mmol), 3-fluoroaniline (0.14 g, 1.23 mmol) and concentrated HCl (0.01 g, 0.27 mmol) in 3:1 THF-isopropanol (*v*/*v*, 10 mL) in a two necked flask equipped with rubber septum and a condenser fitted with calcium chloride tube was heated at 70 °C for 5 h. After cooling, 4-methoxyphenylboronic acid (0.20 g, 1.34 mmol), Pd(PPh_3_)_4_ (0.064 g, 0.056 mmol) and K_2_CO_3_ (0.46 g, 3.36 mmol) were introduced to the mixture under argon atmosphere. A balloon filled with argon was fitted to the top of the condenser and the stirred mixture was heated at 70 °C for 3 h. A solution of 4-fluorophenylboronic acid (0.20 g, 1.45 mmol) in THF (5 mL) was then introduced via a syringe and heating was continued at this temperature under argon atmosphere for additional 3 h. The mixture was, in turn, quenched with an ice-cold water and the product was extracted with ethyl acetate. The combined organic layers were washed with water, dried over anhydrous MgSO_4_, filtered and evaporated under reduced pressure. The residue was purified by column chromatography on silica gel to afford **5a** as a yellow solid (0.43 g, 75%), *R*_f_ (toluene) 0.41, mp. 206–207 °C; ν_max_ (ATR) 500, 521, 617, 699, 811, 830, 1147, 1249, 1475, 1488, 1511, 1527, 1567, 1605, 2838, 2958, 3060, 3417 cm^−1^; δ_H_ (500 MHz, DMSO-*d*_6_) 3.86 (s, 3H), 7.01 (td, *J* = 2.5 and 8.5 Hz, 1H), 7.12 (d, *J* = 9.0 Hz, 2H), 7.41 (t, *J* = 8.5 Hz, 2H), 7.48–7.54 (m, 4H), 7.80 (dd, *J* = 2.0 and 8.5 Hz, 1H), 7.86 (d, *J* = 8.5 Hz, 2H), 8.00–8.04 (m, 3H), 8.15 (d, *J* = 2.0 Hz, 1H), 8.35 (d, *J* = 8.0 Hz, 2H), 8.78 (d, *J* = 2.0 Hz, 1H), 10.11 (s, 1H); δ_c_ (125 MHz, DMSO-*d*_6_) 55.6, 109.2 (d, ^2^*J*_CF_ = 25.6 Hz), 110.4 (d, ^2^*J*_CF_ = 20.8 Hz), 113.6, 115.1, 116.2 (d, ^2^*J*_CF_ = 21.7 Hz), 118.1 (d, ^4^*J*_CF_ = 2.8 Hz), 119.6, 128.2, 128.9, 129.7, 129.8, 130.4 (d, ^3^*J*_CF_ = 9.5 Hz), 130.8, 131.1, 132.2, 132.5, 136.2 (d, ^4^*J*_CF_ = 2.7 Hz), 136.8, 138.8, 139.5, 141.6 (d, ^3^*J*_CF_ = 11.3 Hz), 147.6, 158.5, 158.7, 159.3, 162.5 (d, ^1^*J*_CF_ = 239.8 Hz), 162.6 (d, ^1^*J*_CF_ = 243.7 Hz); *m*/*z* 516 (100, MH^+^); HRMS (ES): MH^+^, found 516.1895. C_33_H_24_F_2_N_3_O^+^ requires 516.1887.

### 3.6. Typical Procedure for the One-Pot Three-Step Sequential Amination and Subsequent Sonogashira and Stille Cross-Coupling of ***1a**–**d***

*N-(3-Fluorophenyl)-6-(furan-2-yl)-2-phenyl-8-(phenylethynyl)quinazolin-4-amine* (**6a**). A stirred mixture of **1a** (0.30 g, 0.67 mmol), 3-fluoroaniline (0.082 g, 0.74 mmol) and concentrated HCl (0.01 g, 0.27 mmol) in 3:1 THF-isopropanol (*v*/*v*, 10 mL) in a two necked flask equipped with rubber septum and a condenser fitted with calcium chloride tube was heated at 70 °C for 5 h. After cooling to RT, Pd(PPh_3_)_4_ (0.040 g, 0.03 mmol), CuI (0.006 g, 0.033 mmol), K_2_CO_3_ (0.28 g, 2.01 mmol) and phenylacetylene (0.08 g, 0.80 mmol) in THF (5 mL) were introduced to the mixture under argon atmosphere. A balloon filled with argon was connected to the top of the condenser and stirring was continued for additional 18 h at RT under argon atmosphere. A solution of tributylstannaylfuran (0.29 g, 0.80 mmol) in THF (5 mL) was introduced to the mixture via a syringe and the stirred mixture was, in turn, heated at 70 °C for 1 h. The mixture was quenched with an ice-cold water and the product was extracted with ethyl acetate. The combined organic layers were washed with water, dried over anhydrous MgSO_4_, filtered and evaporated under reduced pressure. The residue was purified by column chromatography on silica gel to afford **6a** as a yellow solid (0.16 g, 51%), *R*_f_ (toluene) 0.35, mp. 117–119 °C; ν_max_ (ATR) 519, 590, 675, 688, 754, 861, 983, 1148, 1352, 1399, 1488, 1526, 1562, 1615, 2208, 2852, 2923, 3442 cm^−1^; δ_H_ (500 MHz, DMSO-*d*_6_) 6.72 (dd, *J* = 2.0 and 3.5 Hz, 1H), 7.01 (td, *J* = 2.5 and 8.5 Hz, 1H), 7.26 (d, *J* = 3.5 Hz, 1H), 7.47–7.59 (m, 7H), 7.70 (dd, *J* = 2.0 and 8.0 Hz, 2H), 7.79 (d, *J* = 8.0 Hz, 1H), 7.91 (d, *J* = 2.0 Hz, 1H), 7.96 (dt, *J* = 2.5 and 11.5 Hz, 1H), 8.44 (d, *J* = 2.0 Hz, 1H), 8.51 (dd, *J* = 2.0 and 8.5 Hz, 2H), 8.86 (d, *J* = 2.0 Hz, 1H), 10.23 (s, 1H); δ_c_ (125 MHz, DMSO-*d*_6_) 87.5, 95.9, 108.3, 109.5 (d, ^2^*J*_CF_ = 25.5 Hz), 110.7 (d, ^2^*J*_CF_ = 21.7 Hz), 113.1, 115.0, 117.6, 118.4 (d, ^4^*J*_CF_ = 2.8 Hz), 122.6, 123.1, 128.0, 128.3, 129.1, 129.4, 129.5, 130.5 (d, ^3^*J*_CF_ = 9.5 Hz), 131.1, 131.9, 132.6, 138.3 (d, ^4^*J*_CF_ = 2.8 Hz), 141.4 (d, ^3^*J*_CF_ = 10.5 Hz), 144.3, 150.4, 152.2, 158.4, 159.3, 162.4 (d, ^1^*J*_CF_ = 239.8 Hz); *m*/*z* 482 (100, MH^+^); HRMS (ES): MH^+^, found 482.1670. C_32_H_20_FN_3_O^+^ requires 482.1669.

### 3.7. Typical Procedure for the One-Pot Three-Step Sequential Amination, Sonogashira and Suzuki–Miyaura Cross-Coupling of ***1b**–**d***

*N-(3-Fluorophenyl)-2,6-bis(4-fluorophenyl)-8-(phenylethynyl)quinazolin-4-amine* (**7a**). A stirred mixture of **1b** (0.30 g, 0.67 mmol), 3-fluoroaniline (0.08 g, 0.74 mmol) and concentrated HCl (0.01 g, 0.27 mmol) in 3:1 THF-isopropanol (*v*/*v*, 10 mL) in a two necked flask equipped with rubber septum and a condenser fitted with calcium chloride tube was heated at 70 °C for 5 h. After cooling to RT, Pd(PPh_3_)_4_ (0.04 g, 0.03 mmol), CuI (0.006 g, 0.033 mmol), K_2_CO_3_ (0.28 g, 2.01 mmol) and phenylacetylene (0.08 g, 0.80 mmol) in THF (5 mL) were introduced to the mixture under argon atmosphere. An argon-filled balloon was connected to the top of the condenser and the mixture was stirred for additional 18 h at r.t. under argon atmosphere. A solution of 4-fluorophenylboronic acid (0.11 g, 0.80 mmol) in water (5 mL) was introduced via a syringe and the stirred mixture was, in turn, heated at 70 °C for 2 h followed by quenching with an ice-cold water. The product was extracted with ethyl acetate and the combined organic layers were washed with water, dried over anhydrous MgSO_4_, filtered and evaporated under reduced pressure. The residue was purified by column chromatography on silica gel to afford **7a** as a yellow solid (0.20 g, 59%), *R*_f_ (9:1 toluene/ethyl acetate) 0.51, mp. 225–227 °C; ν_max_ (ATR) 518, 542, 589, 689, 753, 801, 837, 1152, 1207, 1411, 1444, 1508, 1529, 1568, 1596, 1619, 3452 cm^−1^; δ_H_ (500 MHz, DMSO-*d*_6_) 7.03 (td, *J* = 2.5 and 8.0 Hz, 1H), 7.41 (t, *J* = 9.0 Hz, 4H), 7.47–7.51 (m, 4H), 7.70 (d, *J* = 8.0 Hz, 2H), 7.75 (d, *J* = 8.5 Hz, 1H), 7.91 (dt, *J* = 2.5 and 11.5 Hz, 1H), 7.99 (t, *J* = 8.0 Hz, 2H), 8.41 (d, *J* = 1.5 Hz, 1H), 8.54 (t, *J* = 8.5 Hz, 2H), 8.83 (d, *J* = 1.5 Hz, 1H), 10.19 (s, 1H); δ_c_ (125 MHz, DMSO-*d*_6_) 87.8, 95.8, 109.6 (d, ^2^*J*_CF_ = 25.5 Hz), 110.8 (d, ^2^*J*_CF_ = 20.8 Hz), 114.8, 116.0 (d, ^2^*J*_CF_ = 21.8 Hz), 116.3 (d, ^2^*J*_CF_ = 20.8 Hz), 118.5 (d, ^4^*J*_CF_ = 1.9 Hz), 121.5, 122.5, 123.1, 129.4, 129.5, 129.7, 130.0, 130.5 (d, ^3^*J*_CF_ = 9.4 Hz), 130.6 (d, ^3^*J*_CF_ = 11.3 Hz), 131.9, 134.9 (d, ^4^*J*_CF_ = 2.8 Hz), 135.3 (d, ^4^*J*_CF_ = 3.7 Hz), 135.7, 136.7, 141.2 (d, ^3^*J*_CF_ = 10.3 Hz), 158.6, 158.7, 162.4 (d, ^1^*J*_CF_ = 232.3 Hz), 162.7 (d, ^1^*J*_CF_ = 243.6 Hz), 164.4 (d, ^1^*J*_CF_ = 239.0 Hz); *m*/*z* 528 (100, MH^+^); HRMS (ES): MH^+^, found 528.1693. C_34_H_21_F_3_N_3_^+^ requires 528.1688.

### 3.8. Materials and Methods for In Vitro Cytotoxicity Assays

Human breast adenocarcinoma (MCF-7) cells, human cervical cancer (HeLa) and human lung carcinoma (A549) cell lines obtained from Cellonex were maintained in Dulbecco’s Modified Eagle′s (DMEM, HyClone, Thermo Scientific, Aalst, Belgium) supplemented with 0.4 mM l-glutamine, sodium pyruvate and 10% foetal bovine serum (FBS, HyClone, Thermo Scientific). The cells of a sub-confluent culture were harvested using trypsin-EDTA (HyClone, Thermo Scientific) and centrifuged at 200× *g* for 5 min and re-suspended in growth medium to 5 × 10^4^ cells/mL. A total of 200 µL of the cell suspension was pipetted into each well of columns 2 to 11 of a 96 well culture plate. The same amount of the growth medium was added to wells of column 1 and 12 to maintain humidity and minimize the edge effect. The plates were incubated at 37 °C in a 5% CO_2_ incubator overnight until the cells were in the exponential phase of growth. After incubation, the DMEM was aspirated from the cells and replaced with 200 µL of different concentrations of the test samples (0.1–100 µg/mL). Each dilution of the test sample was tested in quadruplicate in each experiment and the experiments were repeated three times. The plates were again incubated for 2 days at 37 °C in a 5% incubator. Untreated cells and cells treated with different concentrations of doxorubicin hydrochloride (Sigma-Aldrich, GmBH, Schnelldorf, Germany) were included as negative control and as positive control, respectively. After incubation, 30 µL of 5 mg/mL MTT (Sigma) in phosphate buffered saline PBS was added to each well and the plates were incubated for a further 4 h at 37 °C. After incubation with MTT, the medium in each well was removed and the formazan crystals formed were dissolved by adding 50 µL of DMSO to each well of the plates. The plates were gently shaken until the crystals were dissolved. The amount of MTT reduction was measured immediately by detecting the absorbance using a BioTek microplate reader (BioTek Synergy, Analytical and Diagnostic Products, Johannesburg, South Africa) at a wavelength of 570 nm. The wells in column 1 and 12, which contained medium and MTT but no cells was used to blank the microplate reader. The percentage of cell viability was calculated using the formula below:

(1) $$\%\mathrm{Cell}\;\mathrm{viability}\;=\frac{\mathrm{Mean}\;\mathrm{Absorbance}\;\mathrm{of}\;\mathrm{sample}}{\mathrm{Mean}\;\mathrm{Absorbance}\;\mathrm{of}\;\mathrm{control}}\;\times \;100$$

The LC_50_ values (lethal concentration at which 50% of the cells are killed) were calculated as the concentration of the test sample that resulted in 50% reduction of absorbance compared to untreated cells. The intensity of the MTT formazan produced by living metabolically active cells is directly proportional to the number of live cells present [28].

### 3.9. Flow Cytometric Analysis of Apoptotic Cells

Apoptotic cells were quantified using flow cytometry. Briefly, MCF-7, HeLa and A549 cells were cultured in 6 well plates and treated with compounds **2c**, **3c** and **7a**, respectively. The concentration ranges were chosen from the LC_50_ concentration obtained in 4.8. After the cells were induced for 48 h, both treated and untreated cells were harvested, washed two times with ice PBS and adjusted at a density of 1 × 10^6^ cells/sample. The cells were stained with Annexin-V-FLOUS staining kit (Roche, GmBH, Mannheim, Germany) according to the manufacturer's instructions. The cells were analysed using Becton, Dickinson and Company FACS Accuri flow cytometer (Erembodegem, Belgium).

### 3.10. Caspase-3 Activation Assay

Caspase-3 activity was detected by using the Caspase-3 Colorimetric Assay Kit (CASP-3-C Sigma). Briefly, cells were cultured in 96 well plates and treated for 24 h. After treatments, cells were lysed on ice and experiments were carried out according to the manufacturer’s instructions. The concentration of the released pNA from the substrate is calculated by measuring absorbance at 405 nm by using the BioTek microplate reader (BioTek Synergy, Analytical and Diagnostic Products, Johannesburg, South Africa). The caspase-3 activity of each sample was expressed in μmol pNA released per min per mL of cell lysate.

## 4. Conclusions

In summary, series of novel unsymmetrical polycarbo-substituted 4-anilinoquinazolines were generated from the 2-aryl-6-bromo-4-chloro-8-iodoquinazolines via single-pot sequential multiple transformations involving initial nucleophilic substitution (S_N_AR) and subsequent bis-Suzuki-Miyaura, Sonogashira/Stille or Sonogashira/Suzuki-Miyaura cross-coupling. In each case, the one-pot cross-coupling steps were accomplished with the use of a single catalyst complex by just varying the reaction time and temperature for the subsequent step. The methodology represents an attractive alternative to classical multistep synthesis for the easy assembly of readily available building blocks into more complex derivatives. The 4-anilinoquinolines and their polycarbo-substituted derivatives were evaluated for potential in vitro cytotoxicity against the breast cancer (MCF-7), human cervical cancer (HeLa) and human lung carcinoma (A549) cell lines. The most active compounds **2b**, **2c**, **3c**, **4a**, **4c** and **5a** are more selective against the MCF-7 and HeLa cell lines than the human lung carcinoma (A549) cells. The SAR, based on these preliminary in vitro cytotoxicity results, reveals that the presence of bromine and iodine atoms on the 6- and 8-position and a 2-(4-halogenophenyl)-group favour activity against MCF-7 cell line. Substitution of iodine with 4-methoxyphenyl or 2-hydroxybutyn-1-yl enhances activity against the HeLa cell line for the 6-bromo-substituted derivatives bearing phenyl (**4a** and **5a**) or 4-chlorophenyl (**3c** and **4c**) substituent at the 2-position of the 4-anilinoquinazoline framework. Compound **2c** bearing the two halogen atoms on the fused benzo ring appears to induce apoptosis in MCF-7 cells as confirmed by the results from flow cytometry. Replacement of one or both halogen atoms with a carbon-based substitutent, on the hand, resulted in significant necrosis for **3c**. Even though the flow cytometry results were inconclusive for compound **7a**, treatment of lung cancer cells with this compound resulted in caspase-3 activation. The preliminary in vitro cytotoxicity results and SAR described herein, form a basis for the design and synthesis of more potent 2-arylquinazolines decorated with different halogen atoms on the 4-aniline moiety and various carbon-based substituents on the fused benzo ring. This will probably reveal the exact mode of action of these compounds (apoptosis vs. necrosis) depending on the substitution pattern and presumably the type of cell line.

## Figures and Tables

**Table 1:** Percentage yield of compounds **2a–d.**

Compound	R	%Yield of 2
**2a**	4-H	92
**2b**	4-F	89
**2c**	4-Cl	94
**2d**	4-OMe	90

**Table 2:** Percentage yield of compounds **3a**–**d**.

Compound	R	%Yield of 3
**3a**	4-H	82
**3b**	4-F	74
**3c**	4-Cl	79
**3d**	4-OMe	80

**Table 3:** Percentage yield of compounds **4a–d**.

Compound	R	%Yield of 4
**4a**	4-H	63
**4b**	4-F	72
**4c**	4-Cl	68
**4d**	4-OMe	59

**Table 4:** Percentage yield of compounds **5a–d**.

Compound	R	%Yield of 5
**5a**	4-H	75
**5b**	4-F	76
**5c**	4-Cl	68
**5d**	4-OMe	66

**Table 5:** Percentage yield of compounds **6a**–**d**.

Compound	R	%Yield of 6
**6a**	4-H	51
**6b**	4-F	60
**6c**	4-Cl	53
**6d**	4-OMe	65

**Table 6:** Percentage yield of compounds **7a**–**c.**

Compound	R	%Yield of 7
**7a**	4-F	59
**7b**	4-Cl	56
**7c**	4-OCH_3_	62

**Table 7:**
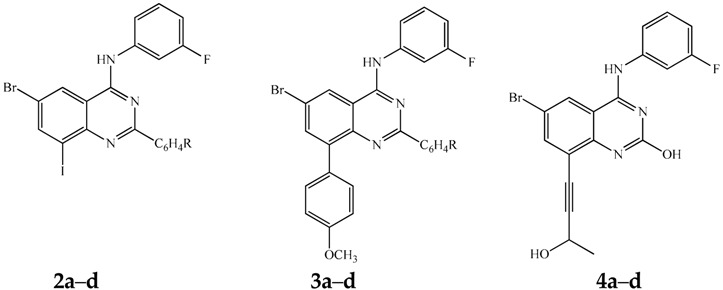
Cytotoxic effects of **2a**–**d**, **3a**–**d** and **4a**–**d** against MCF-7, HeLa and A549 cell lines.

Compound	LC_50_ (μM) ± SD		
	MCF-7	HeLa	A549
**2a** (R = 4-H)	11.59 ± 1.54	19.21 ± 1.49	14.34 ± 1.93
**2b** (R = 4-F)	3.12 ± 0.31	8.53 ± 0.23	9.72 ± 0.45
**2c** (R = 4-Cl)	2.96 ± 0.16	14.27 ± 0.09	19.45 ± 0.24
**2d** (R = 4-OMe)	>100	34.71 ± 1.25	99.01 ± 0.72
**3a** (R = 4-H)	>100	>100	>100
**3b** (R = 4-F)	16.56 ± 0.43	19.51 ± 0.25	1.67 ± 1.92
**3c** (R = 4-Cl)	6.55 ± 0.38	˂0.10	10.88 ± 1.35
**3d** (R = 4-OMe)	13.75 ± 0.70	22.54 ± 0.97	>100
**4a** (R = 4-H)	24.30 ± 0.33	6.29 ± 0.92	11.49 ± 0.04
**4b** (R = 4-F)	11.00 ± 1.60	15.27 ± 0.51	15.35 ± 0.59
**4c** (R = 4-Cl)	8.41 ± 0.77	1.73 ± 0.16	9.35 ± 0.75
**4d** ((R = 4-OMe)	24.14 ± 1.33	7.48 ± 0.37	>100
Doxorubicin hydrochloride	1.62 ± 0.034	1.66 ± 0.03	2.75 ± 0.33

**Table 8:**
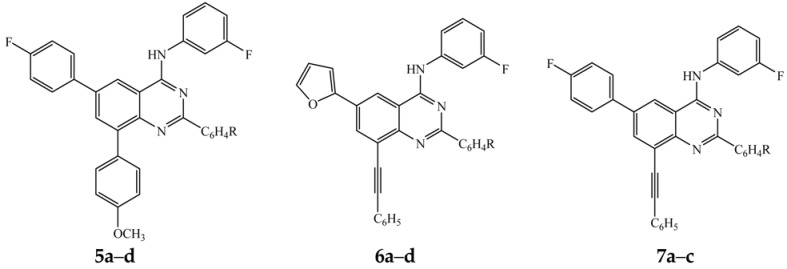
Cytotoxic effects of **5a**–**d**, **6a**–**d** and **7a**–**c** against MCF-7, HeLa and A549 cell lines.

Compound	LC_50_ (μM) ± SD		
	MCF-7	HeLa	A549
**5a** (R = 4-H)	>100	1.45 ± 0.29	>100
**5b** (R = 4-F)	>100	25.35 ± 1.76	71.64 ± 1.33
**5c** (R = 4-Cl)	62.14 ± 0.58	>100	>100
**5d** (R = 4-OMe)	>100	>100	>100
**6b** (R = 4-F)	54.76 ± 1.78	15.14 ± 0.60	>100
**6c** (R = 4-Cl)	>100	>100	>100
**6d** (R = 4-OMe)	>100	>100	>100
**7a** (R = 4-F)	9.48 ± 0.11	15.49 ± 0.74	6.04 ± 0.28
**7b** (R = 4-Cl)	>100	>100	>100
**7c** (R = 4-OMe)	16.64 ± 0.49	>100	>100
Doxorubicin hydrochloride	1.62 ± 0.034	1.66 ± 0.03	2.75 ± 0.33

**Table 9:** Quantitative apoptosis assay of A549 using Annexin-V/PI dual staining method. The percentage of viable cells, early apoptotic cells, late apoptotic cells, and necrotic cells are presented as mean ± SD.

Group/Concentration	Q1	Q2	Q3	Q4
MCF-7				
Negative control	5.95 ± 007	16.80 ± 0.28	76.25 ± 1.77	0.25 ± 0.35
Positive control 3 µM	27.50 ± 0.35	62.5 ± 3.53	12.5 ± 0.35	1.65 ± 0.49
**Compound 2c**				
3 µM	9.10 ± 0.77	67.1 ± 0.64	22.9 ± 0.78	0.8 ± 0.14
1.5 µM	25.5 ± 0.23	48.5 ± 0.21	25.0 ± 1.41	1.0 ± 0.14
0.75 µM	34.5 ± 0.71	31.2 ± 1.10	32.2 ± 0.31	1.7 ± 0.84
HeLa				
Negative control	27.45 ± 0.78	31.80 ± 0.25	46.00 ± 0.56	0.05 ± 0.07
Positive control 3 µM	40.40 ± 1.09	58.5 ± 0.21	1.55 ± 0.64	0.95 ± 0.07
**Compound 3c**				
10 µM	52.7 ± 0.92	24.6 ± 0.28	22.5 ± 0.35	0.1 ± 0.03
1 µM	50.15 ± 0.34	39.155 ± 1.20	12.85 ± 1.22	0.05 ± 0.01
0.1 µM	39.15 ± 1.20	49.8 ± 0.25	9.95 ± 0.10	0.15 ± 0.07
**A549**				
Negative control	52.8 ± 0.28	30.8 ± 0.28	16.45 ± 0.64	0.15 ± 0.07
Positive control 3 µM	70.0 ± 1.41	28.4 ± 0.84	0.38 ± 0.07	0.0 ± 0.00
**Compound 7a**				
10 µM	26.9 ± 0.10	4.1 ± 0.78	68.9 ± 0.78	0.1 ± 0.28
5 µM	39.7 ± 0.42	9.8 ± 1.13	51.0 ± 1.41	0.05 ± 0.07
2.5 µM	49.85 ± 0.21	15.2 ± 1.70	36.95 ± 0.43	0.15 ± 0.07

## Supplementary Materials

Supplementary materials can be accessed at: http://www.mdpi.com/1420-3049/21/10/1366/s1. Experimental procedures, copies of NMR spectra, and the percentage of cell viability and LC_50_ values of doxorubicin hydrochloride and compounds **2**–**7**.
