# Supplementary material for: Synthesis and In Vitro Cytotoxic Properties of Polycarbo-Substituted 4-(Arylamino)quinazolines
Source: Molecules. 2016 Oct 14;21(10):1366. doi: 10.3390/molecules21101366 (PMC6274161; doi:10.3390/molecules21101366)
Supplement: Supplementary file 1 [file molecules-21-01366-s001.pdf]

Supplementary Materials: Synthesis and In Vitro Cytotoxic Properties of Polycarbo-Substituted 4-(Arylamino)quinazolines

Hugues Kamdem Paumo, Tshepiso Jan Makhafola and Malose Jack Mphahlele

1. Detailed Experimental Section

1.1. Generalised Procedure for the Aminination of **1a–d**

A stirred mixture of **1** (1 equiv.), 3-fluoroaniline (1.1 equiv.) and concentrated HCl (0.01 g, 0.27 mmol) in 3:1 THF-isopropanol (5 mL/mmol of **1**) was heated at 70 °C for 5 h and then allowed to cool to RT. The reaction mixture was quenched with an ice-cold water and the product was extracted into ethyl acetate. The organic layers were washed with an aqueous solution of $NaHCO_3$, dried over anhydrous $MgSO_4$, filtered and evaporated under reduced pressure to afford **2** as a solid. The following products (**2a–d**) were prepared in this fashion:

Bromo-N-(3-fluorophenyl)-8-iodo-2-phenylquinazolin-4-amine (**2a**): Yellow solid (1.07 g, 92%), mp. 199–200 °C; ν_{max} (ATR) 445, 551, 701, 770, 957, 1128, 1306, 1397, 1448, 1541, 1590, 1616, 3424 cm^{-1}; δ_H (500 MHz, DMSO-*d*$_6$) 7.02 (td, *J* = 2.5 and 8.0 Hz, 1H), 7.50 (q, *J* = 8.0 Hz, 1H), 7.55–7.58 (m, 3H), 7.75 (d, *J* = 8.5 Hz, 1H), 7.95 (dt, *J* = 2.0 and 11.5 Hz, 1H), 8.48–8.49 (m, 2H), 8.57 (d, *J* = 1.5 Hz, 1H), 8.89 (d, *J* = 1.5 Hz, 1H), 10.13 (s, 1H); δ_C (125 MHz, DMSO-*d*$_6$) 105.2, 109.3 (d, $^2J_{CF}$ = 26.5 Hz), 110.8 (d, $^2J_{CF}$ = 20.9 Hz), 115.7, 118.2 (d, $^4J_{CF}$ = 2.8 Hz), 119.4, 126.3, 128.5, 129.1, 130.6 (d, $^3J_{CF}$ = 8.5 Hz), 131.5, 137.9, 141.2 (d, $^3J_{CF}$ = 11.3 Hz), 145.2, 149.2, 157.9, 160.5, 162.4 (d, $^1J_{CF}$ = 239.8 Hz); *m*/*z* 520 (100, MH$^+$); HRMS (ES): MH$^+$, found 519.9230. $C_{20}H_{13}BrFIN_3^+$ requires 519.9243.

6-Bromo-N-(3-fluorophenyl)-2-(4-fluorophenyl)-8-iodoquinazolin-4-amine (**2b**): Yellow solid (1.03 g, 89%), mp. 248–249 °C; ν_{max} (ATR) 446, 522, 559, 682, 709, 794, 961, 1145, 1303, 1395, 1444, 1546, 1586, 1611, 3452 cm^{-1}; δ_H (500 MHz, DMSO-*d*$_6$) 7.00 (td, *J* = 2.0 and 8.0 Hz, 1H), 7.37 (t, *J* = 2.0 and 9.0 Hz, 2H), 7.48 (q, *J* = 8.0 Hz, 1H), 7.71 (dd, *J* = 1.5 and 8.5 Hz, 1H), 7.86 (dt, *J* = 2.5 and 11.5 Hz, 1H), 8.46 (t, *J* = 9.0 Hz, 2H), 8.51 (d, *J* = 2.0 Hz, 1H), 8.82 (d, *J* = 2.0 Hz, 1H), 10.08 (s, 1H); δ_C (125 MHz, DMSO-*d*$_6$) 105.0, 109.3 (d, $^2J_{CF}$ = 26.6 Hz), 110.9 (d, $^2J_{CF}$ = 20.9 Hz), 115.5, 116.0 (d, $^2J_{CF}$ = 21.7 Hz), 118.2 (d, $^4J_{CF}$ = 2.8 Hz), 119.3, 126.3, 130.5 (d, $^3J_{CF}$ = 9.3 Hz), 130.7 (d, $^3J_{CF}$ = 9.5 Hz), 134.3 (d, $^4J_{CF}$ = 2.8 Hz), 141.1 (d, $^3J_{CF}$ = 11.3 Hz), 145.1, 149.0, 157.9, 159.4, 162.4 (d, $^1J_{CF}$ = 239.8 Hz), 164.5 (d, $^1J_{CF}$ = 246.5 Hz); *m*/*z* 539 (100, MH$^+$); HRMS (ES): MH$^+$, found 538.9250. $C_{20}H_{12}BrF_2IN_3^+$ requires 538.9129.

6-Bromo-2-(4-chlorophenyl)-N-(3-fluorophenyl)-8-iodoquinazolin-4-amine (**2c**): Yellow solid (1.08 g, 94%), mp. 236–237 °C; ν_{max} (ATR) 438, 480, 550, 669, 701, 775, 964, 1131, 1299, 1387, 1458, 1542, 1587, 1602, 3428 cm^{-1}; δ_H (500 MHz, DMSO-*d*$_6$) 7.02 (td, *J* = 2.5 and 8.0 Hz, 1H), 7.49 (q, *J* = 8.5 Hz, 1H), 7.63 (d, *J* = 8.5 Hz, 2H), 7.72 (dd, *J* = 1.5 and 8.0 Hz, 1H), 7.86 (dt, *J* = 2.5 and 11.5 Hz, 1H), 8.43 (d, *J* = 8.5 Hz, 2H), 8.56 (d, *J* = 2.0 Hz, 1H), 8.82 (d, *J* = 2.0 Hz, 1H), 10.14 (s, 1H); δ_C (125 MHz, DMSO-*d*$_6$) 105.1, 109.4 (d, $^2J_{CF}$ = 25.6 Hz), 110.9 (d, $^2J_{CF}$ = 20.9 Hz), 115.8, 118.3 (d, $^4J_{CF}$ = 2.8 Hz), 119.5, 126.4, 129.2, 130.1, 130.5 (d, $^3J_{CF}$ = 8.6 Hz), 136.3, 136.8, 141.0 (d, $^3J_{CF}$ = 10.3 Hz), 145.2, 149.1, 158.0, 159.5, 162.4 (d, $^1J_{CF}$ = 239.8 Hz); *m*/*z* 555 (100, MH$^+$); HRMS (ES): MH$^+$, found 554.9409. $C_{20}H_{12}BrClFIN_3^+$ requires 554.9407.

6-Bromo-N-(3-fluorophenyl)-8-iodo-2-(4-methoxyphenyl)quinazolin-4-amine (**2d**): Yellow solid (1.04 g, 90%), mp. 181–183 °C; ν_{max} (ATR) 444, 551, 675, 762, 792, 959, 1128, 1164, 1242, 1408, 1458, 1542, 1588, 1609, 2830, 2933, 3413 cm^{-1}; δ_H (500 MHz, DMSO-*d*$_6$) 3.83 (s, 3H), 6.98 (td, *J* = 2.5 and 8.5 Hz, 1H), 7.08 (d, *J* = 8.5 Hz, 2H), 7.47 (q, *J* = 8.5 Hz, 1H), 7.72 (dd, *J* = 1.5 and 8.0 Hz, 1H), 7.92 (dt, *J* = 2.5 and 11.5 Hz, 1H), 8.39 (d, *J* = 9.0 Hz, 2H), 8.49 (d, *J* = 2.0 Hz, 1H), 8.81 (d, *J* = 2.0 Hz, 1H), 10.01 (s, 1H); δ_C (125 MHz, DMSO-*d*$_6$) 55.8, 104.9, 109.1 (d, $^2J_{CF}$ = 26.3 Hz), 110.7 (d, $^2J_{CF}$ = 20.6 Hz), 114.4, 115.3, 118.1 (d, $^4J_{CF}$ = 2.8 Hz), 118.7, 126.2, 130.2, 130.3, 130.5 (d, $^3J_{CF}$ = 8.6 Hz), 141.2 (d, $^3J_{CF}$ = 10.3 Hz), 144.9, 149.2, 157.7, 160.3, 162.1, 162.4 (d, $^1J_{CF}$ = 239.3 Hz); *m*/*z* 550 (100, MH$^+$); HRMS (ES): MH$^+$, found 549.9417. $C_{21}H_{15}BrFIN_3O^+$ requires 549.9427.

1.2 Generalised Procedure for the One-Pot Two-Step Sequential Amination and Suzuki-Miyaura Cross-Coupling of **1a–d**

A stirred mixture of **1a** (0.50 g, 1.12 mmol), 3-fluoroaniline (0.14 g, 1.23 mmol) and concentrated HCl (0.01 g, 0.27 mmol) in 3:1 THF-isopropanol (*v*/*v*, 10 mL) was heated at 70 °C for 5 h until the starting material was completely consumed (thin layer chromatography monitoring) and then cooled to RT. The cooled mixture was treated sequentially with $Pd(PPh_3)_4$ (0.064 g, 0.056 mmol) and a solution of K_2CO_3 (0.46 g, 3.36 mmol) in water (5 mL) followed by purging with argon gas for 30 min. A solution of 4-methoxyphenylboronic acid (0.20 g, 1.34 mmol) in THF (5 mL) was then injected using a syringe. The reaction mixture was stirred at 70 °C for additional 3 h and then quenched with an ice-cold water. The product was extracted into ethyl acetate and the combined organic layers were washed with water, dried over $MgSO_4$, filtered and evaporated under reduced pressure. The residue was purified by column chromatography on silica gel to afford **3a** as a solid. Compounds **3b–d** were also prepared in this fashion from the corresponding substrates.

6-Bromo-N-(3-fluorophenyl)-8-(4-methoxyphenyl)-2-phenylquinazolin-4-amine (**3a**): Yellow solid (0.45 g, 82%), R_f (toluene) 0.40, mp. 210–211 °C; ν_{max} (ATR) 450, 501, 565, 676, 717, 773, 829, 975, 1061, 1147, 1289, 1351, 1395, 1440, 1557, 1607, 2846, 3018, 3426 cm^{-1}; δ_H (500 MHz, DMSO-*d*$_6$) 3.86 (s, 3H), 7.00 (td, *J* = 2.0 and 8.5 Hz, 1H), 7.10 (d, *J* = 8.5 Hz, 2H), 7.50 (m, 4H), 7.77 (d, *J* = 8.5 Hz, 2H), 7.79 (d, *J* = 8.5 Hz, 1H), 7.97 (d, *J* = 2.5 Hz, 1H), 8.00 (dt, *J* = 2.0 and 8.5 Hz, 1H), 8.30–8.32 (m, 2H), 8.82 (d, *J* = 2.0 Hz, 1H), 10.04 (s, 1H); δ_C (125 MHz, DMSO-*d*$_6$) 55.6, 109.1 (d, $^2J_{CF}$ = 26.6 Hz), 110.3 (d, $^2J_{CF}$ = 20.8 Hz), 113.7, 116.2, 118.0 (d, $^4J_{CF}$ = 2.7 Hz), 119.0, 124.5, 128.3, 129.0, 129.3, 129.7, 130.5 (d, $^3J_{CF}$ = 9.5 Hz), 131.0, 132.5, 135.9, 138.5, 141.3 (d, $^3J_{CF}$ = 10.3 Hz), 147.3, 157.7, 158.9, 159.5, 162.5 (d, $^1J_{CF}$ = 239.0 Hz); *m*/*z* 500 (100, MH$^+$); HRMS (ES): MH$^+$, found 500.0780. $C_{27}H_{20}BrFN_3O^+$ requires 500.0774.

6-Bromo-N-(3-fluorophenyl)-2-(4-fluorophenyl)-8-(4-methoxyphenyl)-quinazolin-4-amine (**3b**): Yellow solid (0.41 g, 74%), R_f (toluene) 0.56, mp. 215–217 °C; ν_{max} (ATR) 556, 565, 576, 673, 752, 762, 825, 1162, 1249, 1419, 1489, 1507, 1533, 1559, 1610, 2836, 2922, 3459 cm^{-1}; δ_H (500 MHz, DMSO-*d*$_6$) 3.81 (s, 3H), 7.00 (td, *J* = 2.0 and 8.0 Hz, 1H), 7.05 (d, *J* = 9.0 Hz, 2H), 7.35 (t, *J* = 9.0 Hz, 2H), 7.49 (q, *J* = 8.0 Hz, 1H), 7.77 (d, *J* = 8.0 Hz, 1H), 7.82 (t, *J* = 8.0 Hz, 2H), 7.96–7.99 (m, 2H), 8.22 (d, *J* = 8.5 Hz, 2H), 8.83 (d, *J* = 2.0 Hz, 1H), 10.01 (s, 1H); δ_C (125 MHz, DMSO-*d*$_6$) 55.7, 109.0 (d, $^2J_{CF}$ = 25.5 Hz), 110.4 (d, $^2J_{CF}$ = 20.9 Hz), 114.4, 115.0 (d, $^2J_{CF}$ = 21.7 Hz), 115.9, 118.0 (d, $^4J_{CF}$ = 2.8 Hz), 118.3, 125.1, 130.0, 130.5 (d, $^3J_{CF}$ = 9.5 Hz), 130.8, 133.2 (d, $^3J_{CF}$ = 9.5 Hz), 133.8 (d, $^4J_{CF}$ = 2.7 Hz), 136.2, 140.1, 141.5 (d, $^3J_{CF}$ = 11.3 Hz), 147.4, 157.5, 159.0, 161.8, 162.4 (d, $^1J_{CF}$ = 243.6 Hz), 162.5 (d, $^1J_{CF}$ = 240.0 Hz); *m*/*z* 518 (100, MH$^+$); HRMS (ES): MH$^+$, found 518.0681. $C_{27}H_{19}BrF_2N_3O^+$ requires 518.0680.

6-Bromo-2-(4-chlorophenyl)-N-(3-fluorophenyl)-8-(4-methoxyphenyl)quinazolin-4-amine (**3c**): Yellow solid (0.44 g, 79%), R_f (toluene) 0.57, mp. 245–246 °C; ν_{max} (ATR) 488, 565, 745, 770, 801, 832, 1012, 1086, 1145, 1246, 1348, 1404, 1463, 1489, 1510, 1529, 1555, 1606, 2841, 3423 cm^{-1}; δ_H (500 MHz, DMSO-*d*$_6$) 3.85 (s, 3H), 7.00 (td, *J* = 2.0 and 8.0 Hz, 1H), 7.08 (d, *J* = 8.0 Hz, 2H), 7.49 (q, *J* = 8.0 Hz, 1H), 7.56 (d, *J* = 8.5 Hz, 2H), 7.73 (d, *J* = 8.5 Hz, 2H), 7.76 (dd, *J* = 1.5 and 8.0 Hz, 1H), 7.91 (dt, *J* = 2.5 and 11.5 Hz, 1H), 7.97 (d, *J* = 2.5 Hz, 1H), 8.26 (d, *J* = 8.5 Hz, 2H), 8.80 (d, *J* = 2.5 Hz, 1H), 10.06 (s, 1H); δ_C (125 MHz, DMSO-*d*$_6$) 55.6, 109.2 (d, $^2J_{CF}$ = 26.3 Hz), 110.6 (d, $^2J_{CF}$ = 20.6 Hz), 113.7, 116.2, 118.3 (d, $^4J_{CF}$ = 2.8 Hz), 119.2, 124.5, 129.1, 129.6, 129.9, 130.5 (d, $^3J_{CF}$ = 9.1 Hz), 132.4, 135.8, 136.0, 137.4, 141.2, 141.3 (d, $^3J_{CF}$ = 13.7 Hz), 147.2, 157.8, 158.0, 159.5, 162.4 (d, $^1J_{CF}$ = 239.3 Hz); *m*/*z* 534 (100, MH$^+$); HRMS (ES): MH$^+$, found 534.0399. $C_{27}H_{19}BrClFN_3O^+$ requires 534.0384.

6-Bromo-N-(3-fluorophenyl)-2,8-bis(4-methoxyphenyl)quinazolin-4-amine (**3d**): Yellow solid (0.45 g, 80%), R_f (toluene) 0.26, mp. 212–213 °C; ν_{max} (ATR) 521, 576, 752, 804, 831, 1164, 1239, 1346, 1418, 1509, 1526, 1559, 1592, 1608, 2837, 2936, 3006, 3406 cm^{-1}; δ_H (500 MHz, DMSO-*d*$_6$) 3.79 (s, 3H), 3.84 (s, 3H), 6.98 (td, *J* = 2.0 and 8.0 Hz, 1H), 7.02 (d, *J* = 8.5 Hz, 2H), 7.07 (d, *J* = 9.0 Hz, 2H), 7.47 (q, *J* = 8.0 Hz, 1H), 7.73 (d, *J* = 9.0 Hz, 2H), 7.76 (dd, *J* = 1.5 and 8.0 Hz, 1H), 7.91 (d, *J* = 1.5 Hz, 1H), 7.97 (dt, *J* = 2.5 and 11.5 Hz, 1H), 8.23 (d, *J* = 9.0 Hz, 2H), 8.76 (d, *J* = 1.5 Hz, 1H), 9.96 (s, 1H); δ_C (125 MHz, DMSO-*d*$_6$) 55.6, 55.7, 109.0 (d, $^2J_{CF}$ = 25.6 Hz), 110.3 (d, $^2J_{CF}$ = 20.8 Hz), 113.7, 114.3, 115.9, 117.9 (d, $^4J_{CF}$ = 2.7 Hz), 118.4, 124.4, 129.7, 129.9, 130.5 (d, $^3J_{CF}$ = 9.5 Hz), 130.9, 132.4, 135.7, 141.0, 141.5 (d, $^3J_{CF}$ = 10.3 Hz), 147.4, 157.5,

158.8, 159.5, 161.8, 162.4 (d, $^1J_{CF}$ = 239.8 Hz); *m*/*z* 530 (100, MH^+); HRMS (ES): MH^+, found 530.0891. $C_{28}H_{22}BrFN_3O_2^+$ requires 530.0879.

1.3. A General Procedure for the One-Pot Two-Step Sequential Amination and Sonogashira Cross-Coupling of **1a–d**

A stirred mixture of **1a** (0.40 g, 0.89 mmol), 3-fluoroaniline (0.11 g, 0.98 mmol) and concentrated HCl (0.01 g, 0.27 mmol) in 3:1 THF-isopropanol (v/v, 10 mL) in a two necked flask equipped with rubber septum and a condenser fitted with calcium chloride tube was heated at 70 °C for 5 h. After cooling, $Pd(PPh_3)_4$ (0.051 g, 0.044 mmol), CuI (0.008 g, 0.044 mmol) K_2CO_3 (0.37 g, 2.67 mmol) and 3-butyn-2-ol (0.081 g, 1.15 mmol) in THF (5 mL) were introduced to the mixture under argon atmosphere. A balloon filled with argon was fitted to the top of the condenser and the mixture was stirred for additional 18 h at RT under argon atmosphere. The mixture was quenched with an ice-cold water and the product was extracted into ethyl acetate. The combined organic layers were washed with water, dried over anhyd. $MgSO_4$, filtered and evaporated under reduced pressure. The residue was purified by column chromatography on silica gel to afford **4a**. Compounds **4b–d** were also prepared from the corresponding substrates in this fashion.

4-[6-Bromo-4-(3-fluorophenylamino)-2-phenylquinazolin-8-yl]but-3-yn-2-ol (**4a**): Orange solid (0.26 g, 63%), R_f (9:1 toluene-ethyl acetate) 0.26, mp. 172–174 °C; ν_{max} (ATR) 539, 697, 712, 782, 861, 1071, 1142, 1368, 1470, 1524, 1556, 1612, 2924, 3070, 3308, 3478 cm^{-1}; δ_H (500 MHz, DMSO-*d*$_6$) 1.51 (d, *J* = 6.5 Hz, 3H), 4.76 (d, *J* = 6.0 Hz, 1H), 5.60 (d, *J* = 6.0 Hz, 1H), 7.00 (td, *J* = 2.5 and 8.0 Hz, 1H), 7.51 (q, *J* = 8.0 Hz, 1H), 7.52–7.59 (m, 3H), 7.74 (d, *J* = 8.0 Hz, 1H), 7.95 (dt, *J* = 2.0 and 11.5 Hz, 1H), 8.05 (d, *J* = 2.0 Hz, 1H), 8.46–8.49 (m, 2H), 8.83 (d, *J* = 2.0 Hz, 1H), 10.06 (s, 1H); δ_C (125 MHz, DMSO-*d*$_6$) 24.9, 57.1, 78.6, 101.2, 109.0 (d, $^2J_{CF}$ = 25.6 Hz), 110.5 (d, $^2J_{CF}$ = 20.8 Hz), 115.5, 117.8, 117.9 (d, $^4J_{CF}$ = 2.8 Hz), 119.1, 128.2, 128.3, 128.7, 130.3 (d, $^3J_{CF}$ = 9.5 Hz), 131.1, 137.8, 138.9, 140.9 (d, $^3J_{CF}$ = 11.3 Hz), 150.9, 157.3, 159.5, 162.2 (d, $^1J_{CF}$ = 239.0 Hz); *m*/*z* 462 (100, MH^+); HRMS (ES): MH^+, found 462.0620. $C_{24}H_{18}BrFN_3O^+$ requires 462.0617.

4-[6-Bromo-2-(4-fluorophenyl)-4-(3-fluorophenylamino)quinazolin-8-yl]but-3-yn-2-ol (**4b**): Orange solid (0.29 g, 72%), R_f (9:1 toluene-ethyl acetate) 0.30, mp. 194–196 °C; ν_{max} (ATR) 414, 581, 675, 769, 803, 844, 1098, 1147, 1221, 1350, 1471, 1527, 1558, 1611, 3073, 3360, 3471 cm^{-1}; δ_H (500 MHz, DMSO-*d*$_6$) 1.52 (d, *J* = 7.0 Hz, 3H), 4.76 (p, *J* = 6.0 Hz, 1H), 5.61 (d, *J* = 6.0 Hz, 1H), 7.01 (td, *J* = 2.0 and 8.0 Hz, 1H), 7.37 (t, *J* = 8.5 Hz, 2H), 7.49 (q, *J* = 8.5 Hz, 1H), 7.73 (d, *J* = 8.0 Hz, 1H), 7.89 (dt, *J* = 2.5 and 11.5 Hz, 1H), 8.05 (d, *J* = 2.0 Hz, 1H), 8.50 (t, *J* = 8.0 Hz, 2H), 8.82 (d, *J* = 2.0 Hz, 1H), 10.07 (s, 1H); δ_C (125 MHz, DMSO-*d*$_6$) 25.1, 57.4, 78.9, 101.4, 109.3 (d, $^2J_{CF}$ = 25.6 Hz), 110.8 (d, $^2J_{CF}$ = 19.9 Hz), 115.7, 116.0 (d, $^2J_{CF}$ = 21.7 Hz), 118.1 (d, $^4J_{CF}$ = 2.8 Hz), 118.3, 124.3, 126.1, 130.6 (d, $^3J_{CF}$ = 9.5 Hz), 130.8 (d, $^3J_{CF}$ = 8.5 Hz), 134.6 (d, $^4J_{CF}$ = 2.7 Hz), 139.2, 141.0 (d, $^3J_{CF}$ = 10.3 Hz), 150.1, 157.6, 158.8, 162.4 (d, $^1J_{CF}$ = 239.8 Hz), 164.4 (d, $^1J_{CF}$ = 247.5 Hz); *m*/*z* 480 (100, MH^+); HRMS (ES): MH^+, found 480.0526. $C_{24}H_{17}BrF_2N_3O^+$ requires 480.0523.

4-[6-Bromo-2-(4-chlorophenyl)-4-(3-fluorophenylamino)quinazolin-8-yl]but-3-yn-2-ol (**4c**): Orange solid (0.28 g, 68%), R_f (9:1 toluene-ethyl acetate) 0.55, mp. 211–213 °C; ν_{max} (ATR) 486, 676, 741, 768, 802, 842, 1086, 1099, 1142, 1348, 1365, 1466, 1488, 1528, 1557, 1611, 3073, 3350, 3467 cm^{-1}; δ_H (500 MHz, DMSO-*d*$_6$) 1.52 (d, *J* = 7.0 Hz, 3H), 4.76 (p, *J* = 6.0 Hz, 1H), 5.61 (d, *J* = 6.0 Hz, 1H), 7.02 (td, *J* = 2.5 and 8.5 Hz, 1H), 7.50 (q, *J* = 8.0 Hz, 1H), 7.62 (d, *J* = 8.5 Hz, 2H), 7.74 (dd, *J* = 1.5 and 8.5 Hz, 1H), 7.88 (dt, *J* = 2.5 and 12.0 Hz, 1H), 8.07 (d, *J* = 2.0 Hz, 1H), 8.45 (d, *J* = 8.0 Hz, 2H), 8.84 (d, *J* = 2.0 Hz, 1H), 10.12 (s, 1H); δ_C (125 MHz, DMSO-*d*$_6$) 25.1, 57.4, 78.8, 101.5, 109.3 (d, $^2J_{CF}$ = 25.6 Hz), 110.9 (d, $^2J_{CF}$ = 20.7 Hz), 115.8, 118.3, 118.4 (d, $^4J_{CF}$ = 2.8 Hz), 124.3, 126.1, 129.1, 130.1, 130.6 (d, $^3J_{CF}$ = 9.5 Hz), 141.0 (d, $^3J_{CF}$ = 10.3 Hz), 136.2, 137.0, 139.3, 150.1, 157.6, 158.8, 162.4 (d, $^1J_{CF}$ = 239.8 Hz); *m*/*z* 496 (100, MH^+); HRMS (ES): MH^+, found 496.0220. $C_{24}H_{17}BrClFN_3O^+$ requires 497.0228.

4-[6-Bromo-4-(3-fluorophenylamino)-2-(4-methoxyphenyl)quinazolin-8-yl]but-3-yn-2-ol (**4d**): Yellow solid (0.24 g, 59%), R_f (9:1 toluene-ethyl acetate) 0.20, mp. 163–164 °C; ν_{max} (ATR) 412, 536, 582, 674, 768, 799, 839, 869, 1028, 1144, 1162, 1242, 1348, 1362, 1418, 1488, 1528, 1588, 1608, 2926, 3069, 3359 cm^{-1}; δ_H (500 MHz, DMSO-*d*$_6$) 1.52 (d, *J* = 7.0 Hz, 3H), 3.84 (s, 3H), 4.76 (p, *J* = 5.5 Hz, 1H), 5.62 (d, *J* = 5.0 Hz, 1H), 7.00 (td, *J* = 1.5 and 8.5 Hz, 1H), 7.09 (d, *J* = 9.0 Hz, 2H), 7.49 (q, *J* = 8.0 Hz, 1H), 7.74 (dd, *J* = 2.0

and 8.5 Hz, 1H), 7.92 (dt, *J* = 2.5 and 11.5 Hz, 1H), 8.03 (d, *J* = 2.0 Hz, 1H), 8.43 (d, *J* = 9.0 Hz, 2H), 8.81 (d, *J* = 2.5 Hz, 1H), 10.02 (s, 1H); δ_C (125 MHz, DMSO-*d*$_6$) 25.1, 55.8, 57.4, 79.0, 101.2, 109.1 (d, $^2J_{CF}$ = 25.6 Hz), 110.6 (d, $^2J_{CF}$ = 20.8 Hz), 114.4, 115.5, 117.4, 118.1 (d, $^4J_{CF}$ = 1.9 Hz), 124.1, 126.1, 128.6, 130.2, 130.5 (d, $^3J_{CF}$ = 7.6 Hz), 139.1, 141.2 (d, $^3J_{CF}$ = 10.3 Hz), 150.3, 157.4, 159.6, 162.1, 162.4 (d, $^1J_{CF}$ = 239.8 Hz); *m*/*z* 492 (100, MH$^+$); HRMS (ES): MH$^+$, found 492.0724. $C_{25}H_{20}BrFN_3O_2^+$ requires 492.0723.

1.4. Generalised Procedure for the One-Pot Three-Step Sequential Amination and Bis-Suzuki–Miyaura Cross-Coupling of **1a**–**d**

A stirred mixture of **1a** (0.50 g, 1.12 mmol), 3-fluoroaniline (0.14 g, 1.23 mmol) and concentrated HCl (0.01 g, 0.27 mmol) in 3:1 THF-isopropanol (*v*/*v*, 10 mL) in a two necked flask equipped with rubber septum and a condenser fitted with calcium chloride tube was heated at 70 °C for 5 h. After cooling, 4-methoxyphenylboronic acid (0.20 g, 1.34 mmol), $Pd(PPh_3)_4$ (0.064 g, 0.056 mmol) and K_2CO_3 (0.46 g, 3.36 mmol) were introduced to the mixture under argon atmosphere. A balloon filled with argon was fitted to the top of the condenser and the stirred mixture was heated at 70 °C for 3 h. A solution of 4-fluorophenylboronic acid (0.20 g, 1.45 mmol) in THF (5 mL) was then introduced via a syringe and heating was continued at this temperature under argon atmosphere for additional 3 h. The mixture was, in turn, quenched with an ice-cold water and the product was extracted into ethyl acetate. The combined organic layers were washed with water, dried over anhydrous $MgSO_4$, filtered and evaporated under reduced pressure. The residue was purified by column chromatography on silica gel to afford **5a**. Compounds **5b**–**d** were also prepared in this fashion from their corresponding substrates.

6-(4-Fluorophenyl)-N-(3-fluorophenyl)-8-(4-methoxyphenyl)-2-phenylquinazolin-4-amine (**5a**): Yellow solid (0.43 g, 75%), *R*$_f$ (toluene) 0.41, mp. 206–207 °C; ν_{max} (ATR) 500, 521, 617, 699, 811, 830, 1147, 1249, 1475, 1488, 1511, 1527, 1567, 1605, 2838, 2958, 3060, 3417 cm^{-1}; δ_H (500 MHz, DMSO-*d*$_6$) 3.86 (s, 3H), 7.01 (td, *J* = 2.5 and 8.5 Hz, 1H), 7.12 (d, *J* = 9.0 Hz, 2H), 7.41 (t, *J* = 8.5 Hz, 2H), 7.48–7.54 (m, 4H), 7.80 (dd, *J* = 2.0 and 8.5 Hz, 1H), 7.86 (d, *J* = 8.5 Hz, 2H), 8.00–8.04 (m, 3H), 8.15 (d, *J* = 2.0 Hz, 1H), 8.35 (d, *J* = 8.0 Hz, 2H), 8.78 (d, *J* = 2.0 Hz, 1H), 10.11 (s, 1H); δ_c (125 MHz, DMSO-*d*$_6$) 55.6, 109.2 (d, $^2J_{CF}$ = 25.6 Hz), 110.4 (d, $^2J_{CF}$ = 20.8 Hz), 113.6, 115.1, 116.2 (d, $^2J_{CF}$ = 21.7 Hz), 118.1 (d, $^4J_{CF}$ = 2.8 Hz), 119.6, 128.2, 128.9, 129.7, 129.8, 130.4 (d, $^3J_{CF}$ = 9.5 Hz), 130.8, 131.1, 132.2, 132.5, 136.2 (d, $^4J_{CF}$ = 2.7 Hz), 136.8, 138.8, 139.5, 141.6 (d, $^3J_{CF}$ = 11.3 Hz), 147.6, 158.5, 158.7, 159.3, 162.5 (d, $^1J_{CF}$ = 239.8 Hz), 162.6 (d, $^1J_{CF}$ = 243.7 Hz); *m*/*z* 516 (100, MH$^+$); HRMS (ES): MH$^+$, found 516.1895. $C_{33}H_{24}F_2N_3O^+$ requires 516.1887.

N-(3-Fluorophenyl)-2,6-bis(4-fluorophenyl)-8-(4-methoxyphenyl)quinazolin-4-amine (**5b**): Brown solid (0.44 g, 76%), *R*$_f$ (toluene) 0.42, mp. 227–228 °C; ν_{max} (ATR) 437, 527, 587, 750, 804, 824, 1141, 1181, 1213, 1247, 1507, 1574, 1596, 1617, 2926, 2956, 3460 cm^{-1}; δ_H (500 MHz, DMSO-*d*$_6$) 3.84 (s, 3H), 7.00 (td, *J* = 2.5 and 8.0 Hz, 1H), 7.09 (d, *J* = 8.5 Hz, 2H), 7.32 (t, *J* = 8.5 Hz, 2H), 7.38 (t, *J* = 8.5 Hz, 2H), 7.49 (q, *J* = 8.0 Hz, 1H), 7.75 (d, *J* = 8.0 Hz, 1H), 7.82 (d, *J* = 8.5 Hz, 2H), 7.93 (dt, *J* = 2.5 and 11.5 Hz, 1H), 7.99 (t, *J* = 8.0 Hz, 2H), 8.11 (d, *J* = 1.5 Hz, 1H), 8.34 (t, *J* = 8.0 Hz, 2H), 8.75 (d, *J* = 1.5 Hz, 1H), 10.10 (s, 1H); δ_c (125 MHz, DMSO-*d*$_6$) 55.6, 109.0 (d, $^2J_{CF}$ = 25.6 Hz), 110.5 (d, $^2J_{CF}$ = 20.8 Hz), 113.6, 115.0, 115.9 (d, $^2J_{CF}$ = 20.8 Hz), 116.2 (d, $^2J_{CF}$ = 20.8 Hz), 118.3 (d, $^4J_{CF}$ = 2.7 Hz), 119.7, 129.7 (d, $^3J_{CF}$ = 7.6 Hz), 130.4 (d, $^3J_{CF}$ = 8.5 Hz), 130.5 (d, $^3J_{CF}$ = 9.5 Hz), 131.0, 132.3, 132.5, 135.2 (d, $^4J_{CF}$ = 2.7 Hz) 136.2 (d, $^4J_{CF}$ = 2.6 Hz), 136.9, 139.5, 141.5 (d, $^3J_{CF}$ = 11.3 Hz), 147.5, 157.6, 158.7, 159.2, 162.5 (d, $^1J_{CF}$ = 240.0 Hz), 162.6 (d, $^1J_{CF}$ = 243.6 Hz), 164.1 (d, $^1J_{CF}$ = 245.6 Hz); *m*/*z* 534 (100, MH$^+$); HRMS (ES): MH$^+$, found 534.1793. $C_{33}H_{23}F_3N_3O^+$ requires 534.1798.

2-(4-Chlorophenyl)-N-(3-fluorophenyl)-6-(4-fluorophenyl)-8-(4-methoxyphenyl) quinazolin-4-amine (**5c**): Brown solid (0.38 g, 68%), *R*$_f$ (toluene) 0.54, mp. 213–214 °C; ν_{max} (ATR) 433, 521, 528, 553, 745, 803, 825, 1142, 1214, 1246, 1396, 1489, 1508, 1563, 1602, 2925, 2956, 3441 cm^{-1}; δ_H (500 MHz, DMSO-*d*$_6$) 3.86 (s, 3H), 7.00 (td, *J* = 2.5 and 8.5 Hz, 1H), 7.09 (d, *J* = 8.5 Hz, 2H), 7.39 (t, *J* = 9.0 Hz, 2H), 7.50 (q, *J* = 8.5 Hz, 1H), 7.56 (d, *J* = 9.0 Hz, 2H), 7.75 (d, *J* = 8.0 Hz, 1H), 7.82 (d, *J* = 9.0 Hz, 2H), 7.92 (dt, *J* = 2.5 and 11.5 Hz, 1H), 8.00 (d, *J* = 9.0 Hz, 2H), 8.13 (d, *J* = 2.5 Hz, 1H), 8.30 (d, *J* = 8.0 Hz, 2H), 8.75 (d, *J* = 2.5 Hz, 1H), 10.11 (s, 1H); δ_c (125 MHz, DMSO-*d*$_6$) 55.6, 109.3 (d, $^2J_{CF}$ = 25.6 Hz), 110.6 (d, $^2J_{CF}$ = 20.8 Hz), 113.7, 115.1, 116.2 (d, $^2J_{CF}$ = 20.7 Hz), 118.3 (d, $^4J_{CF}$ = 2.8 Hz), 119.6, 129.0, 129.7, 129.8, 129.8, 130.5 (d, $^3J_{CF}$ = 9.1

Hz), 131.0, 132.3, 132.5, 135.6, 136.2 (d, $^4J_{CF}$ = 2.7 Hz), 137.7, 139.5, 141.5 (d, $^3J_{CF}$ = 11.3 Hz), 147.4, 157.6, 158.7, 159.3, 162.5 (d, $^1J_{CF}$ = 239.8 Hz), 162.6 (d, $^1J_{CF}$ = 243.7 Hz); *m*/*z* 550 (100, MH^+); HRMS (ES): MH^+, found 550.1516. $C_{33}H_{23}ClF_2N_3O^+$ requires 550.1498.

N-(3-Fluorophenyl)-6-(4-fluorophenyl)-2,8-bis(4-methoxyphenyl)quinazolin-4-amine (**5d**): Brown solid (0.37 g, 66%), R_f (9:1 toluene-ethyl acetate) 0.71, mp. 196–197 °C; ν_{max} (ATR) 520, 591, 770, 802, 822, 1033, 1157, 1244, 1401, 1415, 1506, 1604, 2834, 2905, 2932, 3454 cm^{-1}; δ_H (500 MHz, DMSO-d_6) 3.80 (s, 3H), 3.85 (s, 3H), 6.99 (td, *J* = 1.5 and 8.5 Hz, 1H), 7.04 (d, *J* = 8.5 Hz, 2H), 7.09 (d, *J* = 8.5 Hz, 2H), 7.38 (t, *J* = 8.5 Hz, 2H), 7.49 (q, *J* = 8.0 Hz, 1H), 7.77 (dd, *J* = 1.5 and 8.0 Hz, 1H), 7.83 (d, *J* = 8.5 Hz, 2H), 7.97–8.00 (m, 3H), 8.09 (d, *J* = 1.5 Hz, 1H), 8.27 (d, *J* = 9.0 Hz, 2H), 8.73 (d, *J* = 1.5 Hz, 1H), 10.03 (s, 1H); δ_C (125 MHz, DMSO-d_6) 55.6, 55.7, 109.1 (d, $^2J_{CF}$ = 25.6 Hz), 110.3 (d, $^2J_{CF}$ = 20.8 Hz), 113.6, 114.3, 114.8, 116.2 (d, $^2J_{CF}$ = 21.8 Hz), 118.1 (d, $^4J_{CF}$ = 2.7 Hz), 119.6, 129.7, 129.8, 129.9, 130.4 (d, $^3J_{CF}$ = 9.5 Hz), 131.2 (d, $^3J_{CF}$ = 11.3 Hz), 132.2, 132.5, 136.3 (d, $^4J_{CF}$ = 2.8 Hz), 136.4, 139.2, 141.7 (d, $^3J_{CF}$ = 10.3 Hz), 147.5, 158.4, 158.5, 159.2, 161.6, 162.5 (d, $^1J_{CF}$ = 239.8 Hz), 162.6 (d, $^1J_{CF}$ = 243.6 Hz); *m*/*z* 546 (100, MH^+); HRMS (ES): MH^+, found 546.1992. $C_{33}H_{24}F_2N_3O^+$ requires 546.1993.

1.5. Generalised Procedure for the One-Pot Three-Step Sequential Amination and Subsequent Sonogashira and Stille Cross-Coupling of **1a–d**

A stirred mixture of **1a** (0.30 g, 0.67 mmol), 3-fluoroaniline (0.082 g, 0.74 mmol) and concentrated HCl (0.01 g, 0.27 mmol) in 3:1 THF-isopropanol (*v*/*v*, 10 mL) in a two necked flask equipped with rubber septum and a condenser fitted with calcium chloride tube was heated at 70 °C for 5 h. After cooling to RT, $Pd(PPh_3)_4$ (0.040 g, 0.03 mmol), CuI (0.006 g, 0.033 mmol), K_2CO_3 (0.28 g, 2.01 mmol) and phenylacetylene (0.08 g, 0.80 mmol) in THF (5 mL) were introduced to the mixture under argon atmosphere. A balloon filled with argon was connected to the top of the condenser and stirring was continued for additional 18 h at r.t. under argon atmosphere. A solution of tributylstannaylfuran (0.29 g, 0.80 mmol) in THF (5 mL) was introduced to the mixture via a syringe and the stirred mixture was, in turn, heated at 70 °C for 1 h. The mixture was quenched with an ice-cold water and the product was extracted into ethyl acetate. The combined organic layers were washed with water, dried over anhydrous $MgSO_4$, filtered and evaporated under reduced pressure. The residue was purified by column chromatography on silica gel to afford **6a**. Compounds **6a–d** were also prepared in this fashion.

N-(3-Fluorophenyl)-6-(furan-2-yl)-2-phenyl-8-(phenylethynyl)quinazolin-4-amine (**6a**): Yellow solid (0.16 g, 51%), R_f (toluene) 0.35, mp. 117–119 °C; ν_{max} (ATR) 519, 590, 675, 688, 754, 861, 983, 1148, 1352, 1399, 1488, 1526, 1562, 1615, 2208, 2852, 2923, 3442 cm^{-1}; δ_H (500 MHz, DMSO-d_6) 6.72 (dd, *J* = 2.0 and 3.5 Hz, 1H), 7.01 (td, *J* = 2.5 and 8.5 Hz, 1H), 7.26 (d, *J* = 3.5 Hz, 1H), 7.47–7.59 (m, 7H), 7.70 (dd, *J* = 2.0 and 8.0 Hz, 2H), 7.79 (d, *J* = 8.0 Hz, 1H), 7.91 (d, *J* = 2.0 Hz, 1H), 7.96 (dt, *J* = 2.5 and 11.5 Hz, 1H), 8.44 (d, *J* = 2.0 Hz, 1H), 8.51 (dd, *J* = 2.0 and 8.5 Hz, 2H), 8.86 (d, *J* = 2.0 Hz, 1H), 10.23 (s, 1H); δ_C (125 MHz, DMSO-d_6) 87.5, 95.9, 108.3, 109.5 (d, $^2J_{CF}$ = 25.5 Hz), 110.7 (d, $^2J_{CF}$ = 21.7 Hz), 113.1, 115.0, 117.6, 118.4 (d, $^4J_{CF}$ = 2.8 Hz), 122.6, 123.1, 128.0, 128.3, 129.1, 129.4, 129.5, 130.5 (d, $^3J_{CF}$ = 9.5 Hz), 131.1, 131.9, 132.6, 138.3 (d, $^4J_{CF}$ = 2.8 Hz), 141.4 (d, $^3J_{CF}$ = 10.5 Hz), 144.3, 150.4, 152.2, 158.4, 159.3, 162.4 (d, $^1J_{CF}$ = 239.8 Hz); m/z 482 (100, MH^+); HRMS (ES): MH^+, found 482.1670. $C_{32}H_{20}FN_3O^+$ requires 482.1669.

N-(3-Fluorophenyl)-2-(4-fluorophenyl)-6-(furan-2-yl)-8-(phenylethynyl)quinazolin-4-amine (**6b**): Yellow solid (0.19 g, 60%), R_f (toluene) 0.41, mp. 175–178 °C; ν_{max} (ATR) 449, 521, 571, 590, 675, 690, 733, 757, 984, 1018, 1147, 1218, 1397, 1488, 1509, 1525, 1567, 1616, 3053, 3437 cm^{-1}; δ_H (500 MHz, DMSO-d_6) 6.71 (dd, *J* = 1.5 and 3.5 Hz, 1H), 7.01 (td, *J* = 2.0 and 8.0 Hz, 1H), 7.25 (d, *J* = 3.5 Hz, 1H), 7.39 (t, *J* = 9.0 Hz, 2H), 7.44–7.54 (m, 4H), 7.70 (dd, *J* = 2.0 and 8.0 Hz, 2H), 7.76 (dd, *J* = 2.0 and 8.0 Hz, 1H), 7.90 (d, *J* = 2.0 Hz, 1H), 7.91 (dt, *J* = 2.5 and 11.5 Hz, 1H), 8.43 (d, *J* = 2.0 Hz, 1H), 8.51 (t, *J* = 8.5 Hz, 2H), 8.85 (d, *J* = 2.0 Hz, 1H), 10.23 (s, 1H); δ_C (125 MHz, DMSO-d_6) 87.4, 95.9, 108.3, 109.6 (d, $^2J_{CF}$ = 26.5 Hz), 110.8 (d, $^2J_{CF}$ = 20.8 Hz), 113.1, 114.9, 116.0 (d, $^2J_{CF}$ = 21.8 Hz), 117.6, 118.5 (d, $^4J_{CF}$ = 2.7 Hz), 122.6, 123.0, 128.0, 129.4, 129.5, 130.5 (d, $^3J_{CF}$ = 9.5 Hz), 130.6 (d, $^3J_{CF}$ = 9.5 Hz), 131.9, 132.5 (d, $^4J_{CF}$ = 2.8 Hz), 134.8 (d, $^4J_{CF}$ = 2.8 Hz), 141.3 (d, $^3J_{CF}$ = 10.5 Hz), 144.3, 150.3, 152.2, 158.5, 158.6, 162.4 (d, $^1J_{CF}$ = 239.8 Hz), 164.3 (d, $^1J_{CF}$ = 246.6 Hz); *m*/*z* 500 (100, MH^+); HRMS (ES): MH^+, found 500.1575. $C_{32}H_{20}F_2N_3O^+$ requires 500.1574.

2-(4-Chlorophenyl)-N-(3-fluorophenyl)-6-(furan-2-yl)-8-(phenylethynyl)quinazolin-4-amine (**6c**): Yellow solid (017 g, 53%), R_f (toluene) 0.42, mp. 212–214 °C; ν_{max} (ATR) 491, 588, 674, 683, 740, 753, 800, 1013, 1086, 1147, 1349, 1394, 1488, 1526, 1559, 1583, 1615, 2211, 3447 cm^{-1}; δ_H (500 MHz, DMSO-d_6) 6.72 (dd, J = 2.0 and 3.5 Hz, 1H), 7.02 (td, J = 2.0 and 8.0 Hz, 1H), 7.25 (d, J = 3.5 Hz, 1H), 7.46–7.55 (m, 4H), 7.63 (d, J = 8.0 Hz, 2H), 7.70 (dd, J = 2.0 and 8.0 Hz, 2H), 7.77 (dd, J = 2.0 and 8.0 Hz, 1H), 7.90 (dt, J = 2.5 and 11.5 Hz, 1H), 7.91 (d, J = 2.0 Hz, 1H), 8.43 (d, J = 2.0 Hz, 1H), 8.47 (d, J = 9.0 Hz, 2H), 8.85 (d, J = 2.0 Hz, 1H), 10.25 (s, 1H); δ_C (125 MHz, DMSO-d_6) 87.4, 95.9, 108.4, 109.6 (d, $^2J_{CF}$ = 25.6 Hz), 110.8 (d, $^2J_{CF}$ = 20.8 Hz), 113.1, 115.0, 117.6, 118.5 (d, $^4J_{CF}$ = 2.7 Hz), 122.6, 123.0, 128.1, 128.6, 129.2, 129.4, 129.9, 130.5 (d, $^3J_{CF}$ = 9.5 Hz), 131.9, 132.7 (d, $^4J_{CF}$ = 2.8 Hz), 135.9, 137.2, 141.3 (d, $^3J_{CF}$ = 10.3 Hz), 144.3, 150.3, 152.2, 158.4, 158.5, 162.4 (d, $^1J_{CF}$ = 239.8 Hz); m/z 516 (100, MH$^+$); HRMS (ES): MH$^+$, found 516.1200. $C_{32}H_{19}ClFN_3O^+$ requires 516.1201.

N-(3-Fluorophenyl)-6-(furan-2-yl)-2-(4-methoxyphenyl)-8-(phenylethynyl)quinazolin-4-amine (**6d**): Yellow solid (0.20 g, 65%), R_f (9:1 toluene/ethyl acetate) 0.70, mp. 220–223 °C; ν_{max} (ATR) cm^{-1}; δ_H (500 MHz, DMSO-d_6) 3.84 (s, 3H), 6.71 (s, 1H), 7.01 (td, J = 2.5 and 8.5 Hz, 1H), 7.12 (d, J = 8.5 Hz, 2H), 7.23 (s, 1H), 7.48–7.52 (m, 4H), 7.70 (d, J = 8.0 Hz, 2H), 7.77 (d, J = 8.0 Hz, 1H), 7.90 (s, 1H), 7.96 (dt, J = 2.5 and 11.5 Hz, 1H), 8.41 (s, 1H), 8.45 (d, J = 9.0 Hz, 2H), 8.84 (s, 1H), 10.17 (s, 1H); δ_C (125 MHz, DMSO-d_6) 55.8, 87.6, 95.7, 108.0, 109.4 (d, $^2J_{CF}$ = 25.6 Hz), 110.5 (d, $^2J_{CF}$ = 19.8 Hz), 113.0, 114.5, 114.7, 117.7, 118.3 (d, $^4J_{CF}$ = 2.7 Hz), 122.3, 123.1, 127.5, 129.4, 130.0, 130.4, 130.5 (d, $^3J_{CF}$ = 9.5 Hz), 130.8, 131.9, 136.3 (d, $^4J_{CF}$ = 2.8 Hz), 141.4 (d, $^3J_{CF}$ = 10.3 Hz), 144.2, 150.6, 152.3, 158.3, 159.2, 162.0, 162.4 (d, $^1J_{CF}$ = 239.8 Hz); m/z 512 (100, MH$^+$); HRMS (ES): MH$^+$, found 512.1776. $C_{33}H_{23}FN_3O_2^+$ requires 512.1774.

1.6. Generalised Procedure for the One-Pot Three-Step Sequential Amination, Sonogashira and Suzuki–Miyaura Cross-Coupling of **1b–d**

A stirred mixture of **1b** (0.30 g, 0.67 mmol), 3-fluoroaniline (0.08 g, 0.74 mmol) and concentrated HCl (0.01 g, 0.27 mmol) in 3:1 THF-isopropanol (*v*/*v*, 10 mL) in a two necked flask equipped with rubber septum and a condenser fitted with calcium chloride tube was heated at 70 °C for 5 h. After cooling to RT, $Pd(PPh_3)_4$ (0.04 g, 0.03 mmol), CuI (0.006 g, 0.033 mmol), K_2CO_3 (0.28 g, 2.01 mmol) and phenylacetylene (0.08 g, 0.80 mmol) in THF (5 mL) were introduced to the mixture under argon atmosphere. An argon-filled balloon was connected to the top of the condenser and the mixture was stirred for additional 18 h at r.t. under argon atmosphere. A solution of 4-fluorophenylboronic acid (0.11 g, 0.80 mmol) in water (5 mL) was introduced via a syringe and the stirred mixture was, in turn, heated at 70 °C for 2 h followed by quenching with an ice-cold water. The product was extracted into ethyl acetate and the combined organic layers were washed with water, dried over anhydrous $MgSO_4$, filtered and evaporated under reduced pressure. The residue was purified by column chromatography on silica gel to afford **7a**. Compounds **7b** and **7c** were also prepared in this fashion from their corresponding substrates.

N-(3-Fluorophenyl)-2,6-bis(4-fluorophenyl)-8-(phenylethynyl)quinazolin-4-amine (**7a**): Yellow solid (0.20 g, 59%), R_f (toluene) 0.51, mp. 225–227 °C; ν_{max} (ATR) 518, 542, 589, 689, 753, 801, 837, 1152, 1207, 1411, 1444, 1508, 1529, 1568, 1596, 1619, 3452 cm^{-1}; δ_H (500 MHz, DMSO-d_6) 7.03 (td, J = 2.5 and 8.0 Hz, 1H), 7.41 (t, J = 9.0 Hz, 4H), 7.47–7.51 (m, 4H), 7.70 (d, J = 8.0 Hz, 2H), 7.75 (d, J = 8.5 Hz, 1H), 7.91 (dt, J = 2.5 and 11.5 Hz, 1H), 7.99 (t, J = 8.0 Hz, 2H), 8.41 (d, J = 1.5 Hz, 1H), 8.54 (t, J = 8.5 Hz, 2H), 8.83 (d, J = 1.5 Hz, 1H), 10.19 (s, 1H); δ_C (125 MHz, DMSO-d_6) 87.8, 95.8, 109.6 (d, $^2J_{CF}$ = 25.5 Hz), 110.8 (d, $^2J_{CF}$ = 20.8 Hz), 114.8, 116.0 (d, $^2J_{CF}$ = 21.8 Hz), 116.3 (d, $^2J_{CF}$ = 20.8 Hz), 118.5 (d, $^4J_{CF}$ = 1.9 Hz), 121.5, 122.5, 123.1, 129.4, 129.5, 129.7, 130.0, 130.5 (d, $^3J_{CF}$ = 9.4 Hz), 130.6 (d, $^3J_{CF}$ = 11.3 Hz), 131.9, 134.9 (d, $^4J_{CF}$ = 2.8 Hz), 135.3 (d, $^4J_{CF}$ = 3.7 Hz), 135.7, 136.7, 141.2 (d, $^3J_{CF}$ = 10.3 Hz), 158.6, 158.7, 162.4 (d, $^1J_{CF}$ = 232.3 Hz), 162.7 (d, $^1J_{CF}$ = 243.6 Hz), 164.4 (d, $^1J_{CF}$ = 239.0 Hz); m/z 528 (100, MH$^+$); HRMS (ES): MH$^+$, found 528.1693. $C_{34}H_{21}F_3N_3^+$ requires 528.1688.

2-(4-Chlorophenyl)-N-(3-fluorophenyl)-6-(4-fluorophenyl)-8-(phenylethynyl)quinazolin-4-amine (**7b**): Yellow solid (0.19 g, 56%), R_f (toluene) 0.57, mp. 218–219 °C; ν_{max} (ATR) 515, 543, 564, 689, 753, 799, 830, 1013, 1087, 1160, 1234, 1408, 1489, 1510, 1530, 1561, 1602, 1615, 3452 cm^{-1}; δ_H (500 MHz, DMSO-d_6) 7.03 (td, J = 2.5 and 8.5 Hz, 1H), 7.41 (t, J = 9.0 Hz, 2H), 7.47–7.51 (m, 4H), 7.65 (d, J = 8.5 Hz, 2H),

7.70 (d, *J* = 8.0 Hz, 2H), 7.74 (d, *J* = 9.0 Hz, 1H), 7.89 (dt, *J* = 2.5 and 11.5 Hz, 1H), 8.00 (t, *J* = 8.5 Hz, 2H), 8.42 (d, *J* = 1.5 Hz, 1H), 8.49 (d, *J* = 8.5 Hz, 2H), 8.84 (d, *J* = 1.5 Hz, 1H), 10.22 (s, 1H); δ_C (125 MHz, DMSO-d_6) 87.7, 95.9, 109.6 (d, $^2J_{CF}$ = 25.5 Hz), 110.9 (d, $^2J_{CF}$ = 20.8 Hz), 114.9, 116.4 (d, $^2J_{CF}$ = 21.8 Hz), 118.5 (d, $^4J_{CF}$ = 2.7 Hz), 121.5, 122.5, 123.1, 129.2, 129.4, 129.5, 129.7 (d, $^3J_{CF}$ = 8.6 Hz), 130.0, 130.6 (d, $^3J_{CF}$ = 9.5 Hz), 131.9, 135.4 (d, $^4J_{CF}$ = 2.8 Hz), 135.5 (d, $^4J_{CF}$ = 3.7 Hz), 136.0, 136.9, 137.3, 141.1 (d, $^3J_{CF}$ = 11.3 Hz), 150.3, 158.6, 158.7, 162.5 (d, $^1J_{CF}$ = 240.8 Hz), 162.8 (d, $^1J_{CF}$ = 244.6 Hz); *m*/*z* 544 (100, MH^+); HRMS (ES): MH^+, found 544.1393. $C_{34}H_{21}ClF_2N_3^+$ requires 544.1392.

N-(3-Fluorophenyl)-6-(4-fluorophenyl)-2-(4-methoxyphenyl)-8-(phenylethynyl)-quinazolin-4-amine (**7c**): Yellow solid (0.20 g, 62%), R_f (9:1 toluene/ethyl acetate) 0.76, mp. 236–237 °C; ν_{max} (ATR) cm^{-1}; δ_H (500 MHz, DMSO-d_6) 3.84 (s, 3H), 7.01 (td, *J* = 2.5 and 8.5 Hz, 1H), 7.12 (d, *J* = 8.5 Hz, 2H), 7.40 (t, *J* = 8.5 Hz, 2H), 7.47–7.55 (m, 4H), 7.70 (dd, *J* = 1.5 and 8.0 Hz, 2H), 7.75 (d, *J* = 8.0 Hz, 1H), 7.93–8.01 (m, 3H), 8.39 (d, *J* = 2.0 Hz, 1H), 8.47 (d, *J* = 8.5 Hz, 2H), 8.81 (d, *J* = 2.0 Hz, 1H), 10.13 (s, 1H); δ_C (125 MHz, DMSO-d_6) 55.8 87.9, 95.0, 109.4 (d, $^2J_{CF}$ = 25.6 Hz), 110.6 (d, $^2J_{CF}$ = 20.8 Hz), 114.5, 114.6, 116.3 (d, $^2J_{CF}$ = 20.8 Hz), 118.3 (d, $^4J_{CF}$ = 2.7 Hz), 121.5, 122.3, 123.2, 129.4, 129.5, 129.6 (d, $^3J_{CF}$ = 7.6 Hz), 130.1, 130.5 (d, $^3J_{CF}$ = 9.5 Hz), 130.8, 131.9, 135.4 (d, $^4J_{CF}$ = 2.8 Hz), 135.5 (d, $^4J_{CF}$ = 3.7 Hz), 136.2, 141.4 (d, $^3J_{CF}$ = 11.3 Hz), 150.6, 158.4, 159.4, 162.0, 162.5 (d, $^1J_{CF}$ = 239.8 Hz), 162.7 (d, $^1J_{CF}$ = 243.6 Hz); *m*/*z* 540 (100, MH^+); HRMS (ES): MH^+, found 540.1882. $C_{35}H_{24}F_2N_3O^+$ requires 540.1887.

2. ^1H- and ^{13}C NMR Spectra of Compounds 2–7

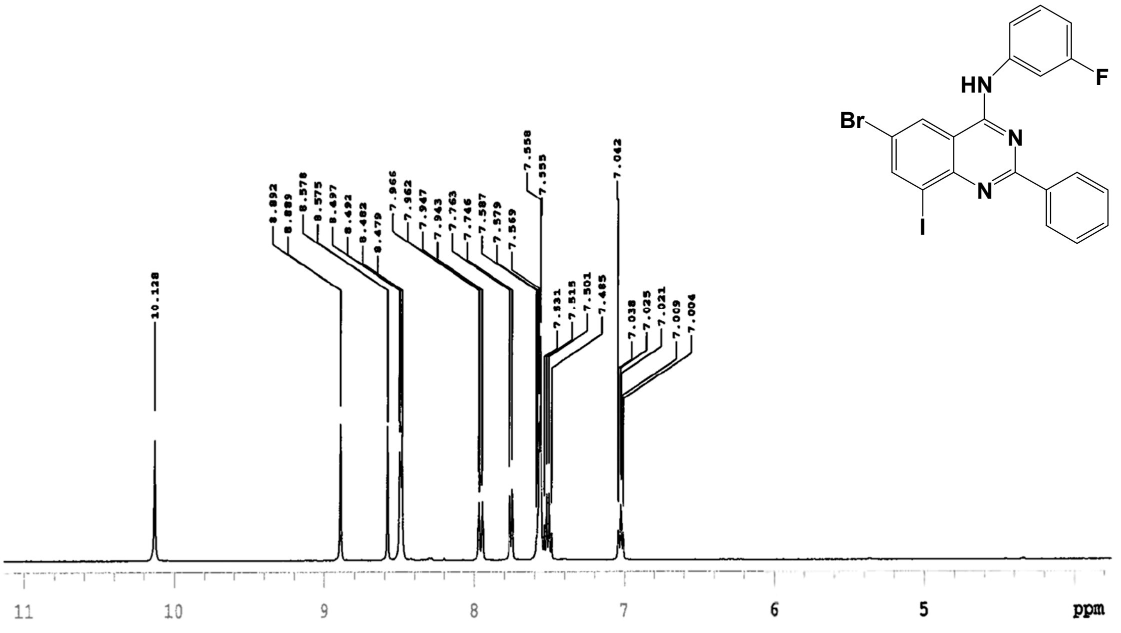

Figure S1. ^1H-NMR Spectrum of Compound **2a.**

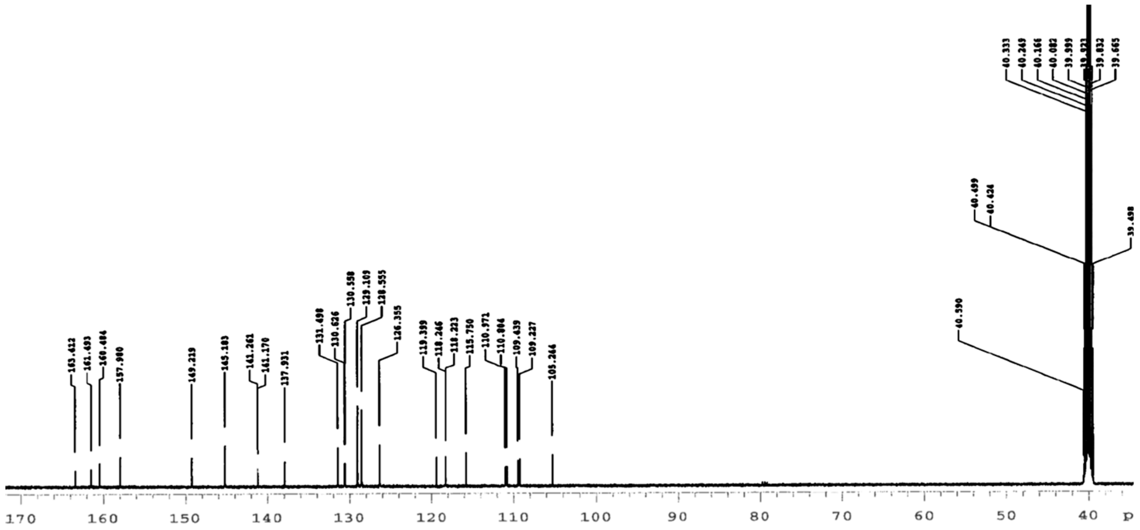

Figure S2. ^{13}C NMR Spectrum of Compound **2a.**

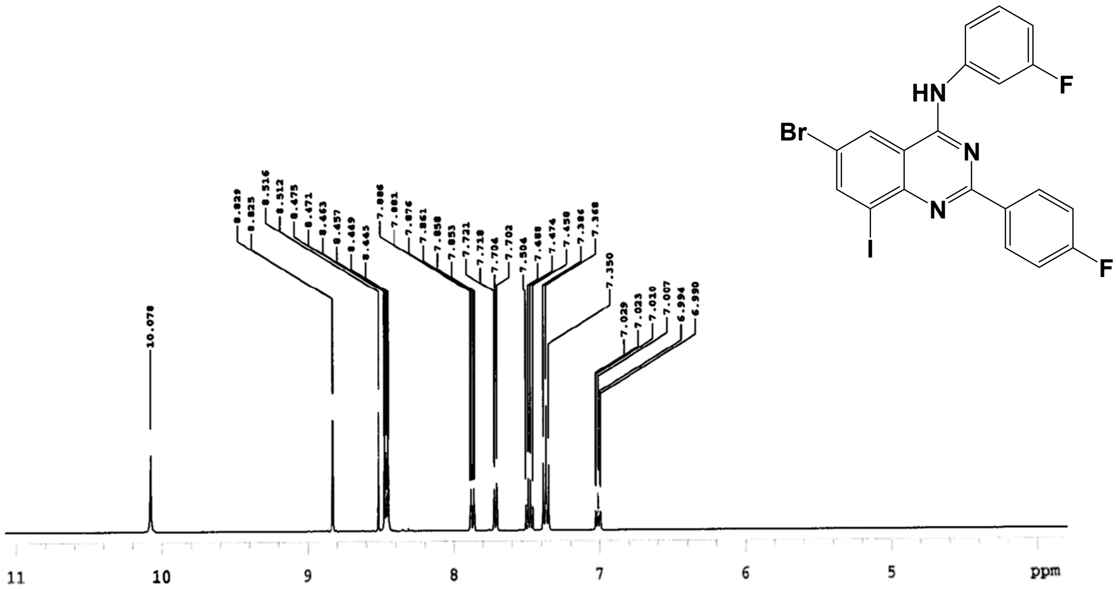

Figure S3. ^{1}H NMR Spectrum of Compound **2b.**

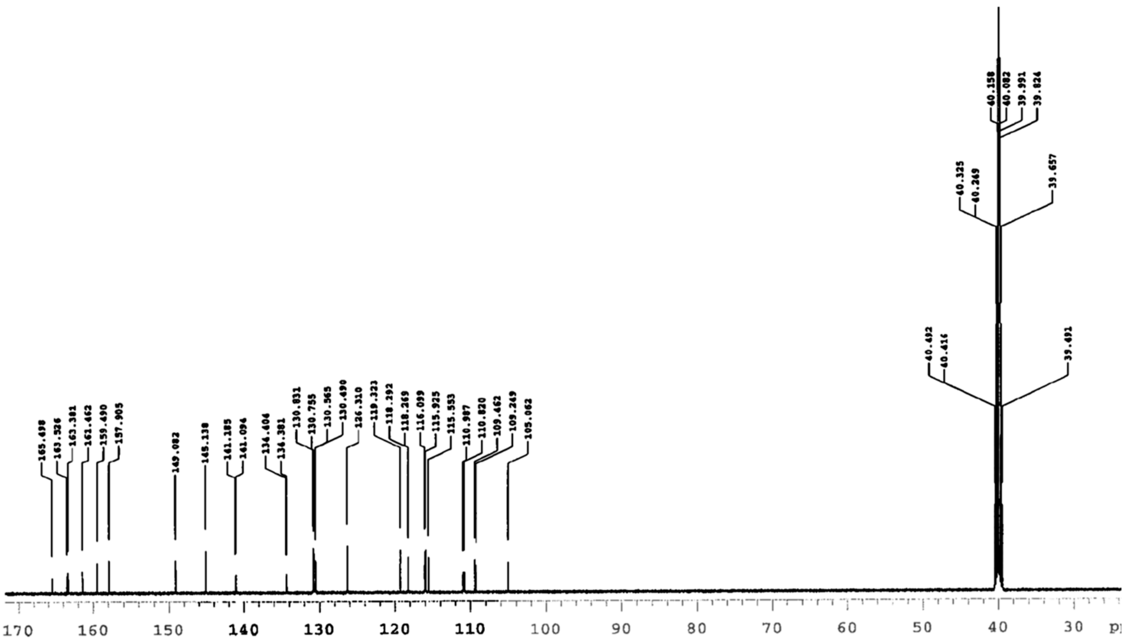

Figure S4. ^{13}C NMR Spectrum of Compound **2b.**

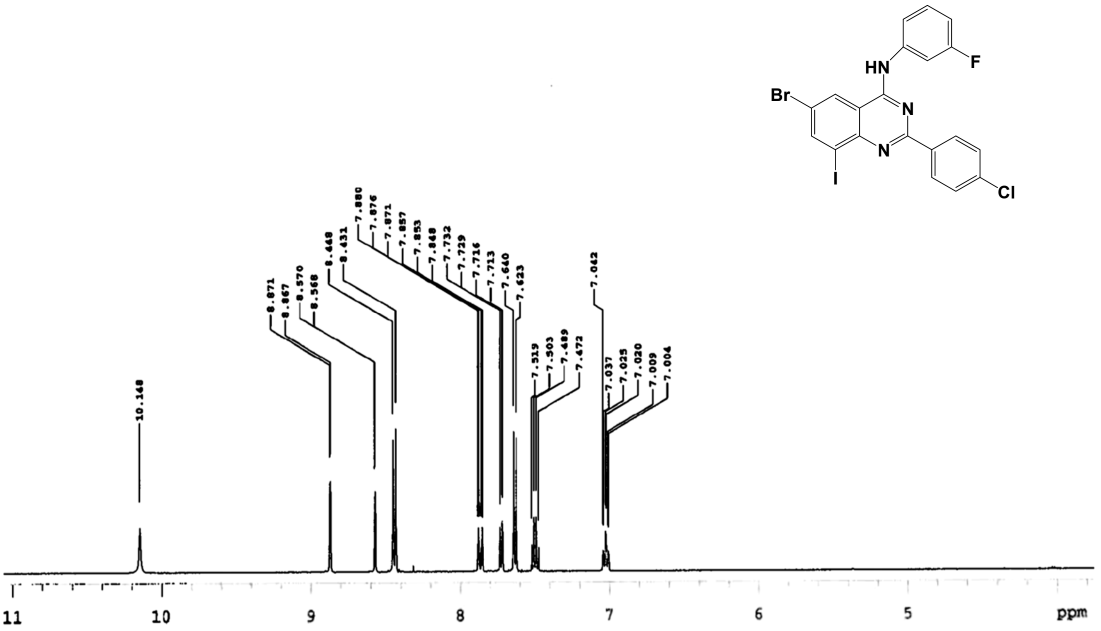

Figure S5. ^{1}H NMR Spectrum of Compound **2c**

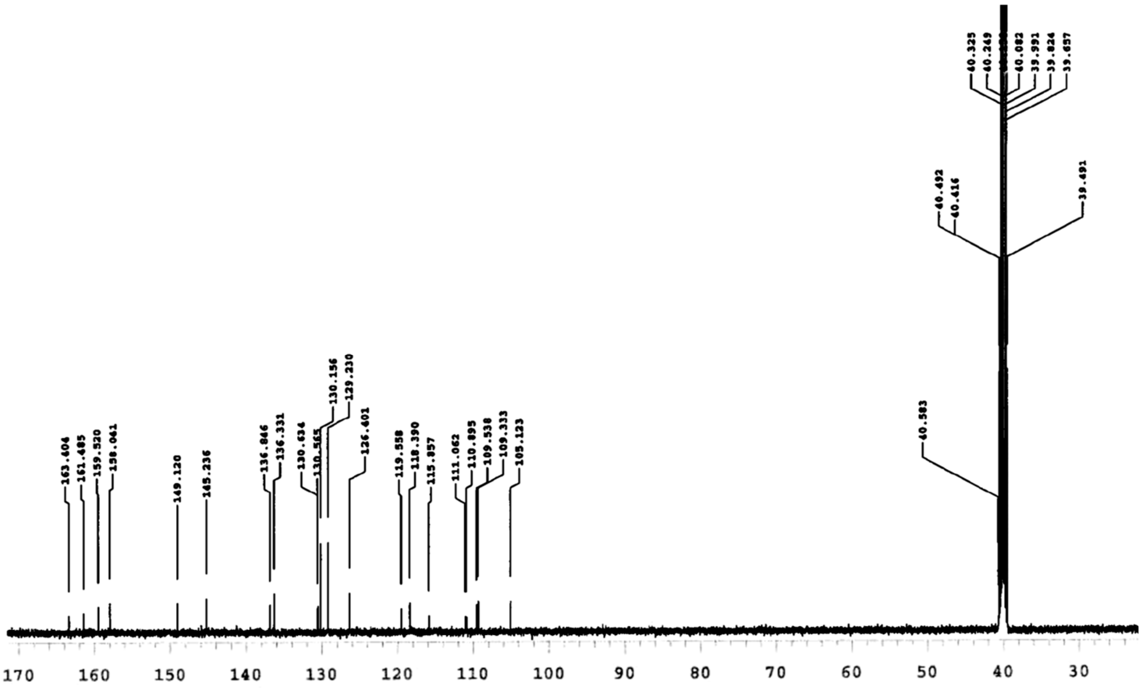

Figure S6. ^{13}C NMR Spectrum of Compound **2c.**

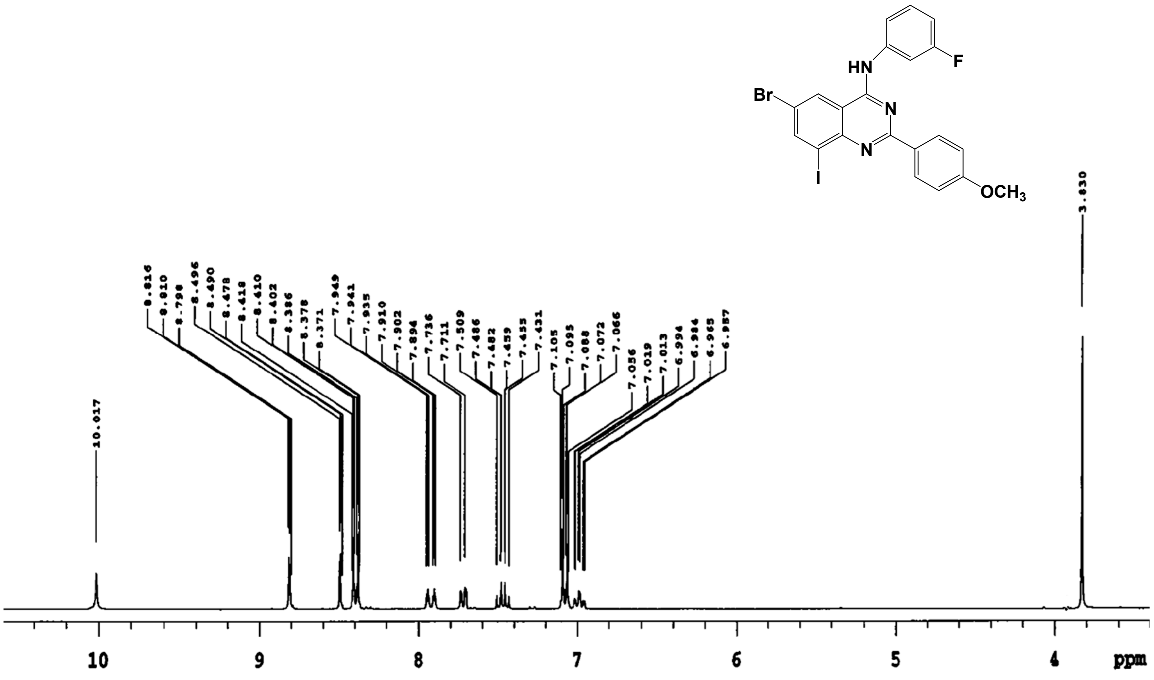

Figure S7. ^{1}H NMR Spectrum of Compound **2d.**

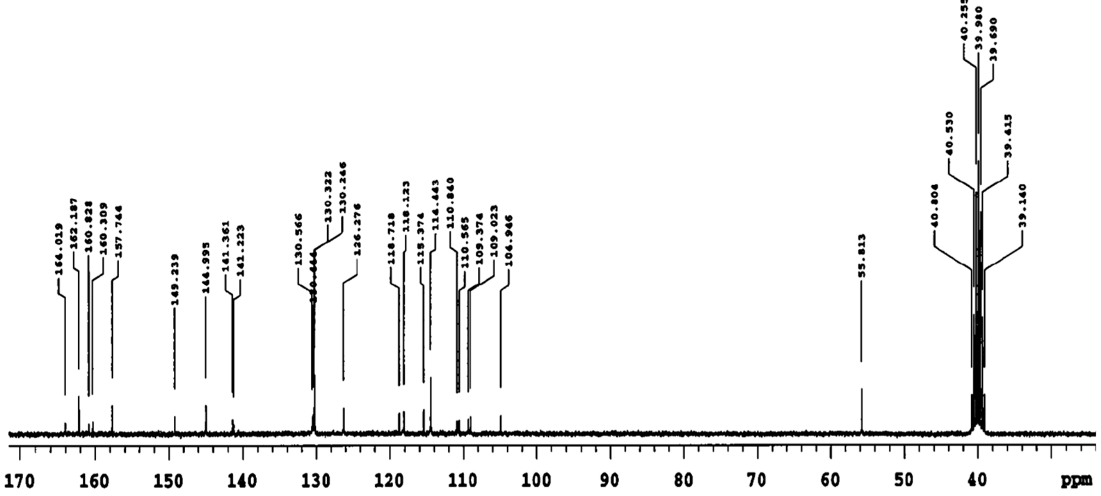

Figure S8. ^{13}C NMR Spectrum of Compound **2d.**

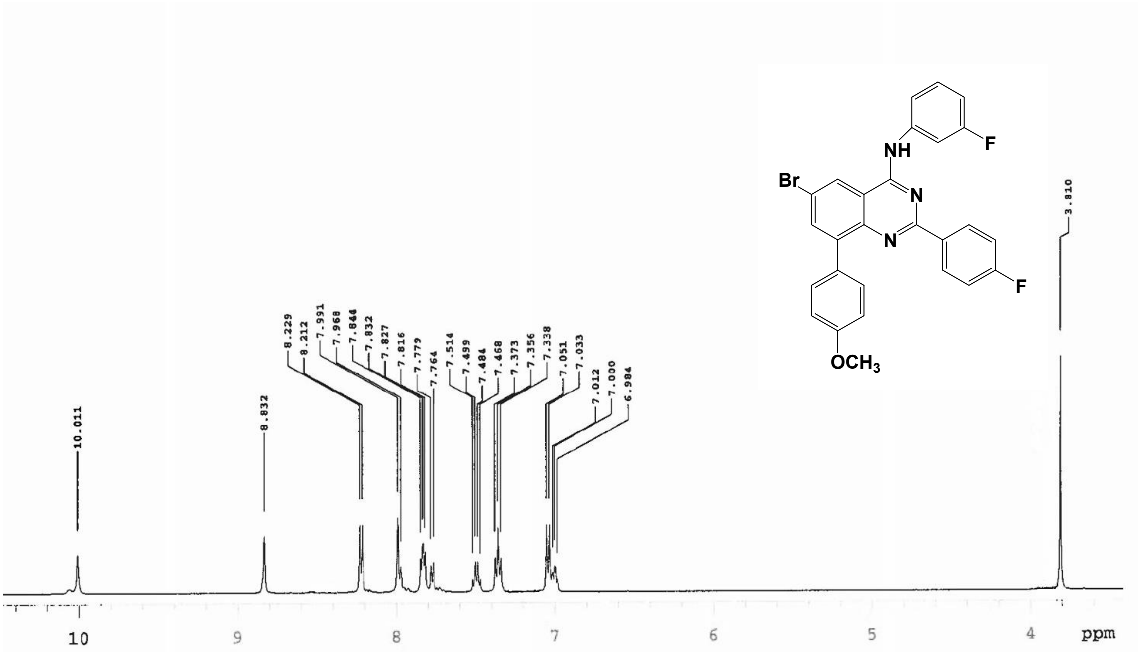

Figure S9. ^{1}H-NMR Spectrum of Compound **3a.**

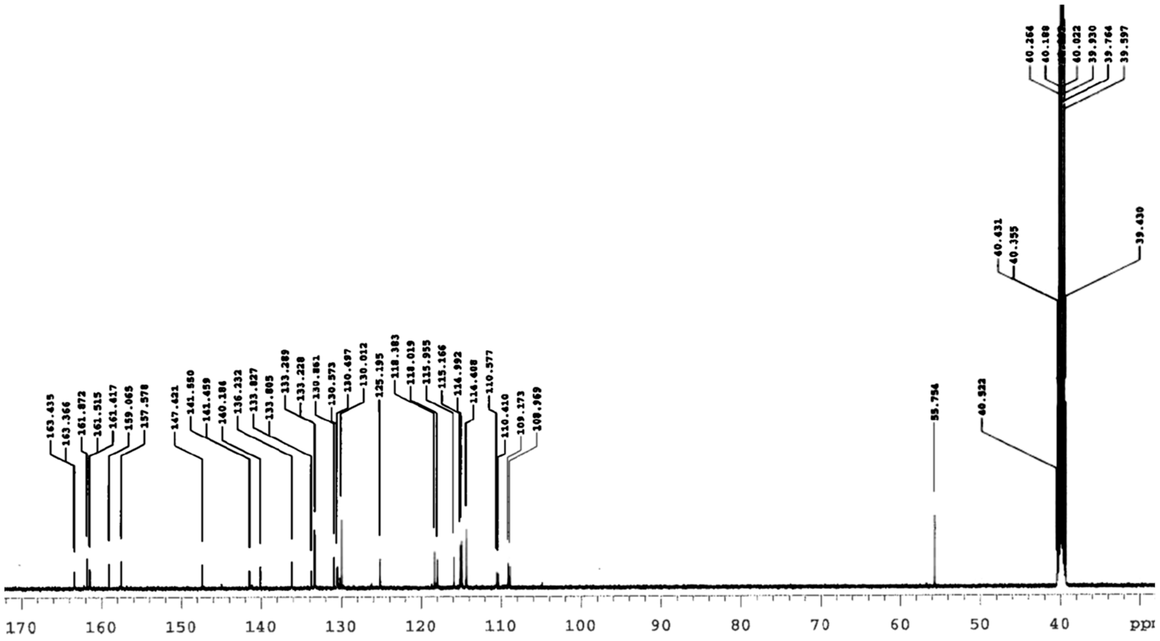

Figure S10. ^{13}C NMR Spectrum of Compound **3b.**

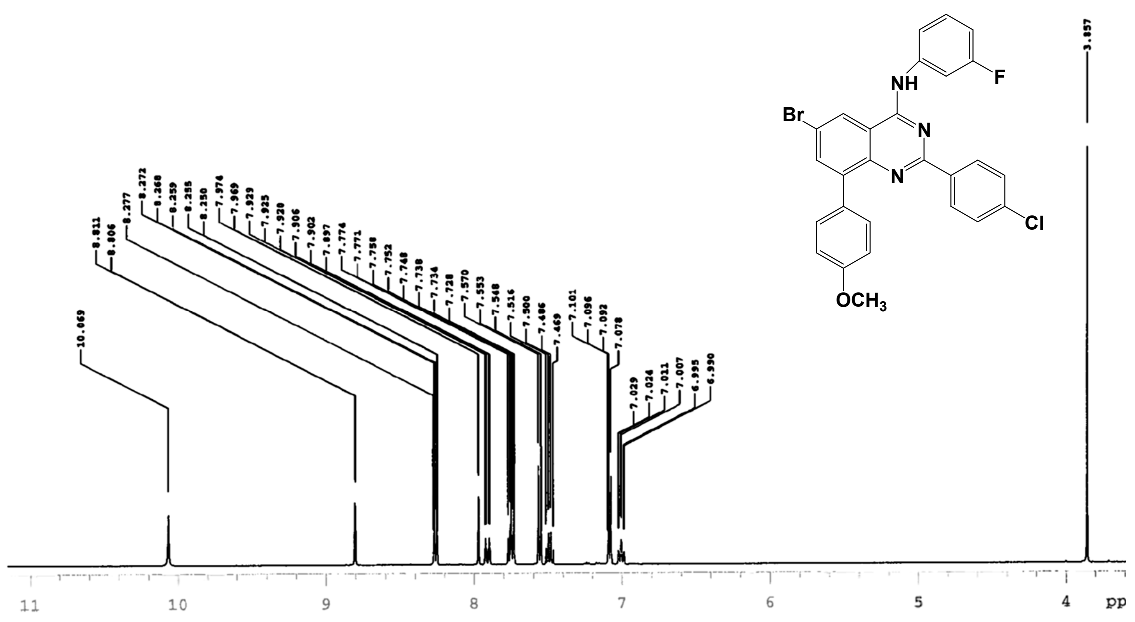

Figure S11. ^{1}H NMR Spectrum of Compound **3c.**

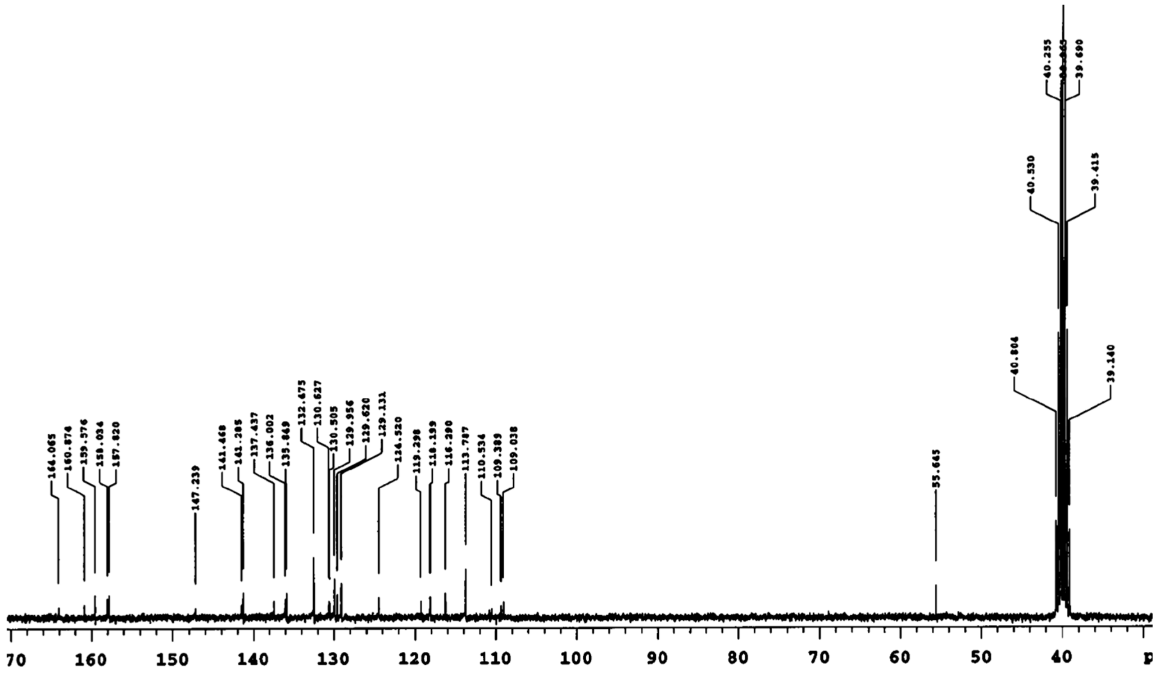

Figure S12. ^{13}C NMR Spectrum of Compound **3c.**

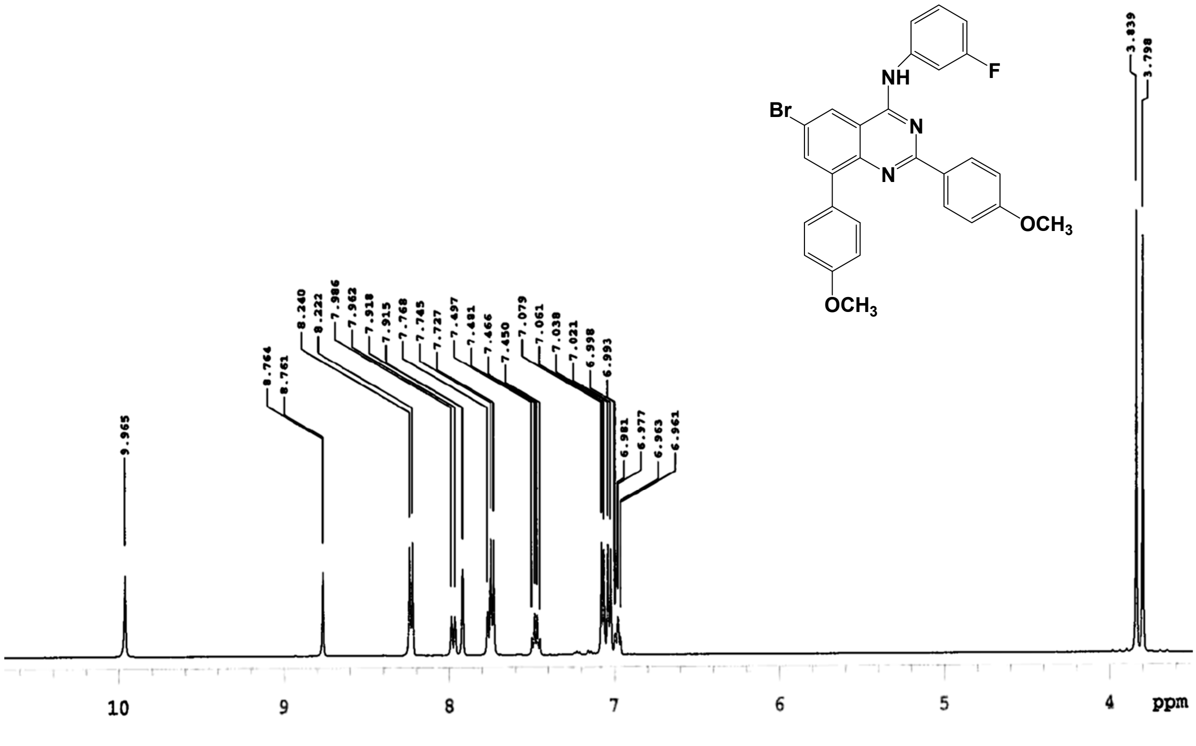

Figure S13. ^{1}H NMR Spectrum of Compound **3d.**

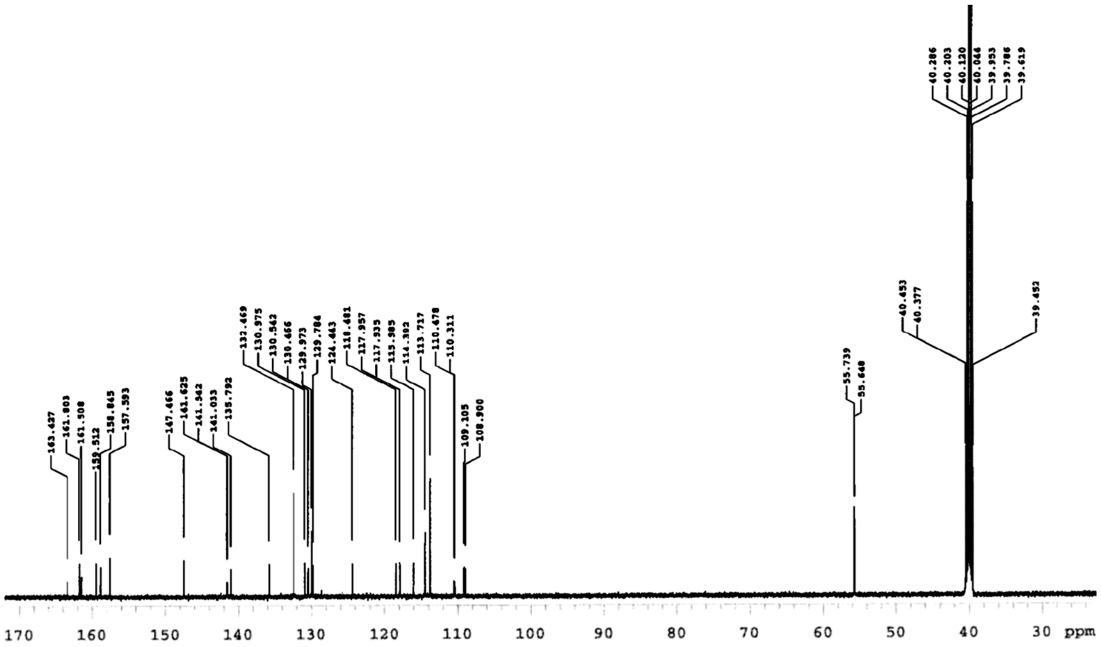

Figure S14. ^{13}C NMR Spectrum of Compound **3d.**

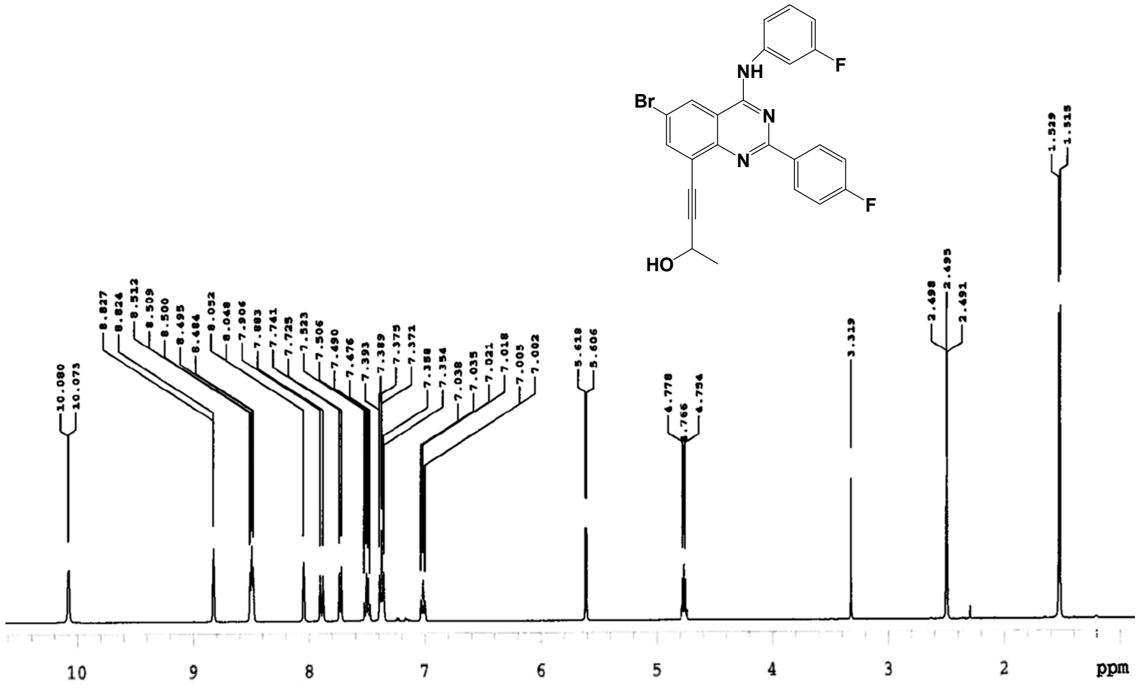

Figure S15. ^{1}H NMR Spectrum of Compound **4b.**

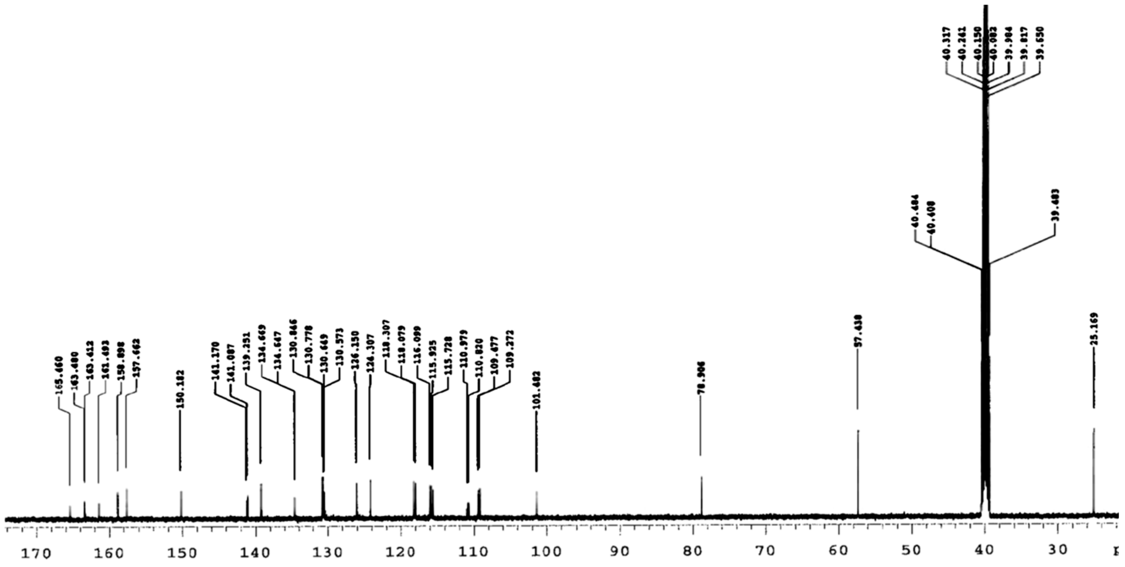

Figure S16. ^{13}C-NMR Spectrum of Compound **4b.**

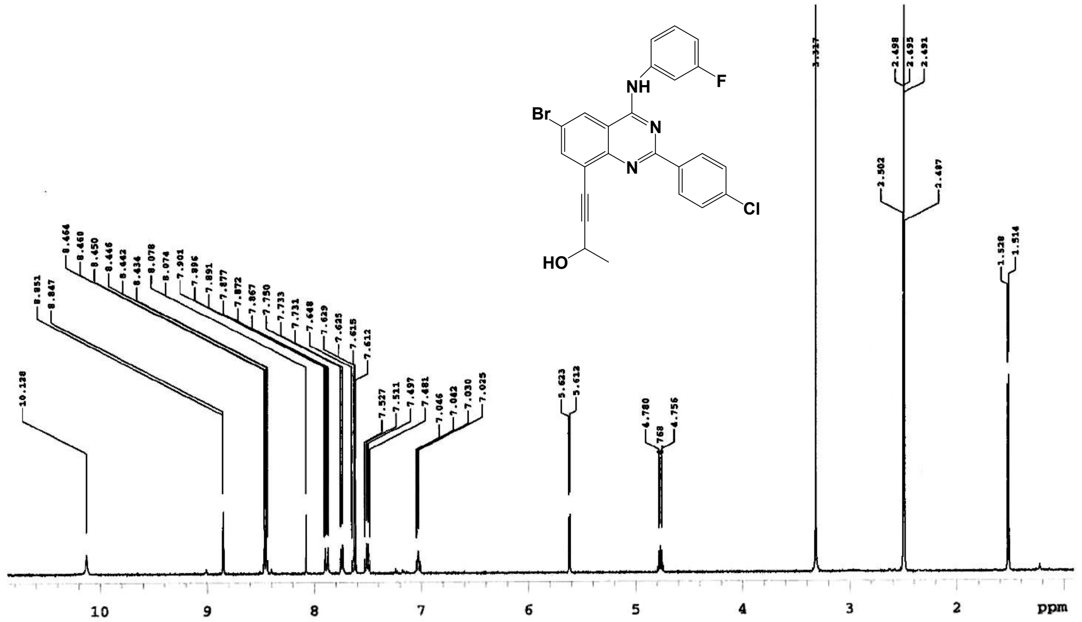

Figure S17. ^{1}H NMR Spectrum of Compound **4c.**

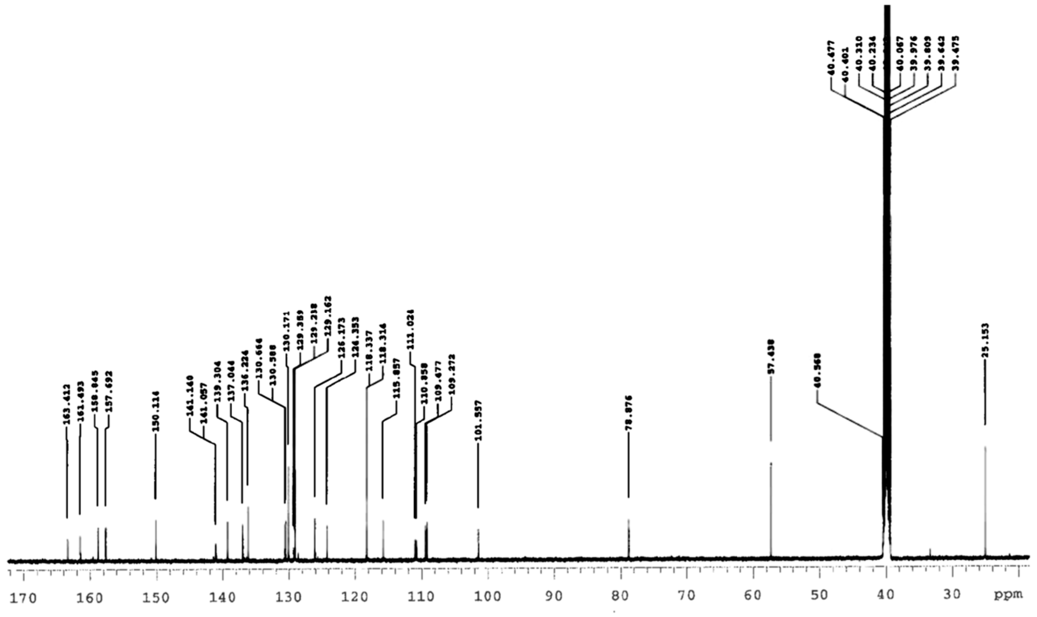

Figure S18. ^{13}C NMR Spectrum of Compound **4c.**

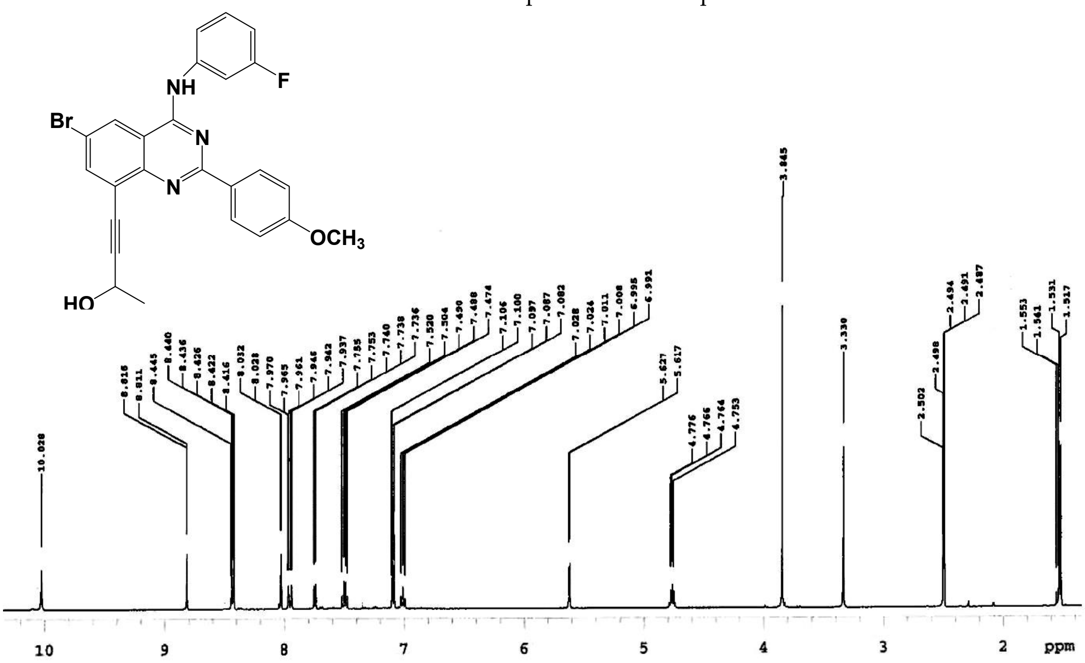

Figure S19. ^{1}H NMR Spectrum of Compound **4d.**

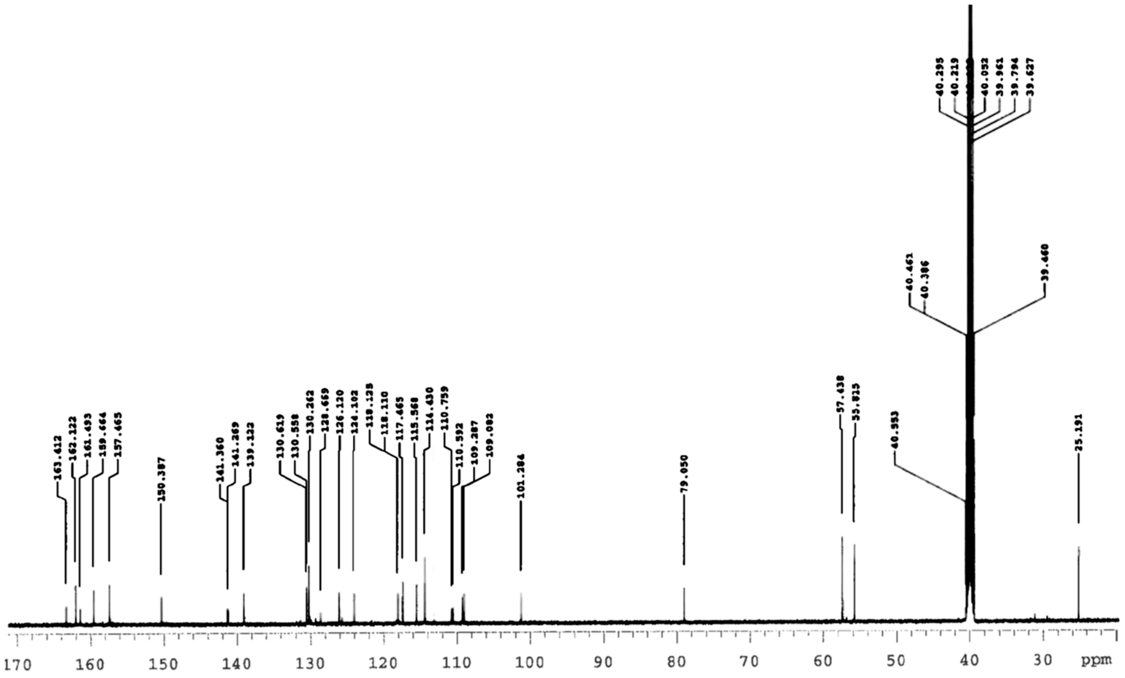

Figure S20. ^{13}C NMR Spectrum of Compound **4d.**

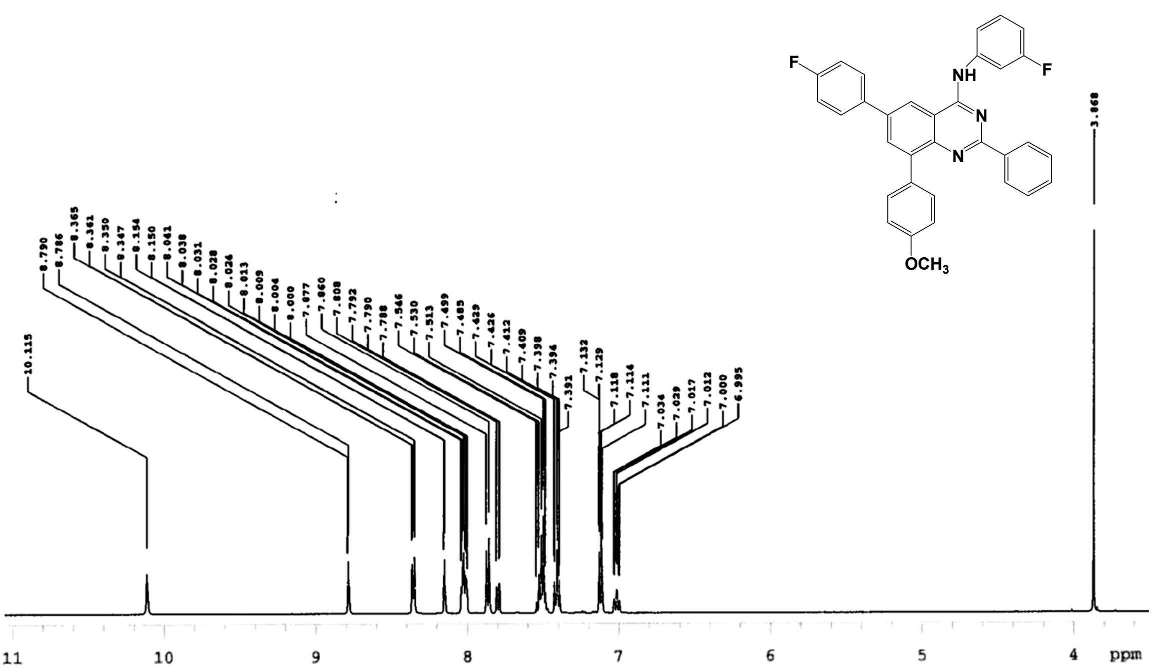

Figure S21. ^{1}H NMR Spectrum of Compound **5a.**

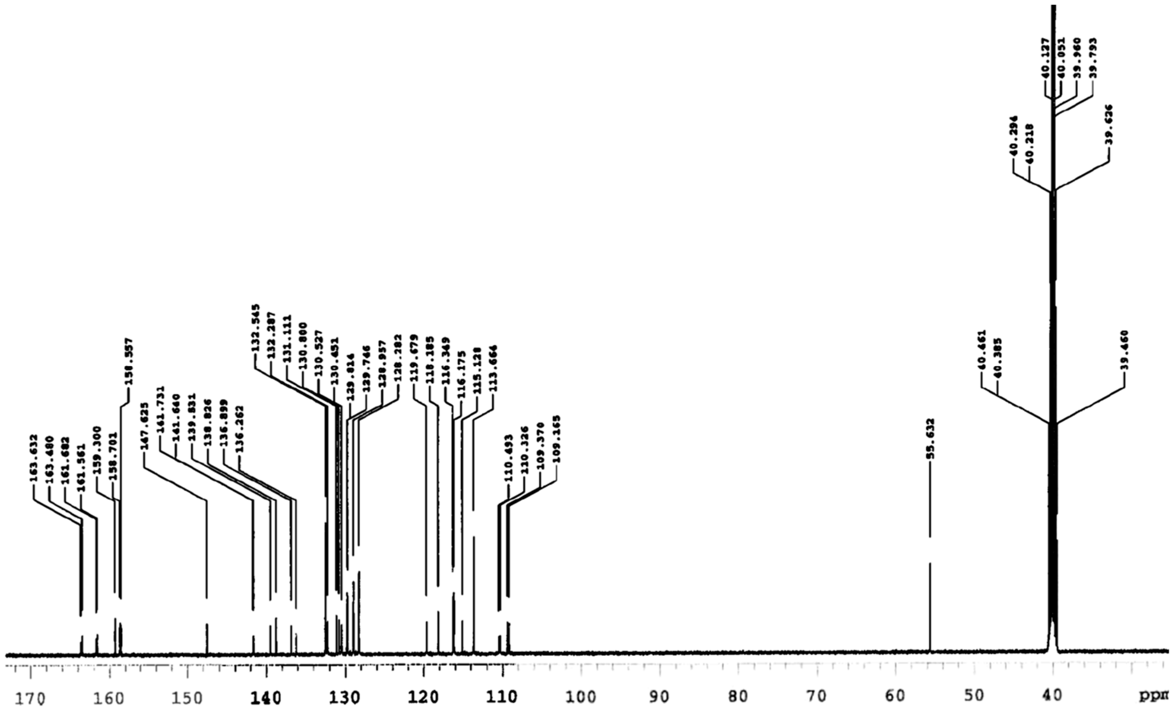

Figure S22. ^{13}C NMR Spectrum of Compound **5a.**

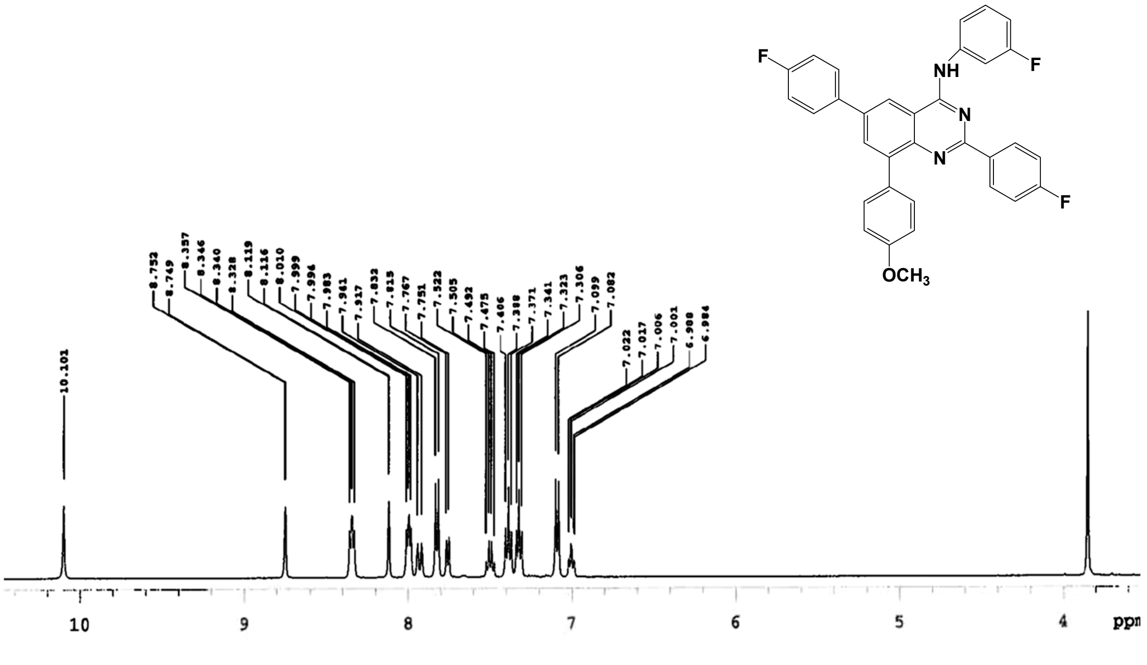

Figure S23. ^{1}H NMR Spectrum of Compound **5b.**

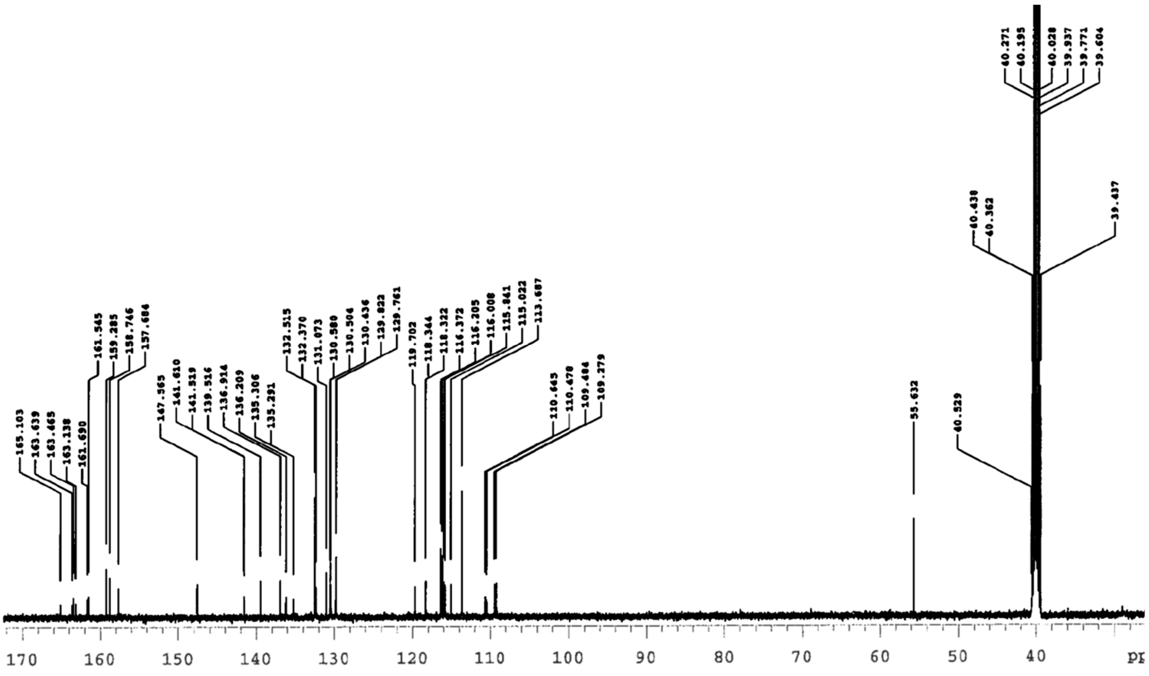

Figure S24. ^{13}C NMR Spectrum of Compound **5b.**

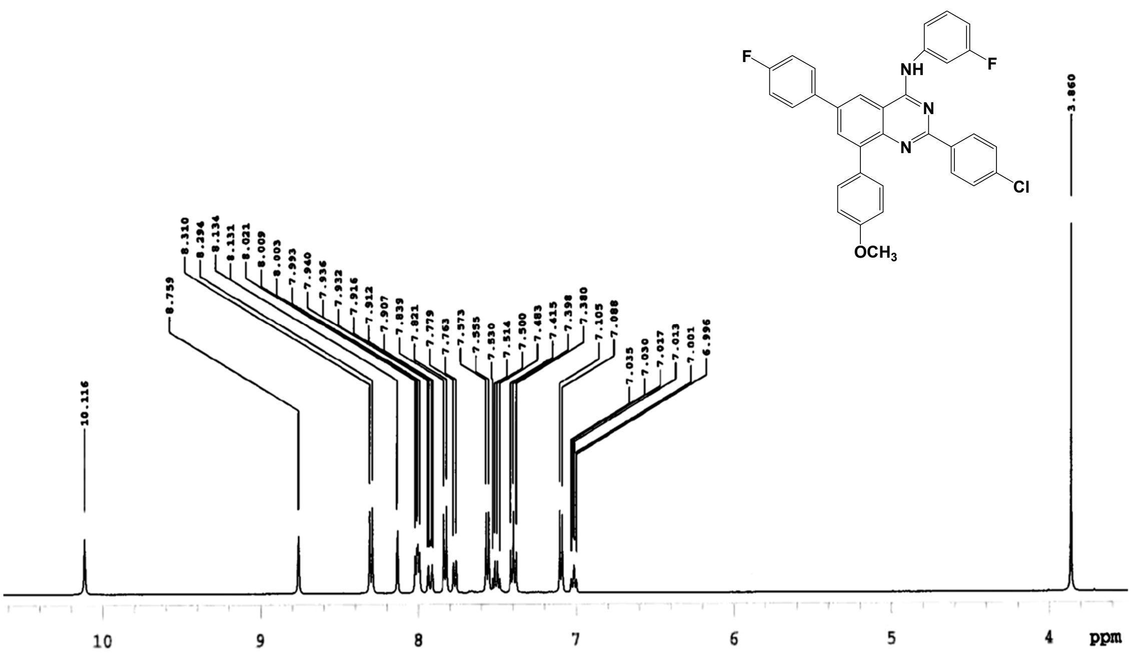

Figure S25. ^{1}H NMR Spectrum of Compound **5c.**

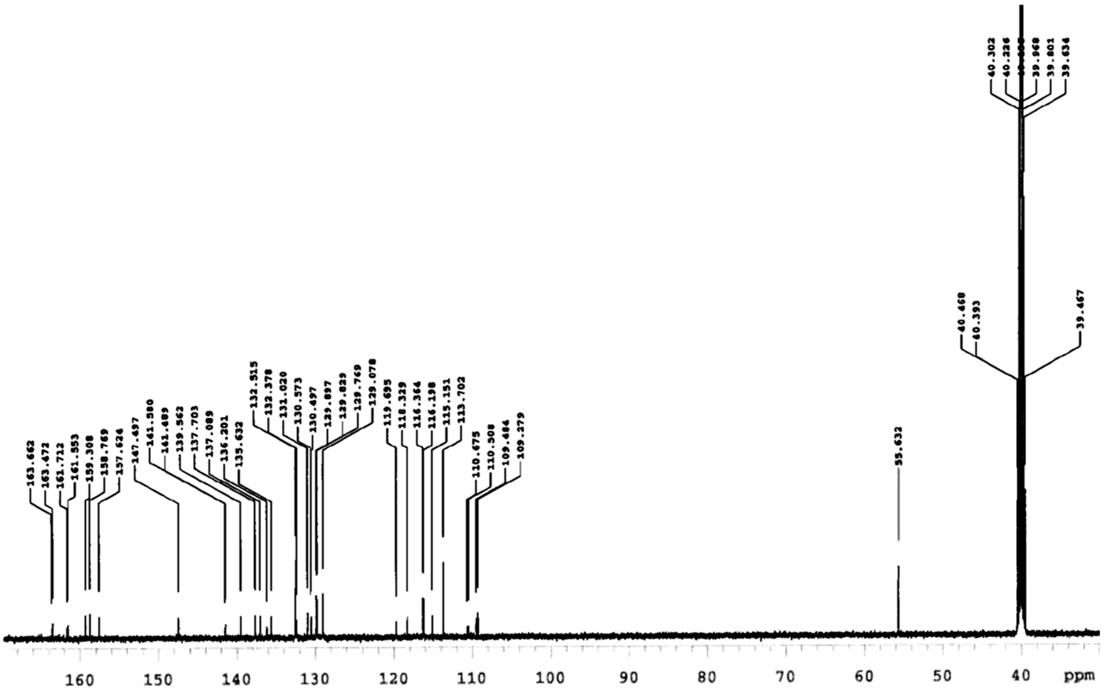

Figure S26. ^{13}C NMR Spectrum of Compound **5c.**

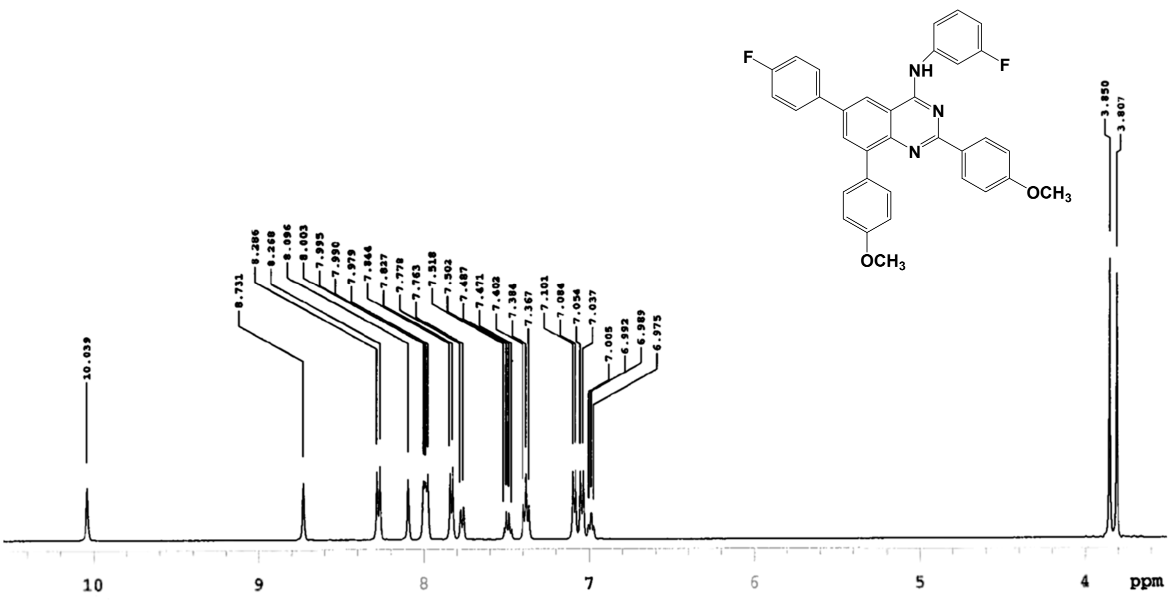

Figure S27. ^{1}H NMR Spectrum of Compound **5d.**

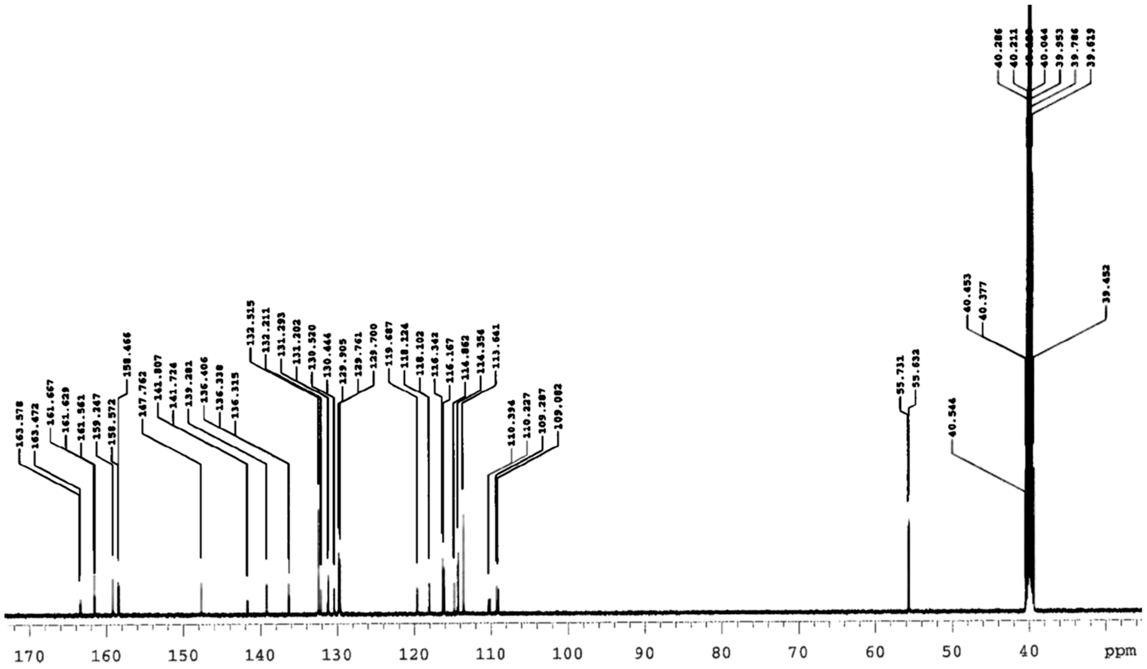

Figure S28. ^{13}C NMR Spectrum of Compound **5d.**

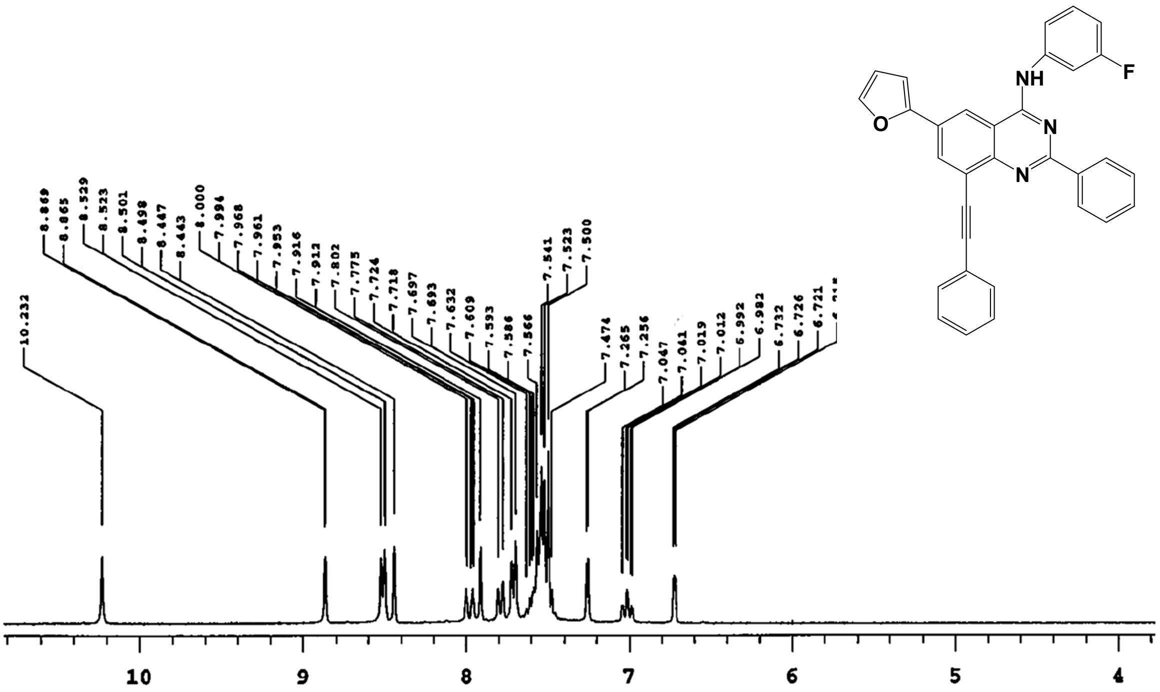

Figure S29. ^{1}H NMR Spectrum of Compound **6a.**

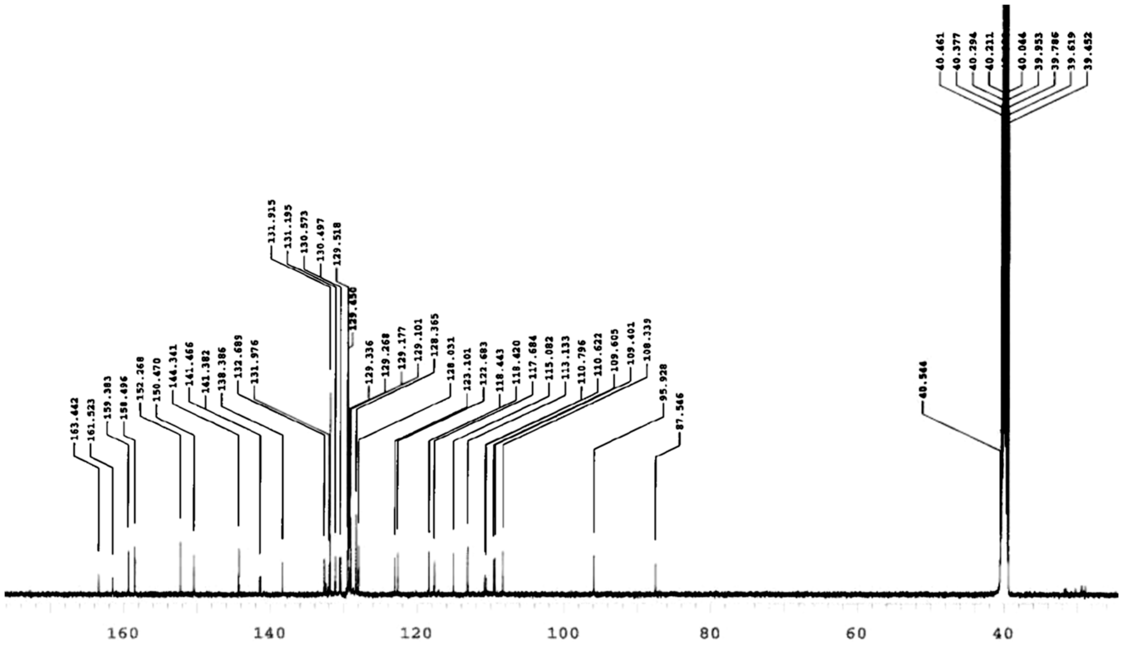

Figure S30. ^{13}C NMR Spectrum of Compound **6a.**

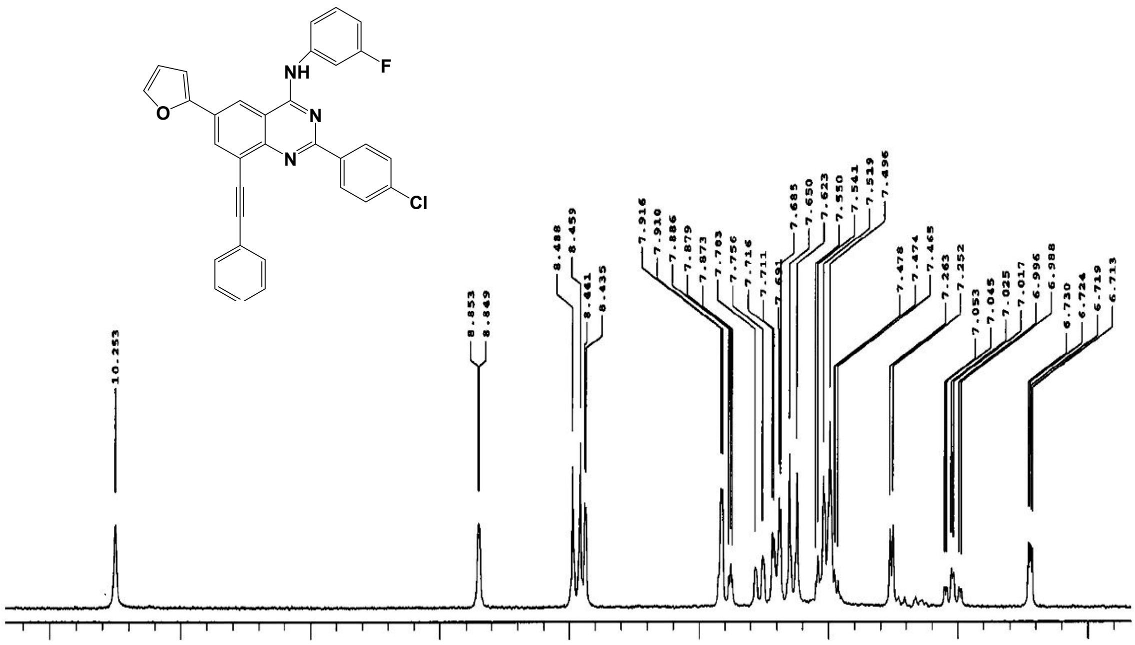

Figure S31. ^{1}H NMR Spectrum of Compound **6c.**

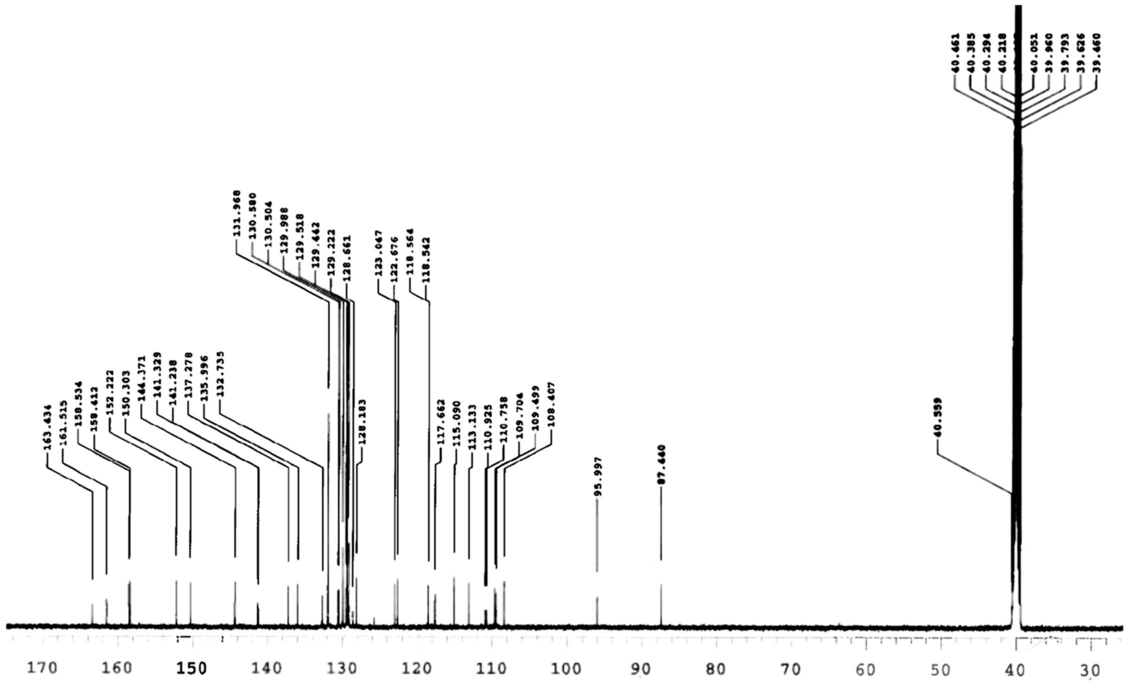

Figure S32. ^{13}C NMR Spectrum of Compound **6c.**

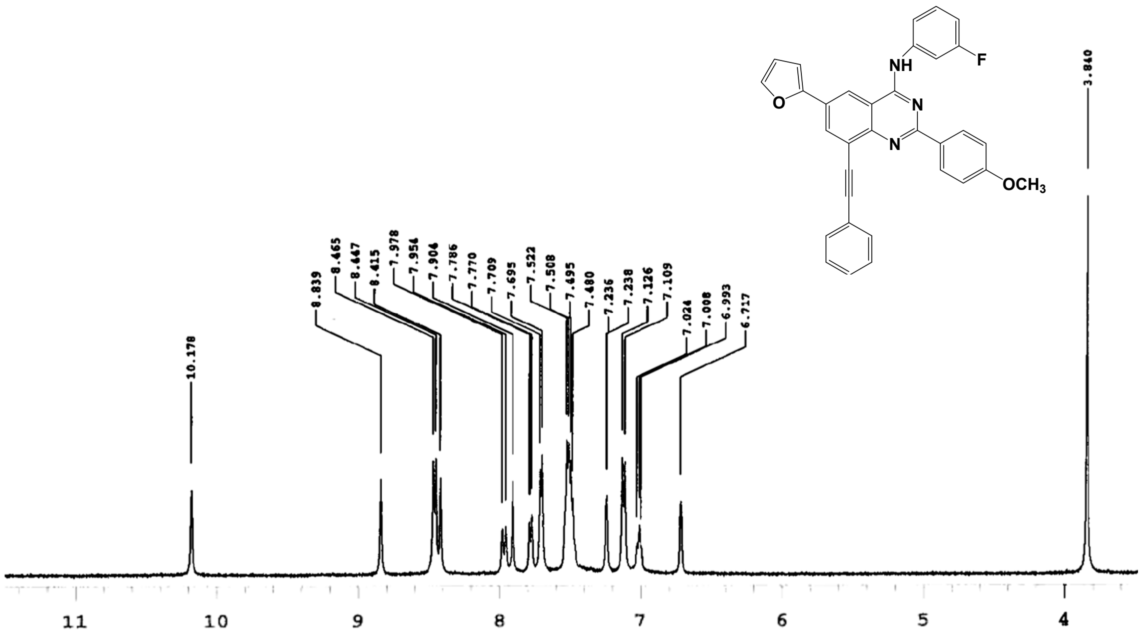

Figure S33. ^{1}H NMR Spectrum of Compound **6d.**

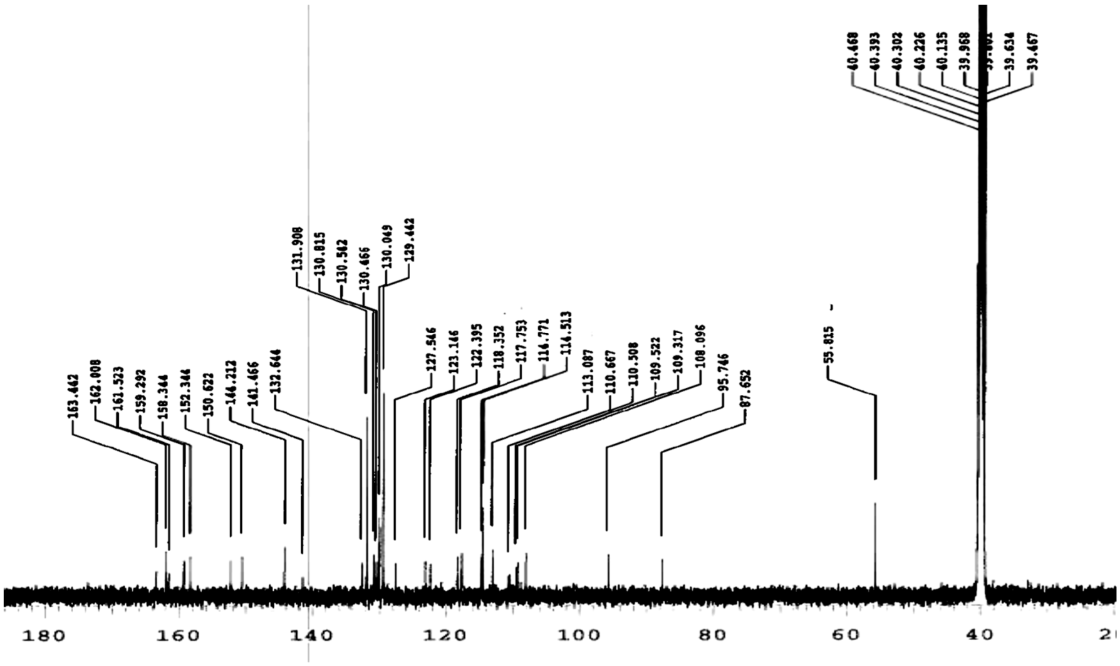

Figure S34. ^{13}C NMR Spectrum of Compound **6d.**

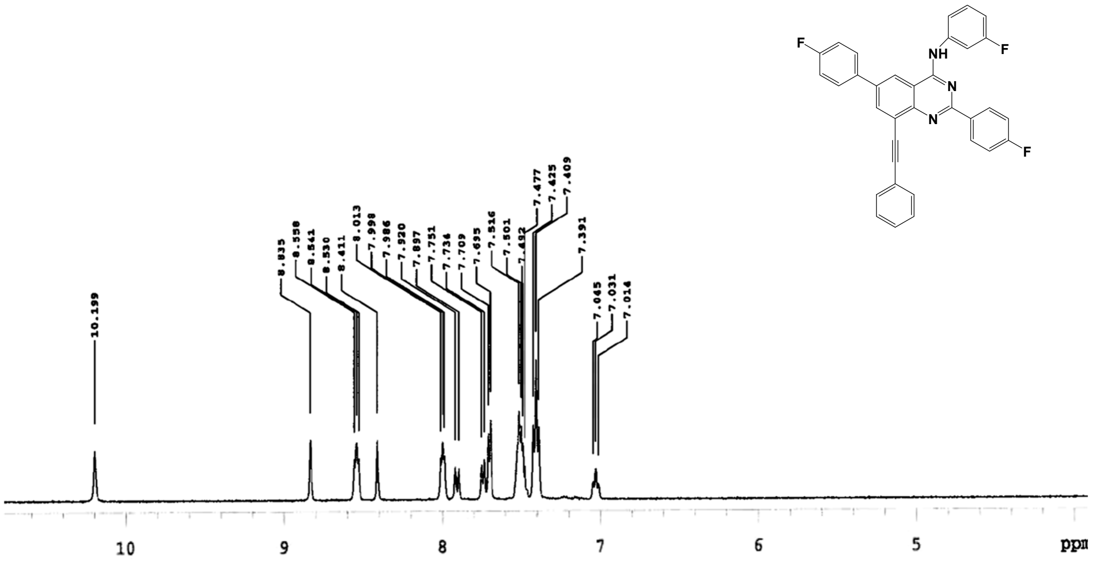

Figure S35. ^{1}H NMR Spectrum of Compound **7a**.

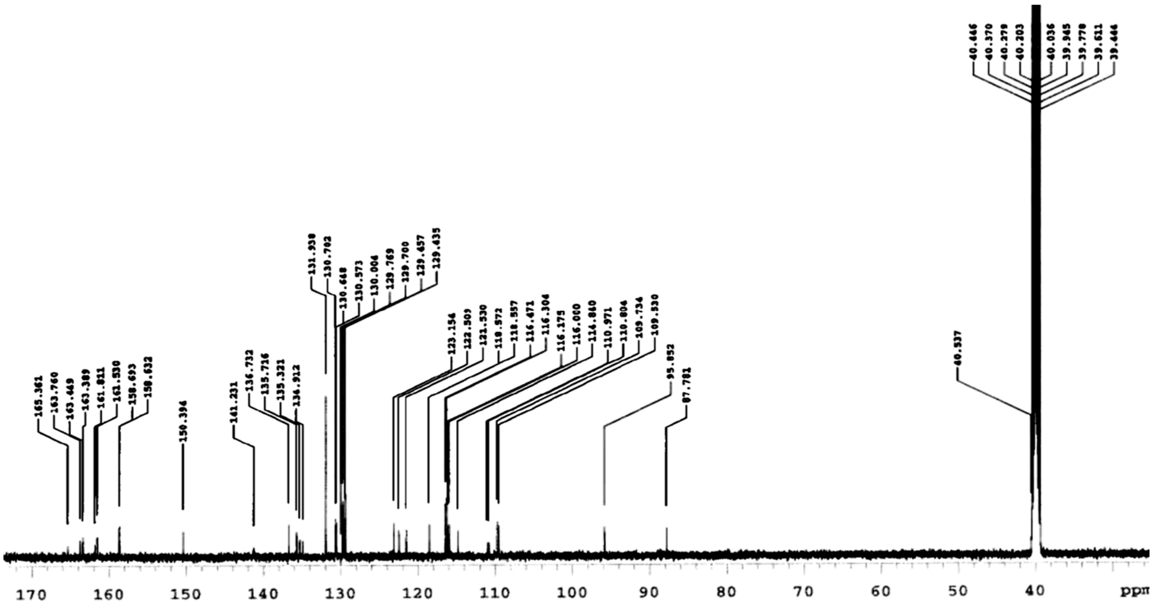

Figure S36. ^{13}C NMR Spectrum of Compound **7a.**

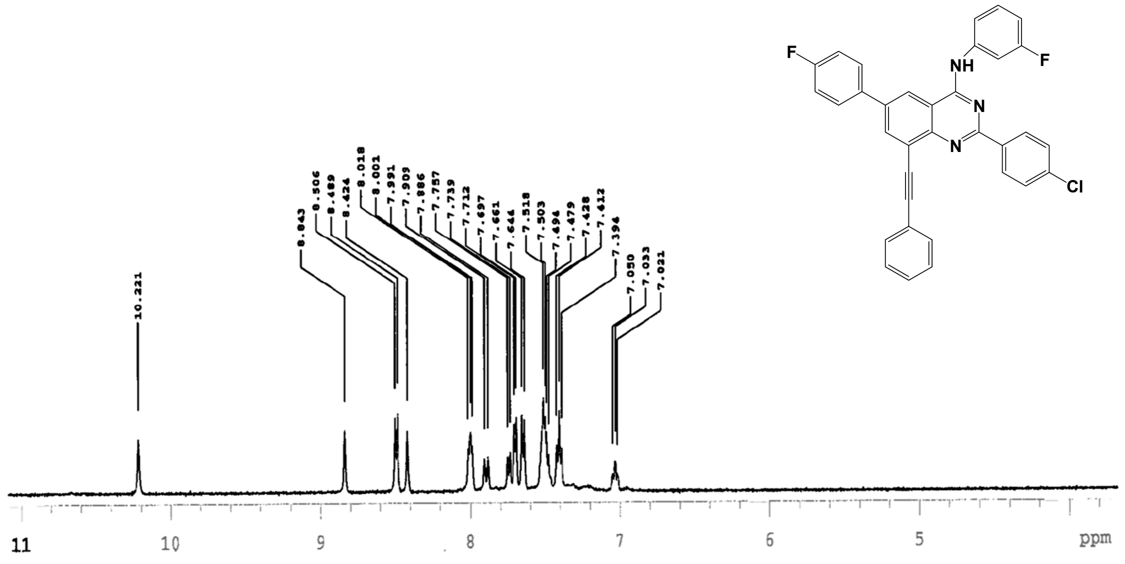

Figure S37. ^{1}H NMR Spectrum of Compound **7b.**

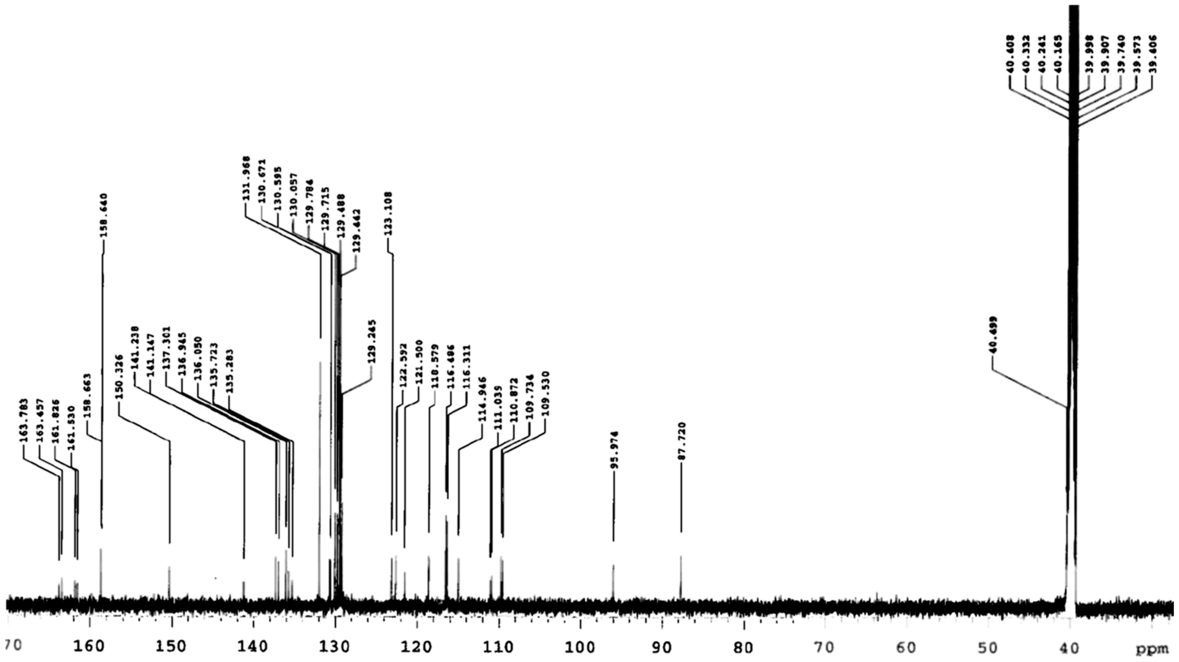

Figure S38. ^{13}C NMR Spectrum of Compound **7b.**

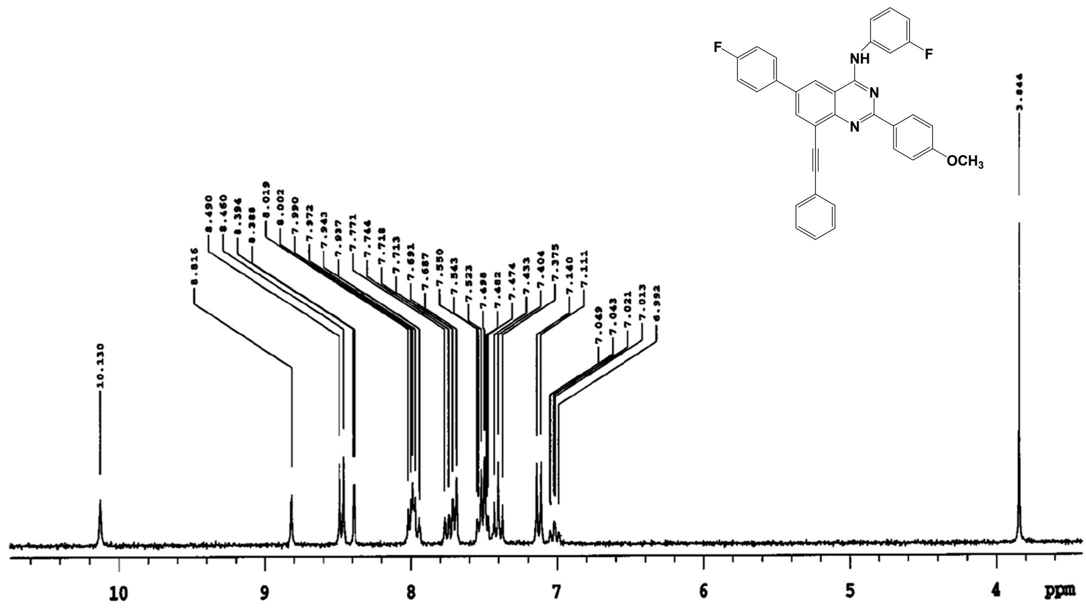

Figure S39. ^{1}H NMR Spectrum of Compound **7c.**

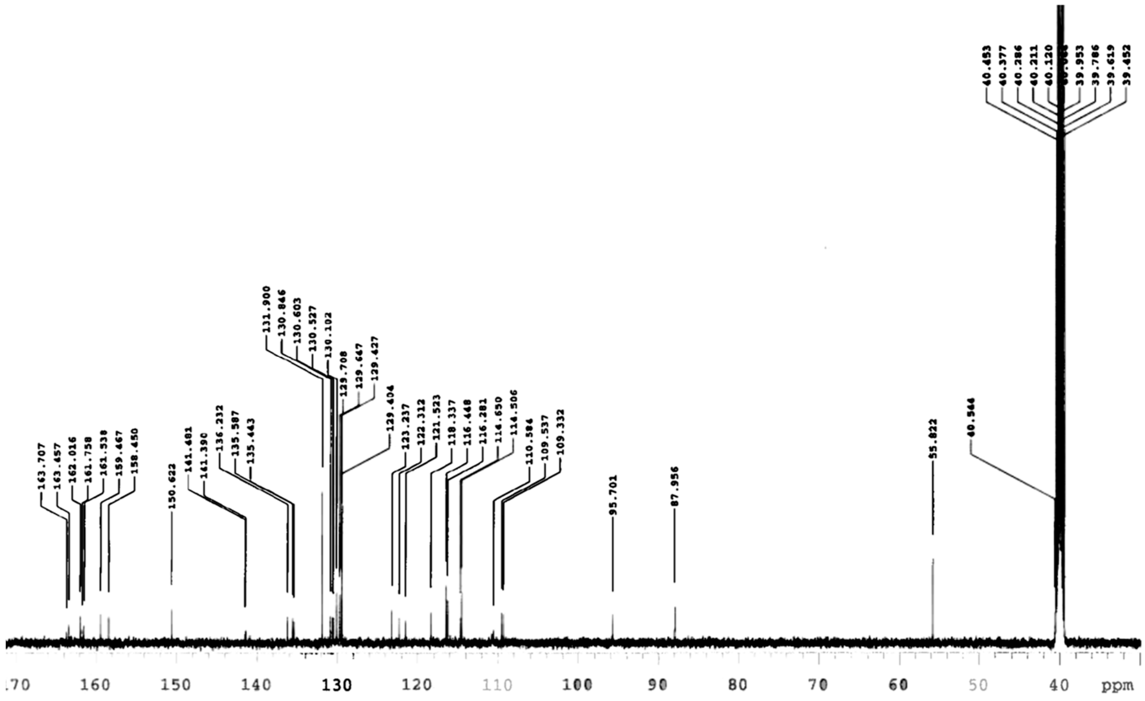

Figure S40. ^{13}C NMR Spectrum of Compounds **7**.

3. % Cell Viability and LC_{50} Values of Doxorubicin Hydrochloride and Compounds 2–7

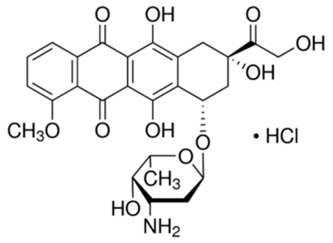

Figure S41. Chemical structure of Doxorubicin hydrochloride.

Table S1. Percentage cell viability of MCF-7, HeLa and A549 cells exposed to different concentrations of doxorubicin hydrochloride.

Concentration (µM)	Log Concentration	%Viability MCF-7	SD	%Viability HeLa	SD	%Viability A549	SD
20	1.30103	27.68	1.09	5.59	0.71	36.24	1.29
10	1	32.10	1.06	8.77	0.10	41.89	0.94
5	0.69897	34.25	1.64	16.83	0.20	45.91	0.68
2	0.30103	35.55	1.25	47.65	1.10	50.88	1.25
1	0	57.5	1.57	55.64	0.73	59.29	1.21
0.5	−0.30103	70.47	1.74	67.36	1.90	68.95	1.76

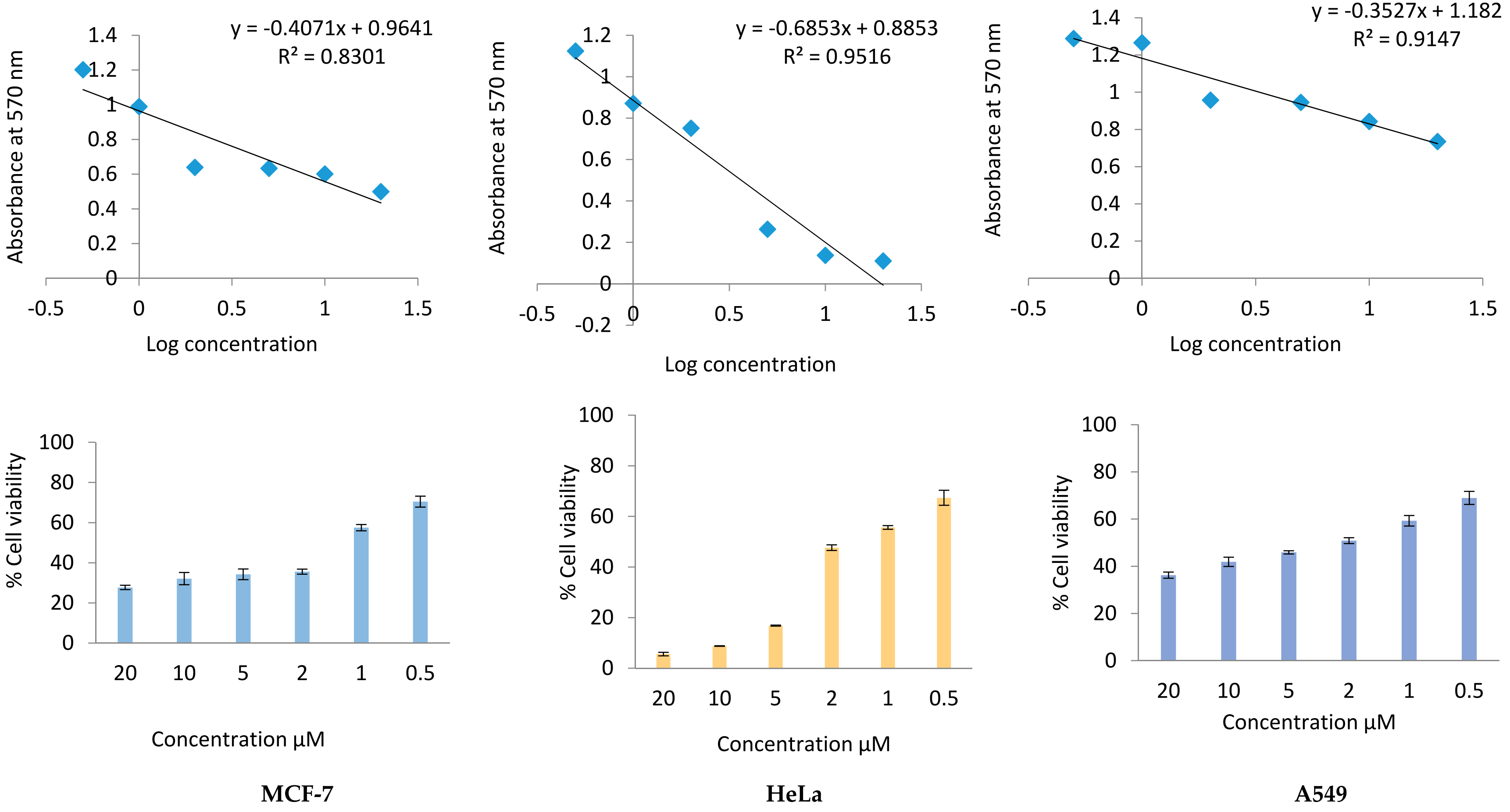

Figure S42. Linear regression plots and percentage cell viability graphs of MCF-7, HeLa and A549 cells exposed to different concentrations of doxorubicin hydrochloride.

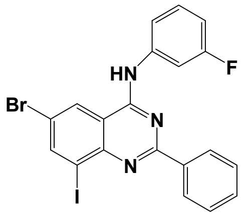

2a

Figure S43. Chemical structure of **2a**

Table S2. Percentage cell viability of MCF-7, HeLa and A549cells exposed to different concentrations of **2a**.

Concentration (µg/mL)	Log Concentration	%Viability MCF-7	SD	%Viability HeLa	SD	%Viability A549	SD
100	2	3.68	0.85	2.16	0.10	2.09	0.48
10	1	65.01	2.93	71.90	1.73	67.16	1.39
1	0	74.05	0.10	89.10	1.64	81.69	0.75
0.1	−1	91.20	2.08	96.52	1.27	89.91	2.40

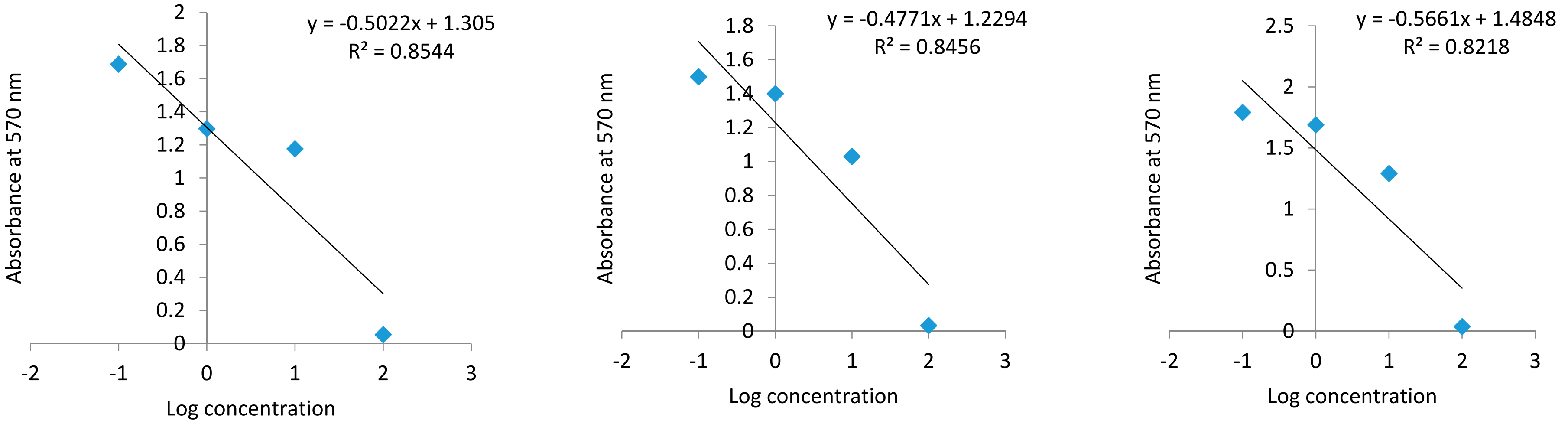

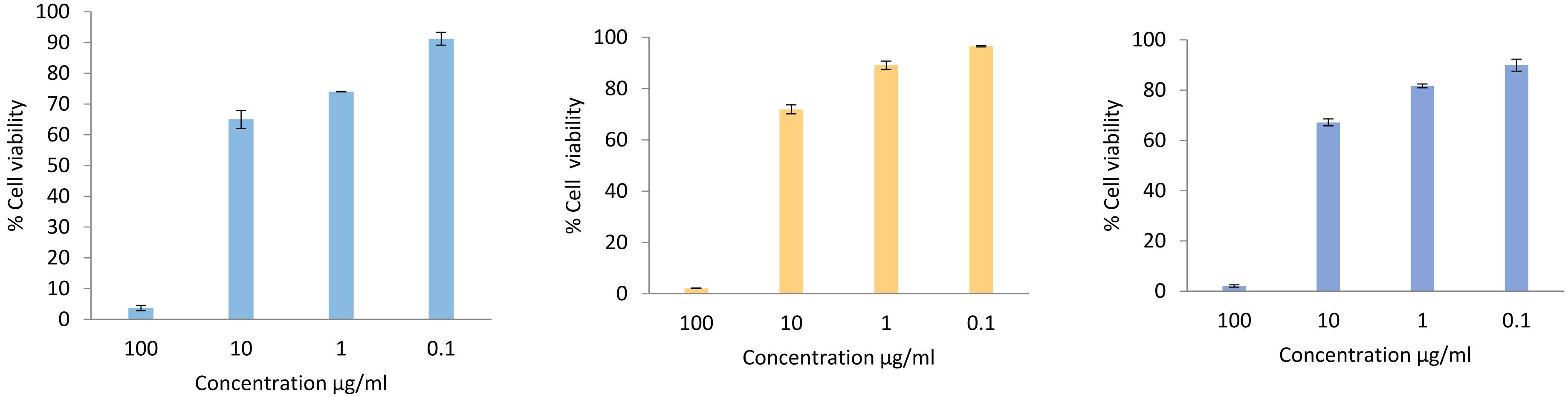

MCF-7 **HeLa** **A549**

Figure S44. Linear regression plots and percentage cell viability graphs of MCF-7, HeLa and A549 cells exposed to different concentrations of **2a.**

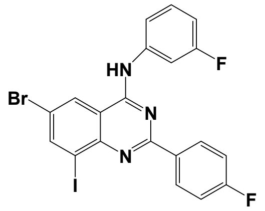

2b

Figure S45. Chemical structure of **2b.**

Table S3. Percentage cell viability of MCF-7, HeLa and A549 cells exposed to different concentrations of **2b**.

Concentration (µg/mL)	Log Concentration	%Viability MCF-7	SD	%Viability HeLa	SD	%Viability A549	SD
100	2	4.96	0.40	4.77	0.27	4.85	1.70
10	1	45.47	2.38	62.38	1.87	66.36	1.39
1	0	52.25	1.25	70.47	2.37	74.53	1.15
0.1	−1	73.55	0.61	76.85	1.46	79.10	2.00

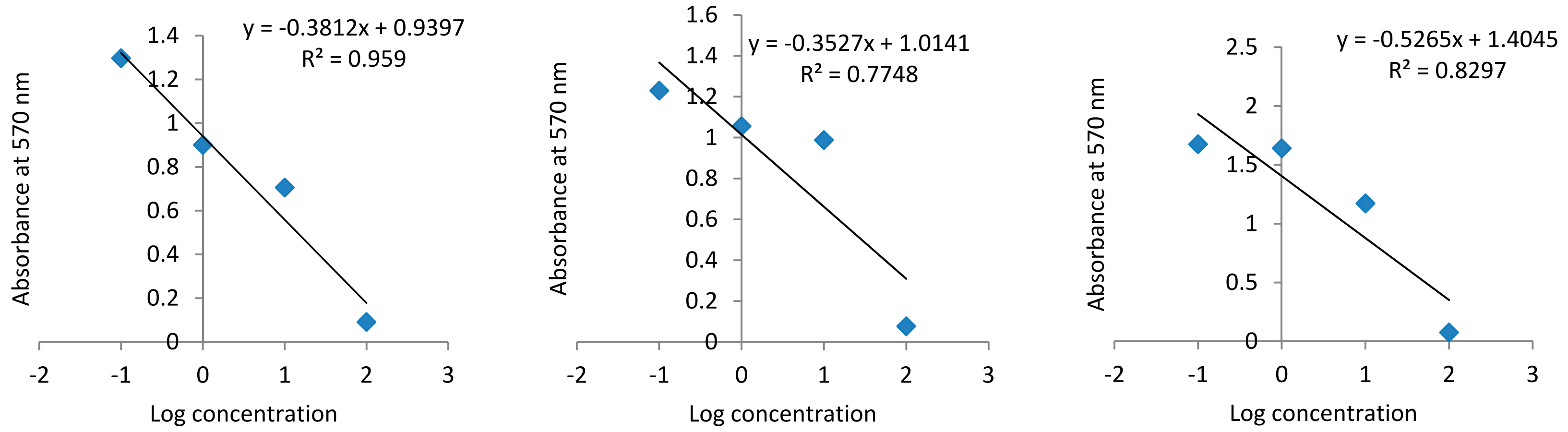

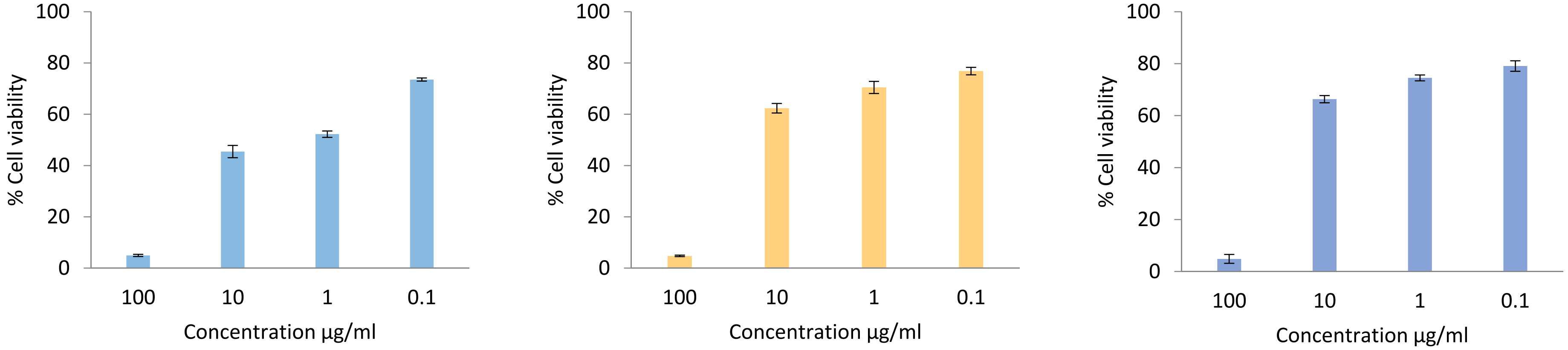

MCF-7 **HeLa** **A549**

Figure S46. Linear regression plots and percentage cell viability graphs of MCF-7, HeLa and A549 cells exposed to different concentrations of **2b.**

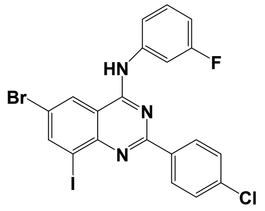

2c

Figure S47. Chemical structure of **2c.**

Table S4. Percentage cell viability of MCF-7, HeLa and A549 cells exposed to different concentrations of **2c.**

Concentration (µg/mL)	Log Concentration	%Viability MCF-7	SD	%Viability HeLa	SD	%Viability A549	SD
100	2	8.54	3.24	11.25	0.73	10.17	1.41
10	1	42.66	1.01	54.58	0.87	63.99	0.74
1	0	50.78	2.92	79.11	0.93	71.21	1.72
0.1	-1	74.49	2.78	97.68	1.87	85.23	1.40

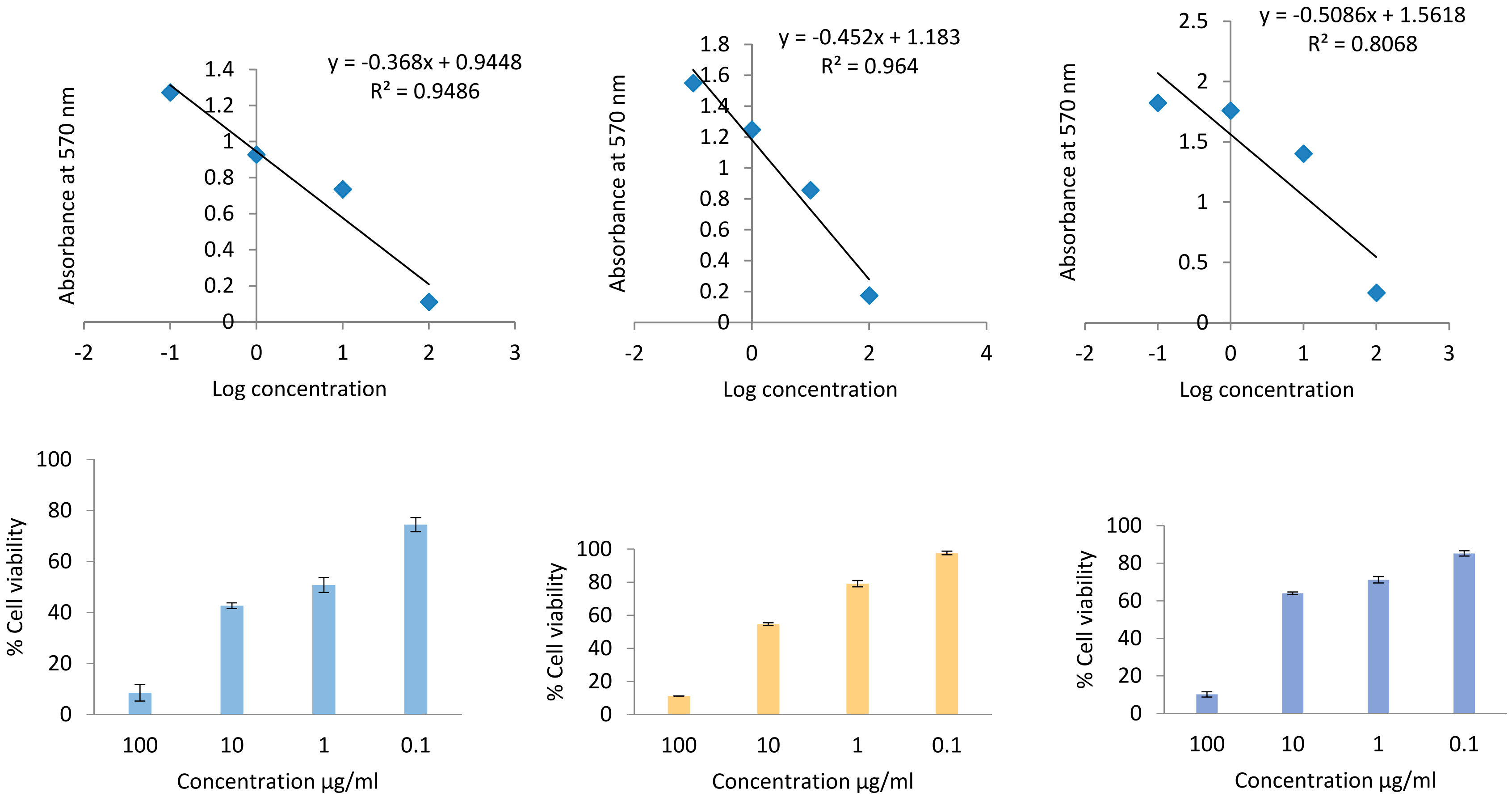

MCF-7 **HeLa** **A549**

Figure S48. Linear regression plots and percentage cell viability graphs of MCF-7, HeLa and A549 cells exposed to different concentrations of **2c**.

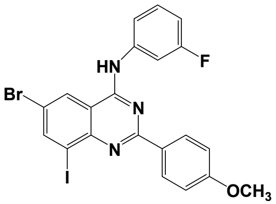

2d

Figure S49. Chemical structure of **2d**.

Table S5. Percentage cell viability of MCF-7, HeLa and A549 cells exposed to different concentrations of **2d**.

Concentration (µg/mL)	Log Concentration	%Viability MCF-7	SD	%Viability HeLa	SD	%Viability A549	SD
100	2	38.30	1.20	34.27	1.06	35.91	0.95
10	1	79.28	2.05	49.61	1.611	73.76	1.23
1	0	86.69	1.55	90.55	2.15	93.49	1.74
0.1	−1	91.38	0.42	95.13	2.47	96.95	0.61

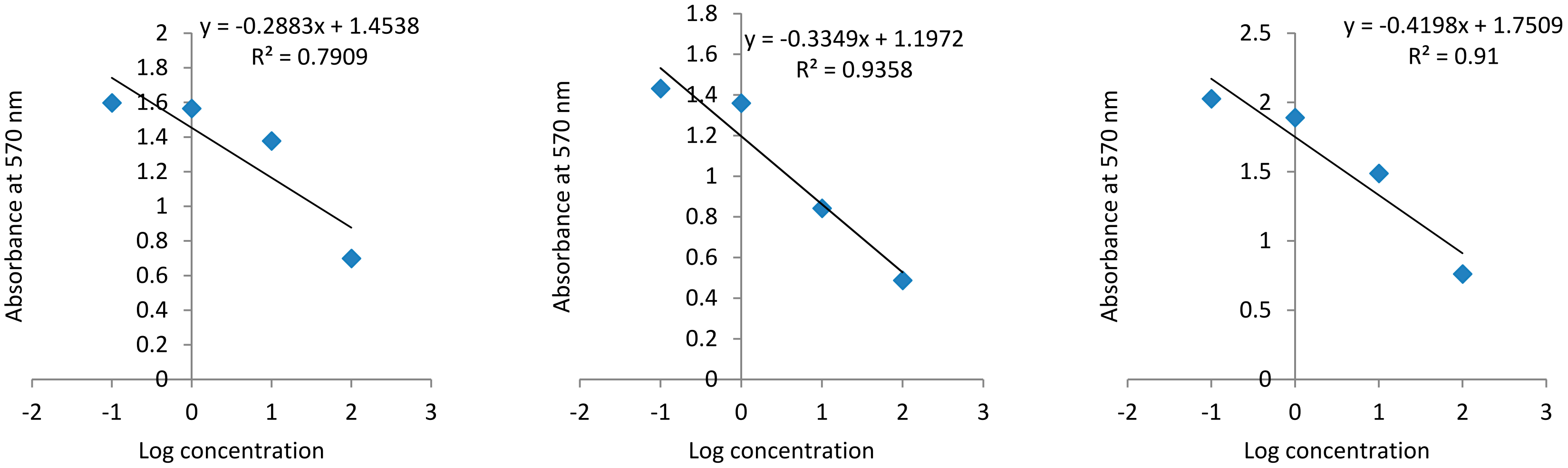

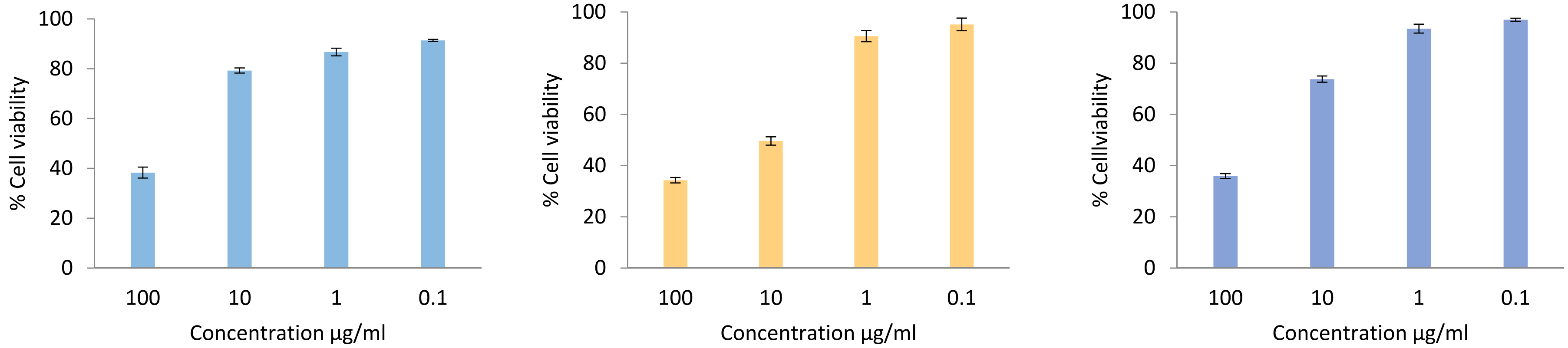

MCF-7 **HeLa** **A549**

Figure S50. Linear regression plots and percentage cell viability graphs of MCF-7, HeLa and A549 cells exposed to different concentrations of **2d.**

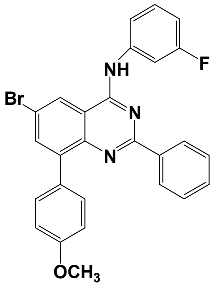

3a

Figure S51. Chemical structure of **3a.**

Table S6. Percentage cell viability of MCF-7, HeLa and A549 cells exposed to different concentrations of **3a.**

Concentration (µg/mL)	Log Concentration	%Viability MCF-7	SD	%Viability HeLa	SD	%Viability A549	SD
100	2	54.50	0.64	61.28	1.15	54.00	1.55
10	1	69.06	1.82	75.11	0.93	64.41	1.78
1	0	77.24	1.67	93.49	1.56	96.38	0.34
0.1	−1	97.53	1.03	96.00	2.73	98.54	0.68

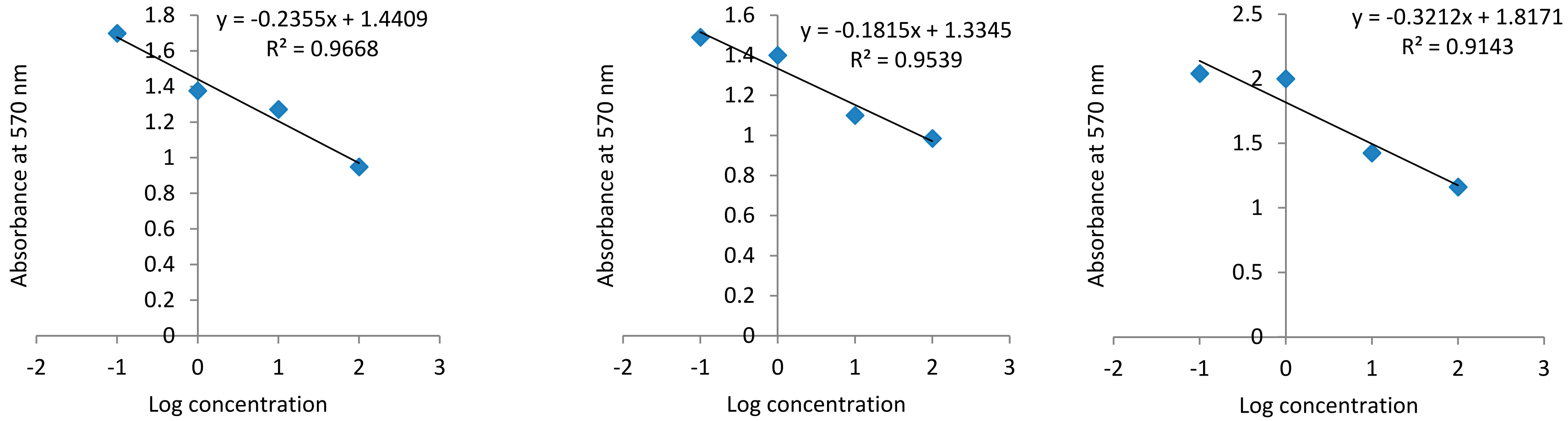

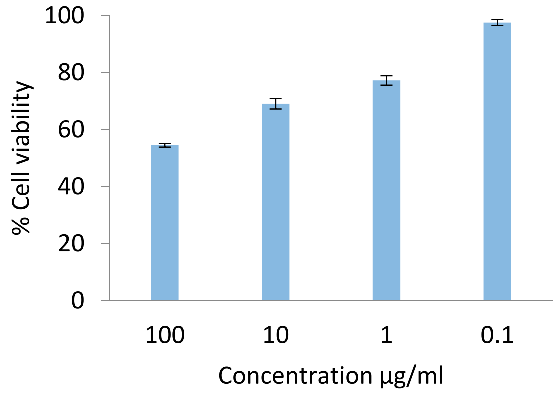

MCF-7

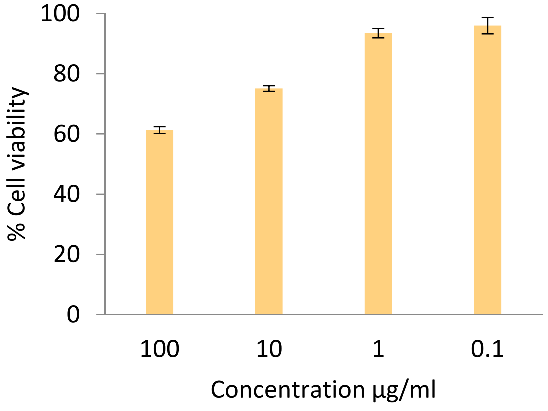

HeLa

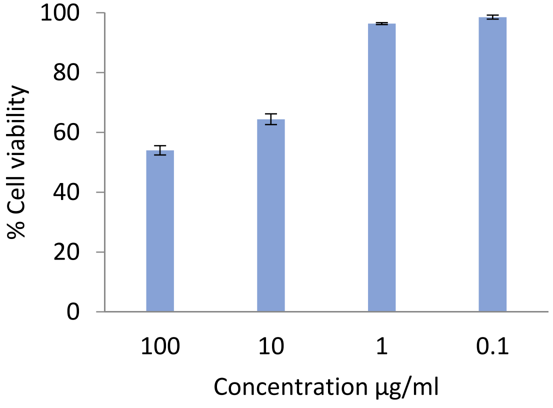

A549

Figure S52. Linear regression plots and percentage cell viability graphs of MCF-7, HeLa and A549 cells exposed to different concentrations of **3a.**

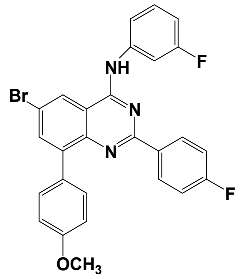

3b

Figure S53. Chemical structure of **3b.**

Table S7. Percentage cell viability of MCF-7, HeLa and A549 cells exposed to different concentrations of **3b.**

Conentration (µg/mL)	Log Conentration	%Viability MCF-7	SD	%Viability HeLa	SD	%Viability A549	SD
100	2	47.75	2.31	43.39	0.64	38.09	1.57
10	1	54.28	2.86	49.94	0.69	54.12	1.45
1	0	60.46	2.28	62.38	1.51	57.10	0.20
0.1	−1	71.35	2.56	70.18	1.72	67.58	1.37

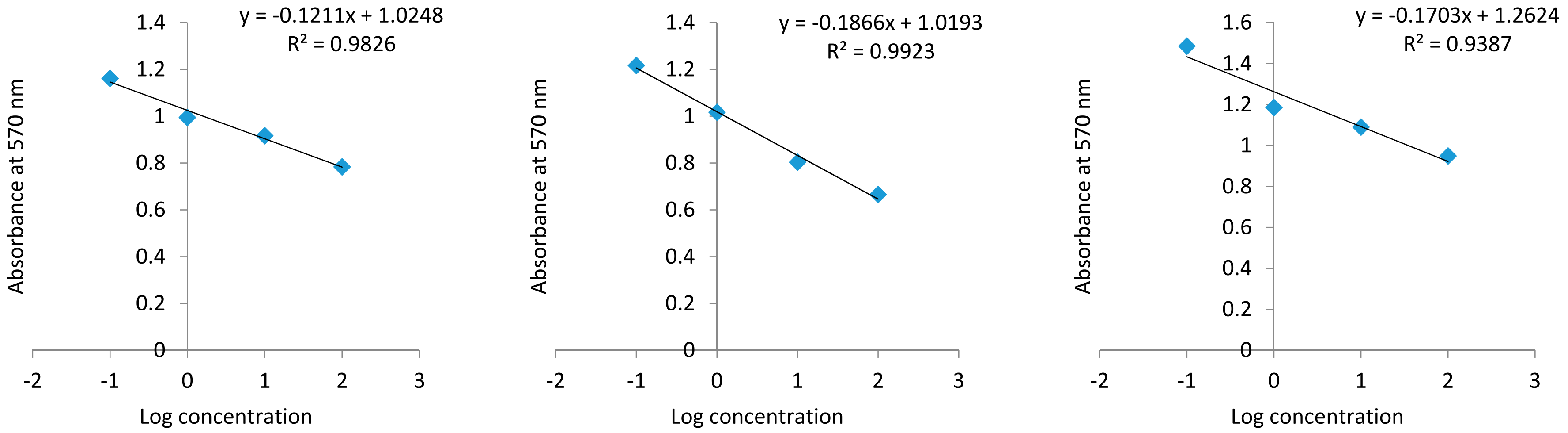

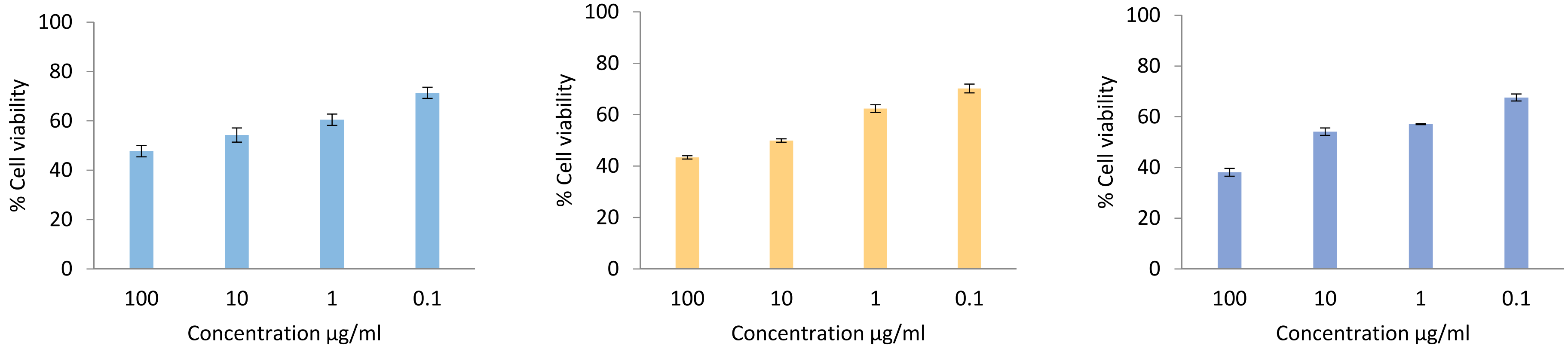

MCF-7 **HeLa** **A549**

Figure S54. Linear regression plots and percentage cell viability graphs of MCF-7, HeLa and A549 cells exposed to different concentrations of **3b.**

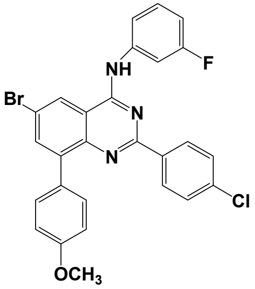

3c

Figure S55. Chemical structure of **3c.**

Table S8. Percentage cell viability of MCF-7, HeLa and A549 cells exposed to different concentrations of **3c**

Concentration (µg/mL)	Log Concentration	%Viability MCF-7	SD	%Viability HeLa	SD	%Viability A549	SD
100	2	42.10	2.60	34.69	0.73	4271	1.45
10	1	45.80	1.95	38.27	0.23	45.559	0.85
1	0	55.33	1.81	39.49	0.68	62.71	1.29
0.1	−1	59.51	2.90	45.36	1.88	66.09	1.30

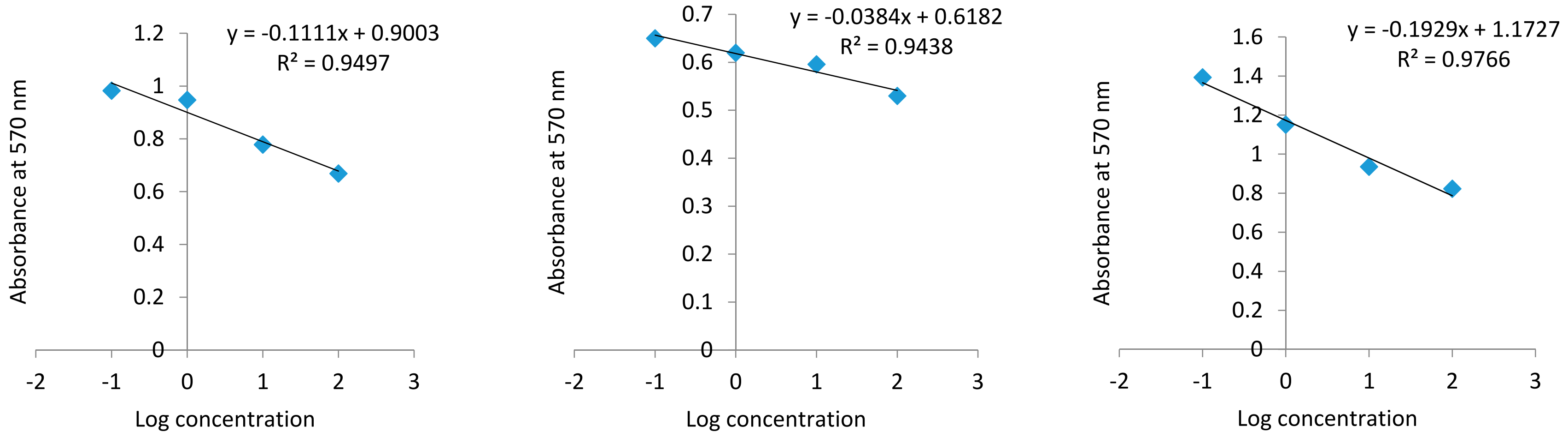

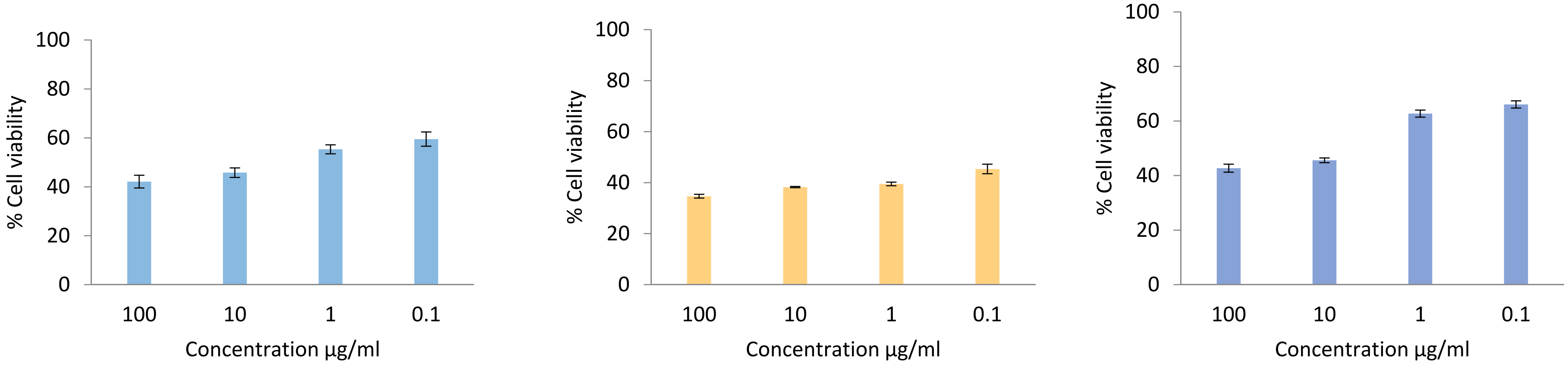

MCF-7 **HeLa** **A549**

Figure S56. Linear regression plots and percentage cell viability graphs of MCF-7, HeLa and A549 cells exposed to different concentrations of **3c.**

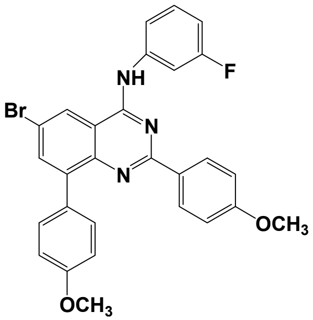

3d

Figure S57. Chemical structure of **3d.**

Table S9. Percentage cell viability of MCF-7, HeLa and A549 cells exposed to different concentrations of **3d**.

Concentration (μg/mL)	Log Concentration	%Viability MCF-7	SD	%Viability HeLa	SD	%Viability A549	SD
100	2	45.88	2.044	45.94	0.96	67.68	0.68
10	1	53.53	2.34	53.19	1.03	72.44	1.62
1	0	55.33	1.84	55.16	0.32	73.98	1.45
0.1	-1	78.72	1.95	61.41	1.70	79.06	1.68

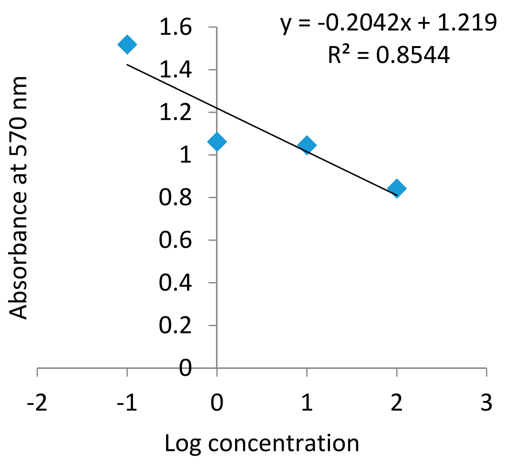

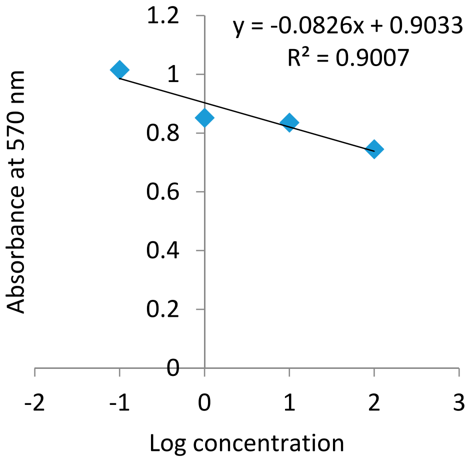

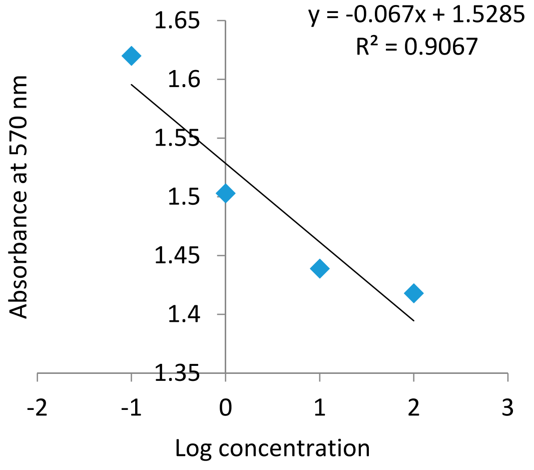

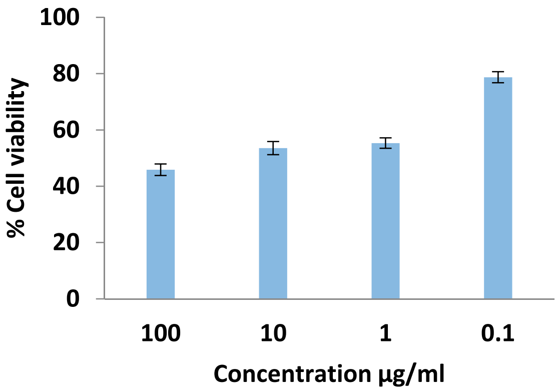

MCF-7

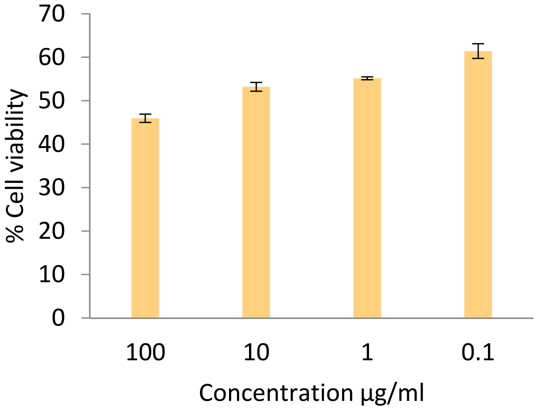

HeLa

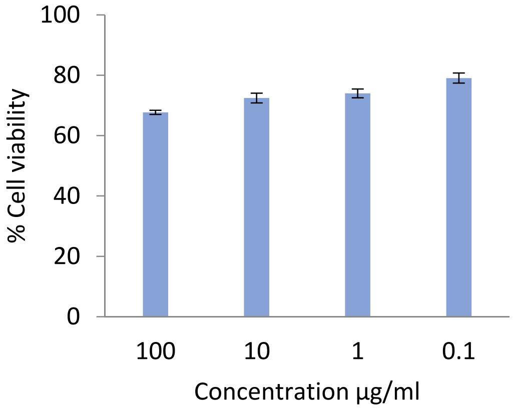

A549

Figure S58. Linear regression plots and percentage cell viability graphs of MCF-7, HeLa and A549 cells exposed to different concentrations of **3d.**

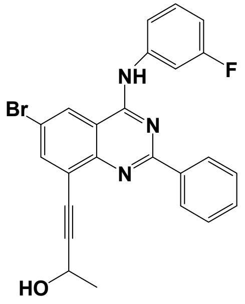

4a

Figure S59. Chemical structure of **4a.**

Table S10. Percentage cell viability of MCF-7, HeLa and A549 cells exposed to different concentrations of **4a.**

Concentration (µg/mL)	Log Concentration	%Viability MCF-7	SD	%Viability HeLa	SD	%Viability A549	SD
100	2	9.85	1.63	14.64	0.10	11.15	156
10	1	67.39	1.53	56.42	1.19	55.49	0.71
1	0	78.34	1.78	57.93	1.14	76.19	1.70
0.1	-1	95.36	2.16	68.28	1.47	78.39	1.59

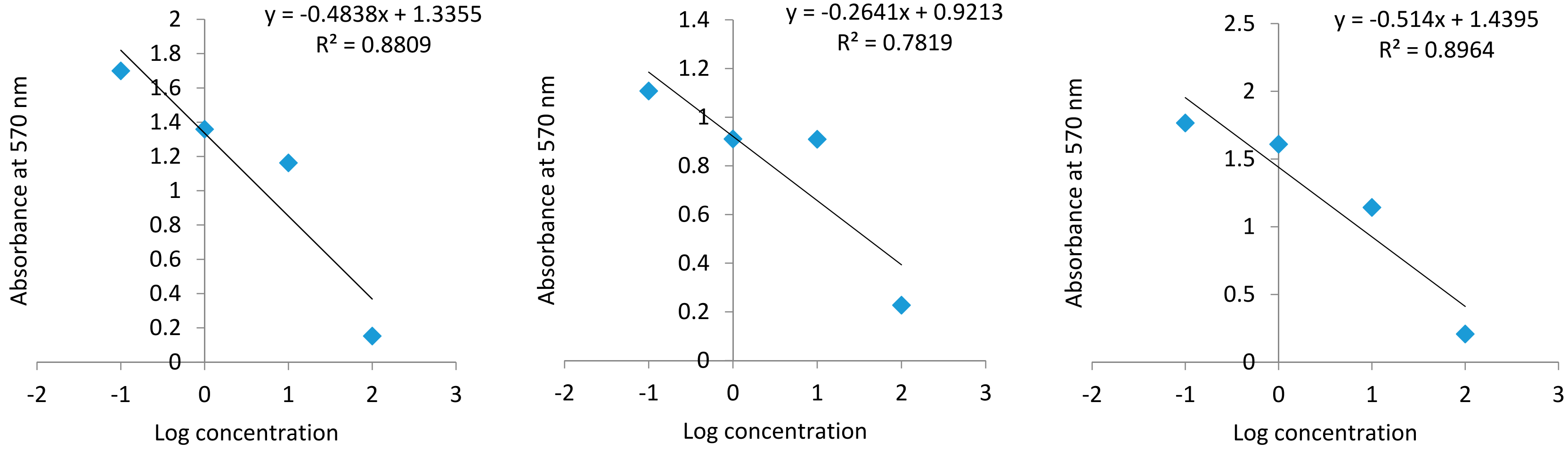

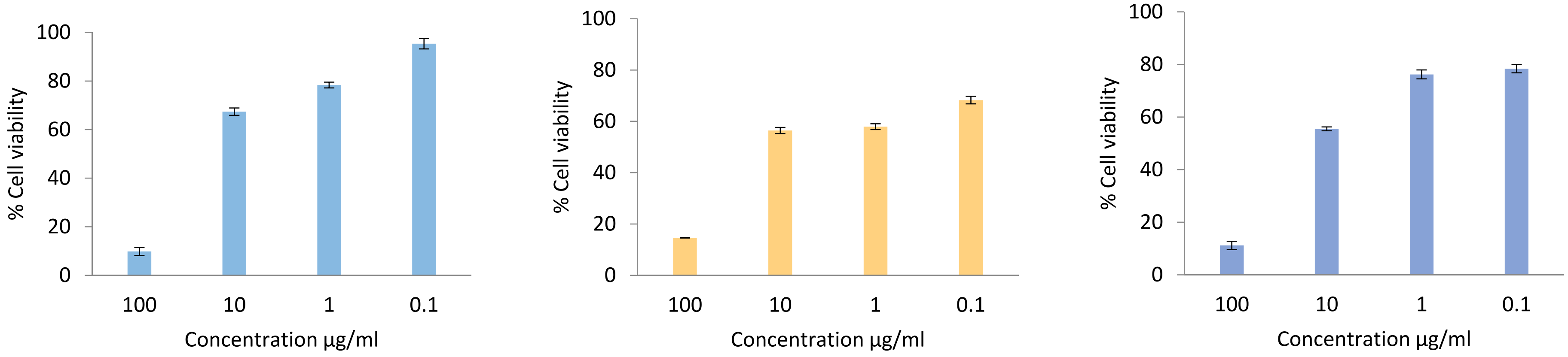

MCF-7 **HeLa** **A549**

Figure S60. Linear regression plots and percentage cell viability graphs of MCF-7, HeLa and A549 cells exposed to different concentrations of **4a.**

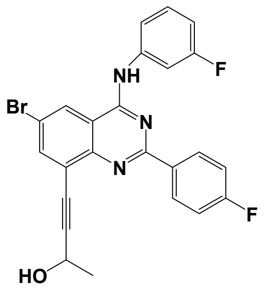

4b

Figure S61. Chemical structure of **4b.**

Table S11. Percentage cell viability of MCF-7, HeLa and A549 cells exposed to different concentrations of **4b**.

Concentration (μg/mL)	Log Concentration	%Viability MCF-7	SD	%Viability HeLa	SD	%Viability A549	SD
100	2	3.73	0.73	3.48	0.09	3.46	1.63
10	1	59.59	2.53	61.61	0.59	51.70	0.92
1	0	78.35	1.23	83.17	0.91	91.88	0.24
0.1	-1	58.45	1.08	95.10	1.84	96.53	2.78

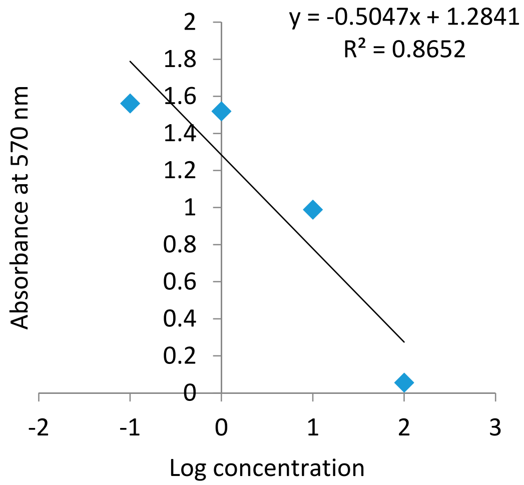

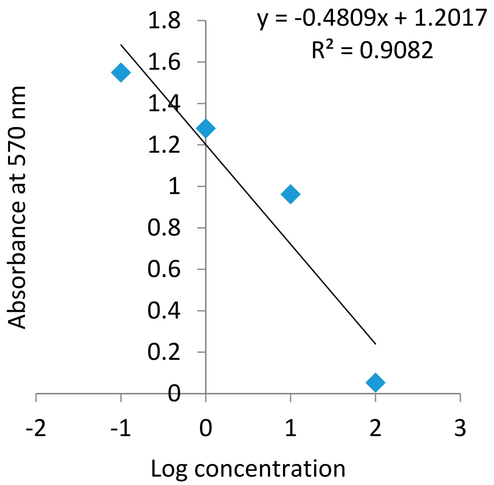

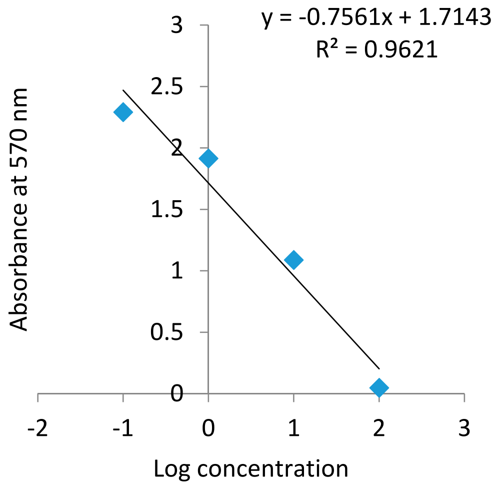

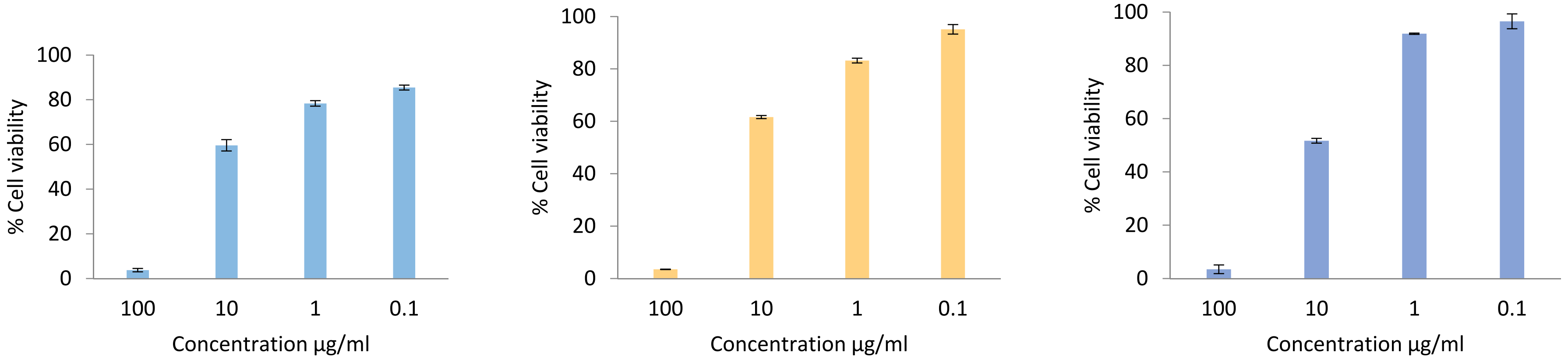

MCF-7 **HeLa** **A549**

Figure S62. Linear regression plots and percentage cell viability graphs of MCF-7, HeLa and A549 cells exposed to different concentrations of **4b.**

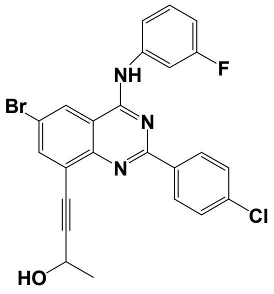

4c

Figure S63. Chemical structure of **4c.**

Table S12. Percentage cell viability of MCF-7, HeLa and A549 cells exposed to different concentrations of **4c.**

Concentration (μg/mL)	Log Concentration	%Viability MCF-7	SD	%Viability HeLa	SD	%Viability A549	SD
100	2	12.51	0.93	10.06	1.64	14.66	0.34
10	1	53.27	2.70	36.75	1.10	37.49	1.56
1	0	69.54	1.02	54.81	1.10	76.72	1.06
0.1	-1	81.64	1.26	67.05	1.20	88.69	1.19

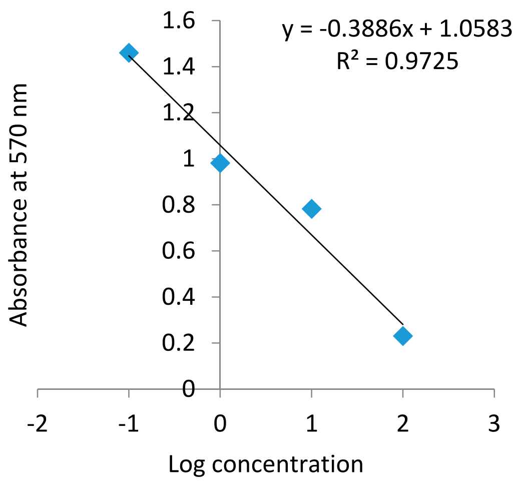

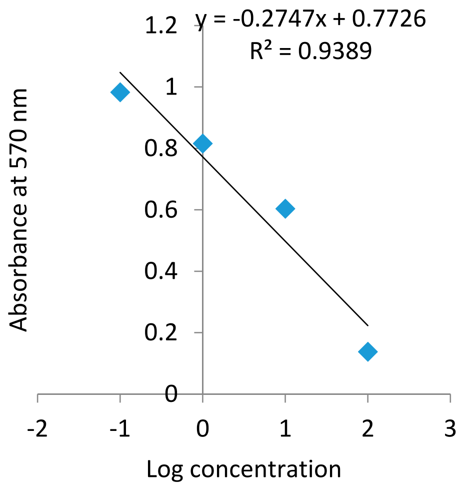

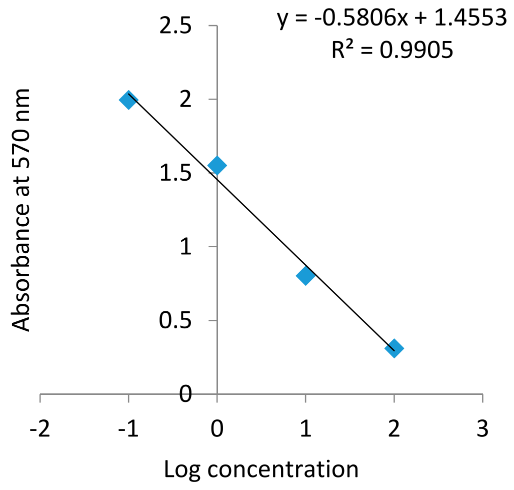

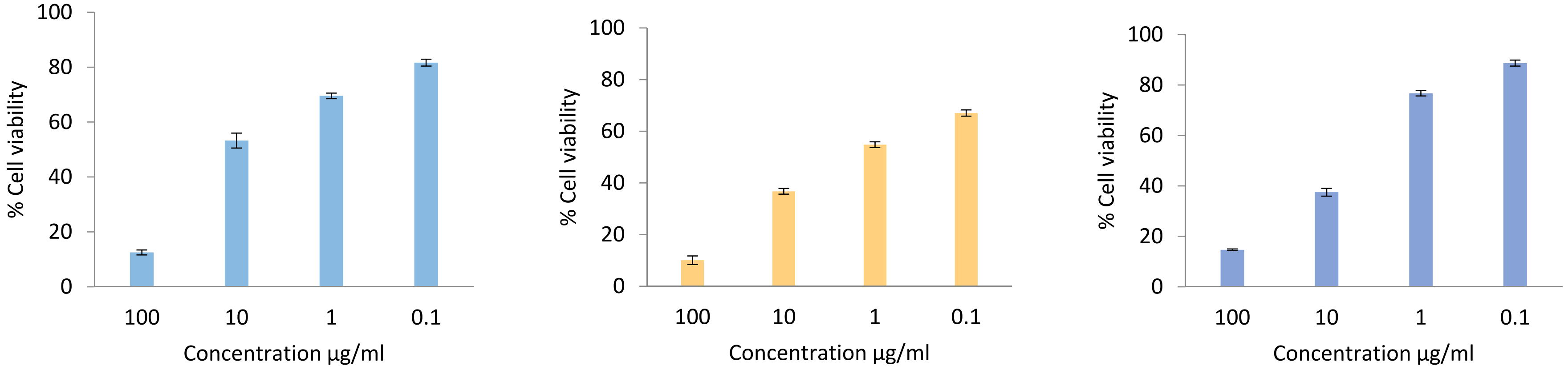

MCF-7 **HeLa** **A549**

Figure S64. Linear regression plots and percentage cell viability graphs of MCF-7, HeLa and A549 cells exposed to different concentrations of **4c.**

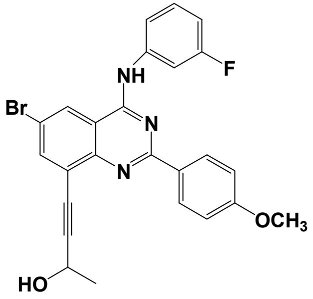

4d

Figure S65. Chemical structure of **4d.**

Table S13. Percentage cell viability of MCF-7, HeLa and A549 cells exposed to different concentrations of **4d.**

Concentration (µg/mL)	Log Concentration	%Viability MCF-7	SD	%Viability HeLa	SD	%Viability A549	SD
100	2	23.53	1.67	28.92	1.78	51.06	1.04
10	1	59.61	1.96	50.26	0.51	54.37	0.34
1	0	61.00	1.69	60.67	0.97	64.12	0.48
0.1	-1	73.99	1.71	62.89	1.51	78.38	1.62

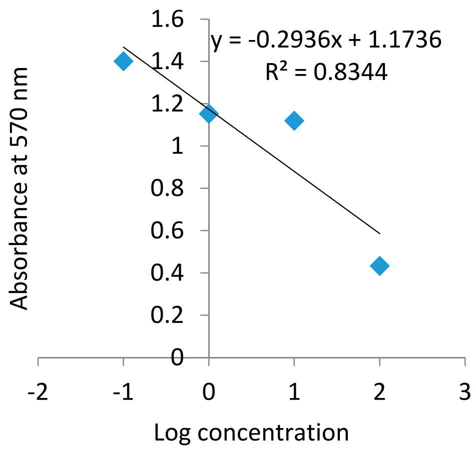

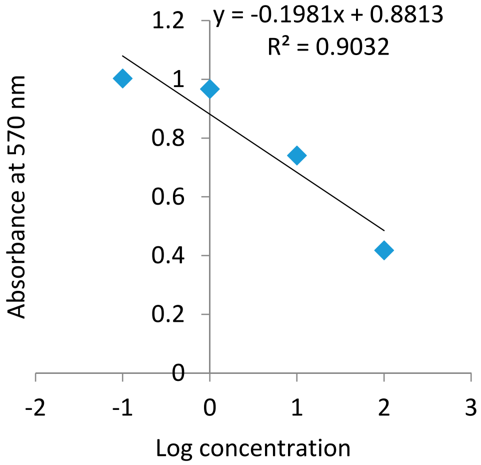

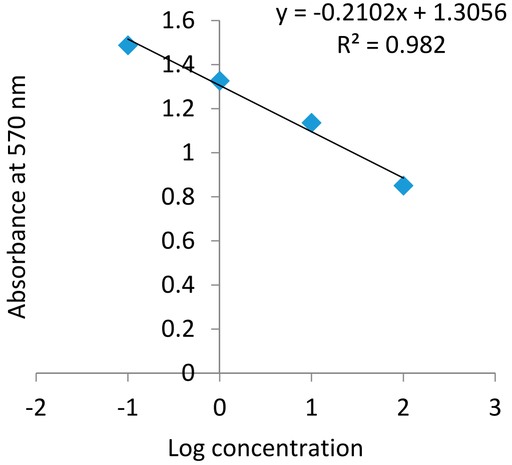

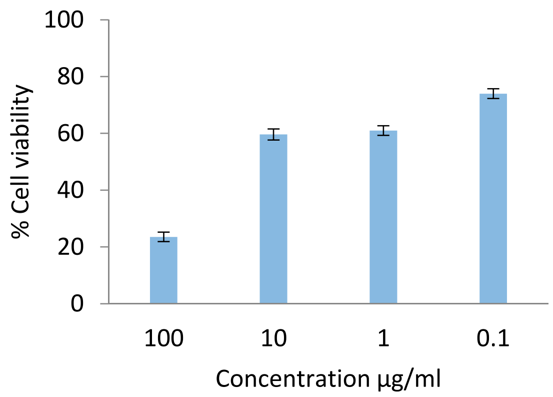

MCF-7

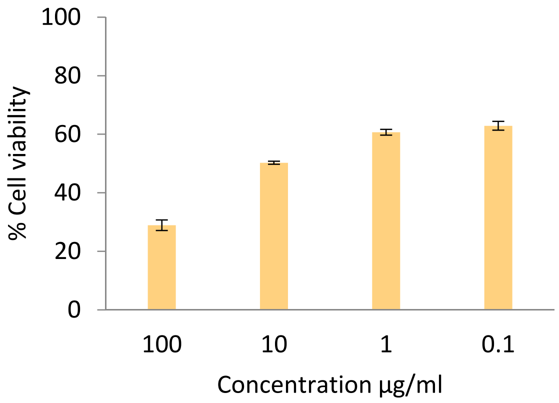

HeLa

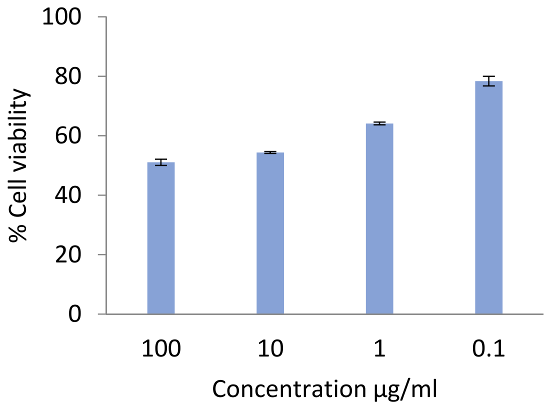

A549

Figure S66. Linear regression plots and percentage cell viability graphs of MCF-7, HeLa and A549 cells exposed to different concentrations of **4d.**

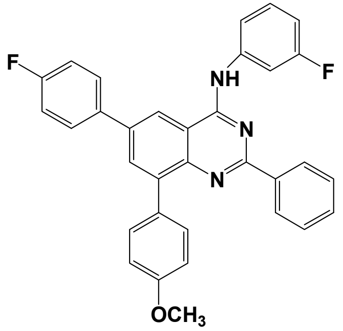

5a

Figure S67. Chemical structure of **5a**.

Table S14. Percentage cell viability of MCF-7, HeLa and A549 cells exposed to different concentrations of **5a.**

Concentration (μg/mL)	Log Concentration	%Viability MCF-7	SD	%Viability HeLa	SD	%Viability A549	SD
100	2	53.43	2.30	37.33	1.37	60.93	1.09
10	1	64.67	1.65	43.04	1.87	61.58	1.33
1	0	73.05	0.10	48.52	0.32; 2.32	80.18	1.94
0.1	-1	74.42	1.51	56.25	1.42	83.59	1.76

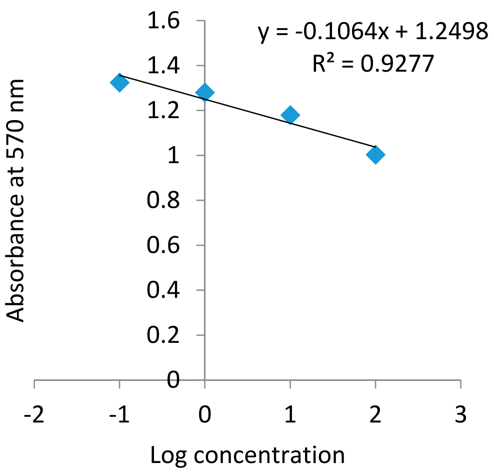

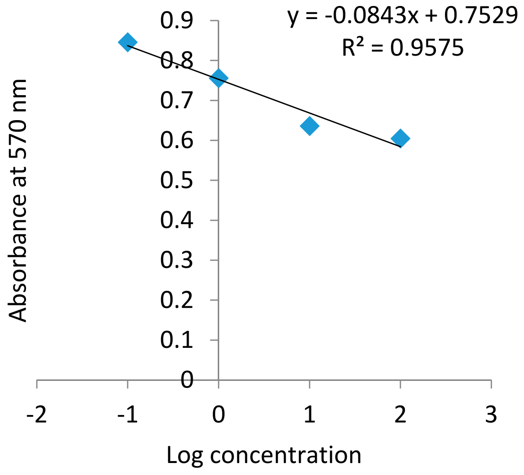

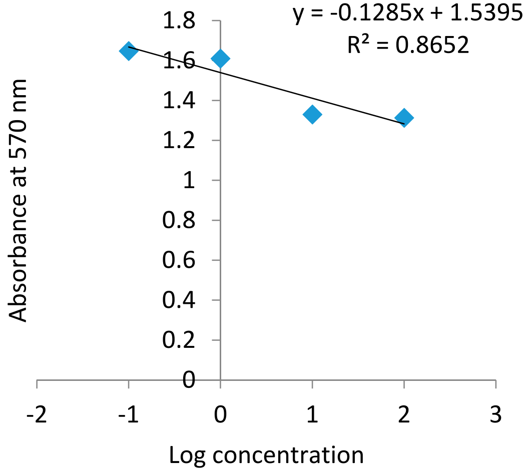

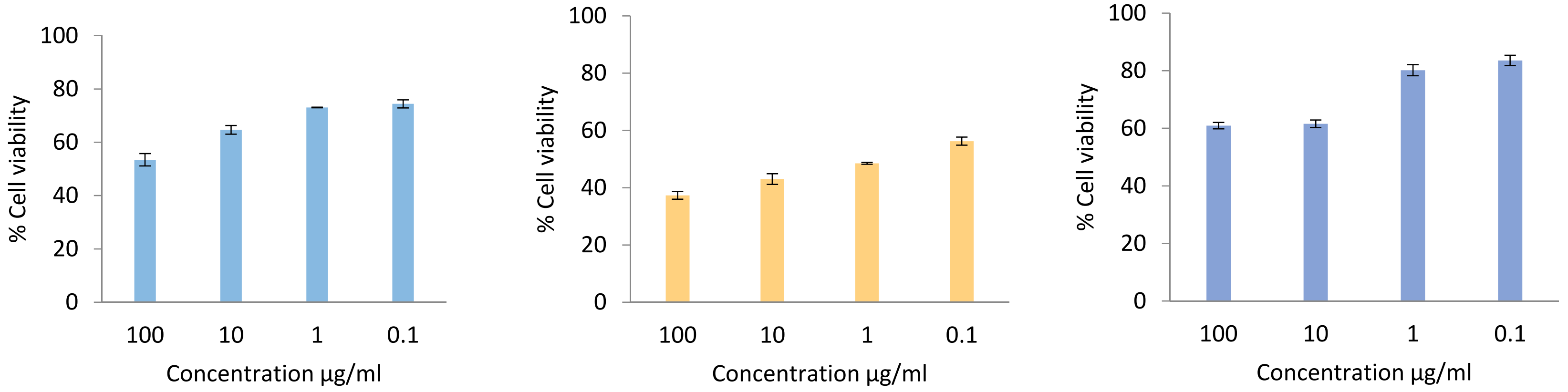

MCF-7 **HeLa** **A549**

Figure S68. Linear regression plots and percentage cell viability graphs of MCF-7, HeLa and A549 cells exposed to different concentrations of **5a**.

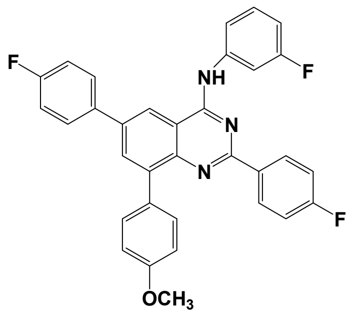

5b

Figure S69. Chemical structure of **5b**.

Table S15. Percentage cell viability of MCF-7, HeLa and A549 cells exposed to different concentrations of **5b**.

Concentration (µg/mL)	Log Concentration	%Viability MCF-7	SD	%Viability HeLa	SD	%Viability A549	SD
100	2	53.88	2.96	38.39	0.97	47.06	1.47
10	1	56.14	1.32	54.87	0.27	55.61	1.65
1	0	58.04	1.26	56.58	1.87	60.64	1.77
0.1	-1	63.90	2.53	59.93	1.60	83.60	1.16

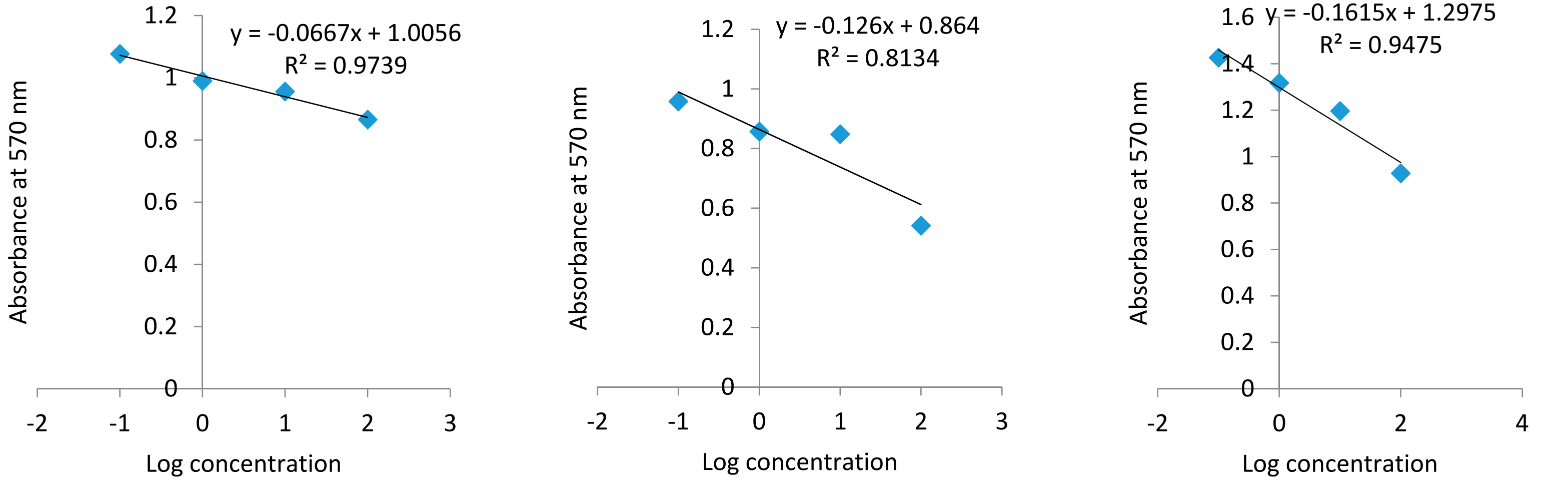

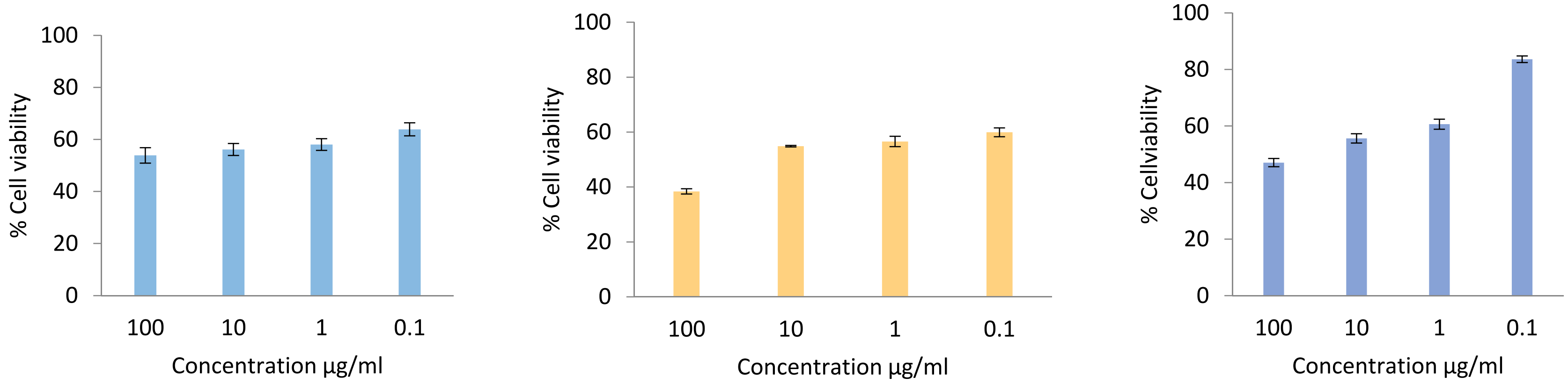

MCF-7 **HeLa** **A549**

Figure S70. Linear regression plots and percentage cell viability graphs of MCF-7, HeLa and A549 cells exposed to different concentrations of **5b.**

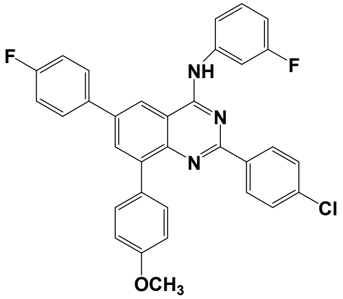

Figure S71. Chemical structure of **5c.**

Table S16. Percentage cell viability of MCF-7, HeLa and A549 cells exposed to different concentrations of **5c**.

Concentration (µg/mL)	Log Concentration	%Viability MCF-7	SD	%Viability HeLa	SD	%Viability A549	SD
100	2	47.75	1.21	42.30	1.96	52.03	0.74
10	1	54.70	1.33	64.73	1.77	60.01	1.13
1	0	71.45	0.91	74.79	0.27	76.04	1.85
0.1	-1	79.52	1.74	86.11	1.50	89.86	1.87

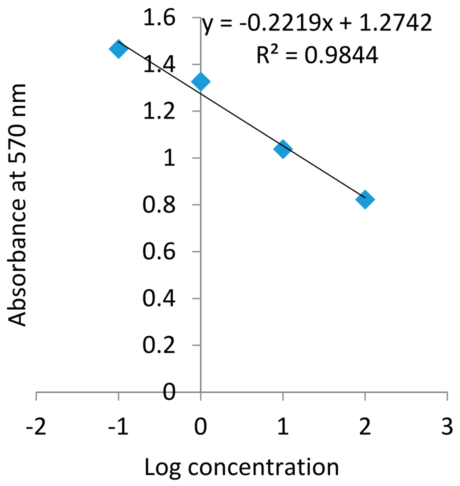

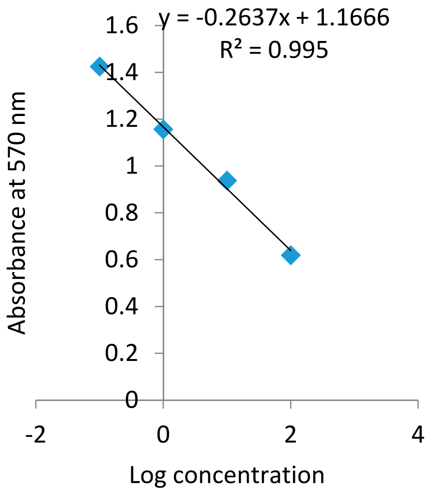

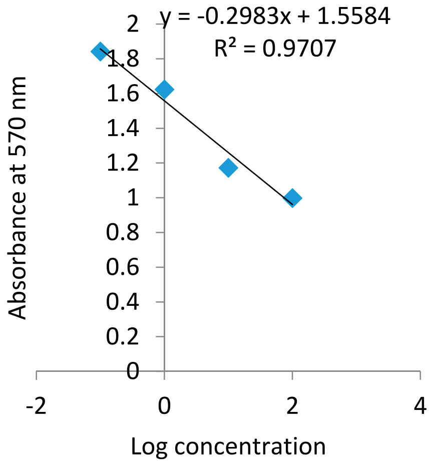

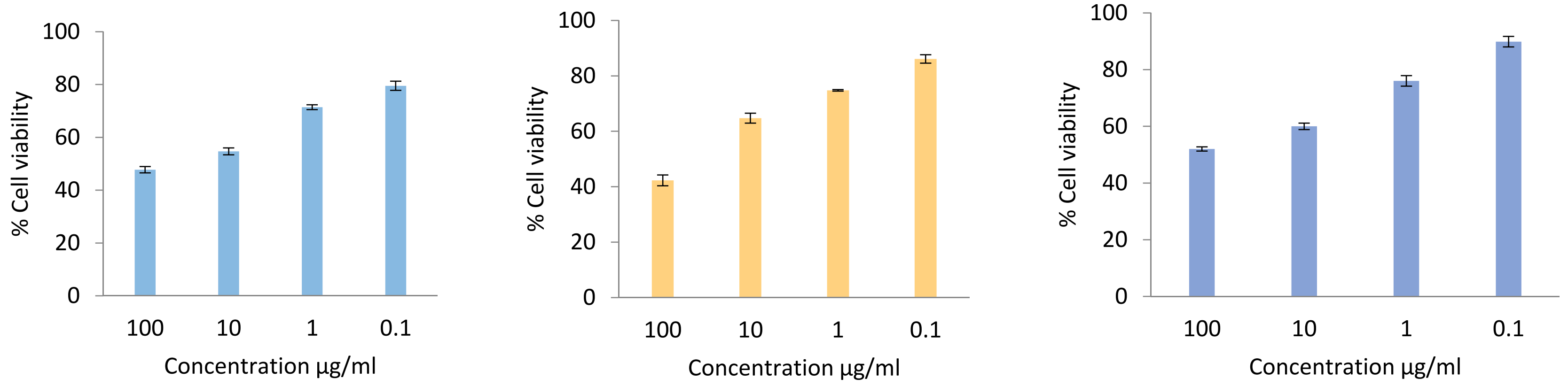

MCF-7 **HeLa** **A549**

Figure S72. Linear regression plots and percentage cell viability graphs of MCF-7, HeLa and A549 cells exposed to different concentrations of **5c.**

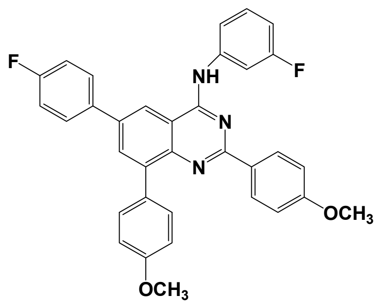

Figure S73. Chemical structure of **5d.**

Table S17. Percentage cell viability of MCF-7, HeLa and A549 cells exposed to different concentrations of **5d.**

Concentration (µg/mL)	Log Concentration	%Viability MCF-7	SD	%Viability HeLa	SD	%Viability A549	SD
100	2	66.23	0.69	66.12	0.50	64.12	1.90
10	1	67.59	0.97	73.50	1.01	71.16	1.53
1	0	85.36	1.05	81.66	0.87	86.33	1.26
0.1	-1	96.09	1.97	98.97	1.37	97.75	1.74

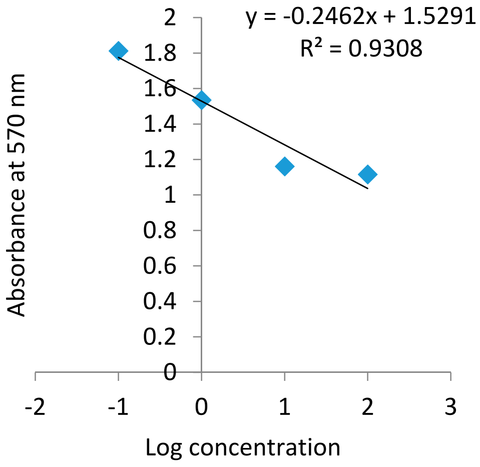

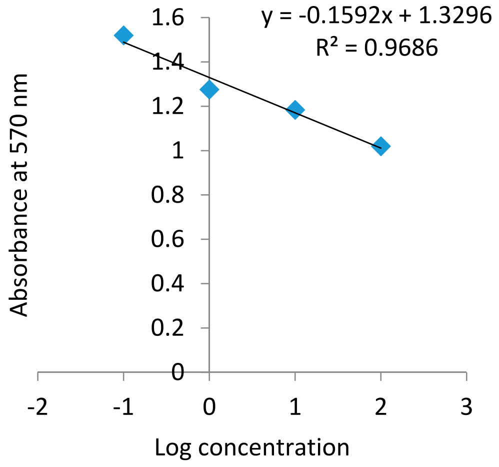

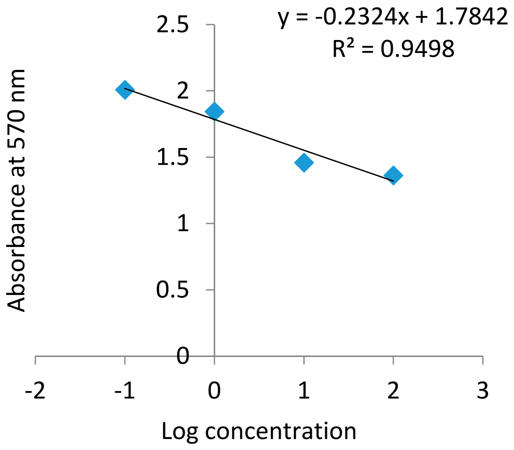

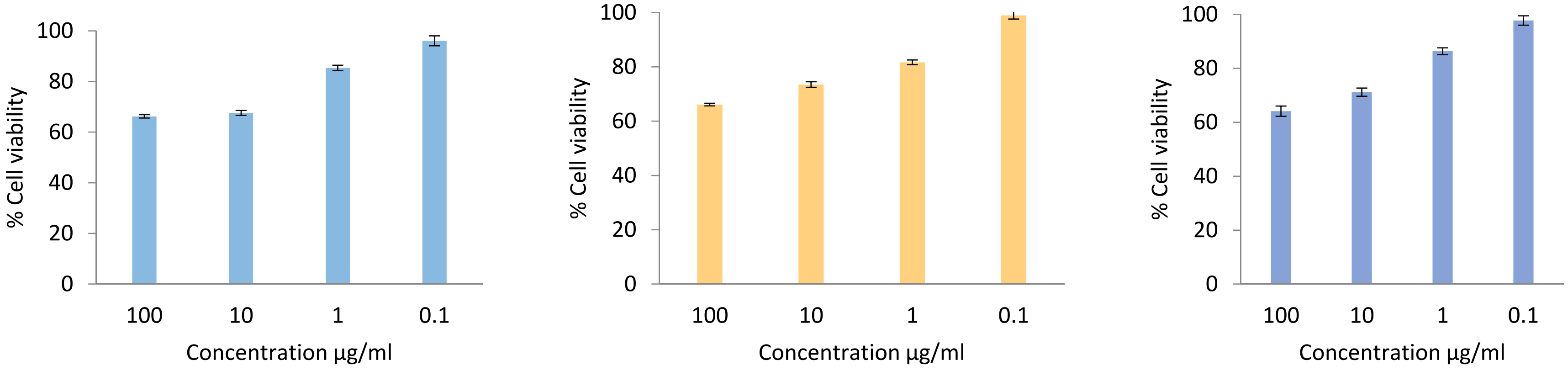

MCF-7 **HeLa** **A549**

Figure S74. Linear regression plots and percentage cell viability graphs of MCF-7, HeLa and A549 cells exposed to different concentrations of **5d.**

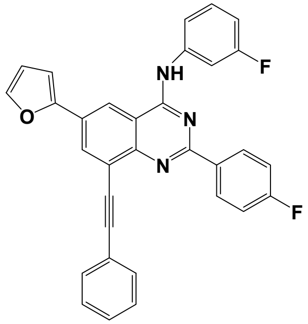

Figure S75. Chemical structure of **6b**.

Table S21. Percentage cell viability of MCF-7, HeLa and A549 cells exposed to different concentrations of **6b.**

Concentration (μg/mL)	Log Concentration	%Viability MCF-7	SD	%Viability HeLa	SD	%Viability A549	SD
100	2	24.03	0.99	27.18	1.32	30.96	0.88
10	1	76.03	1.36	50.71	1.06	87.77	1.49
1	0	90.53	2.84	63.48	1.60	96.06	1.48
0.1	-1	98.06	1.61	89.83	1.16	98.18	0.17

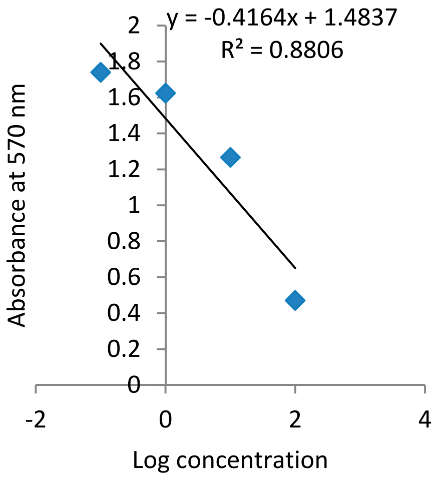

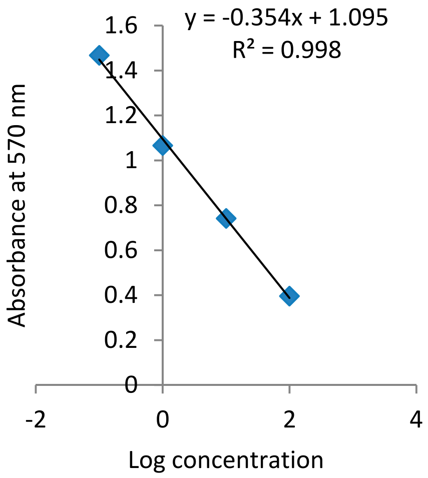

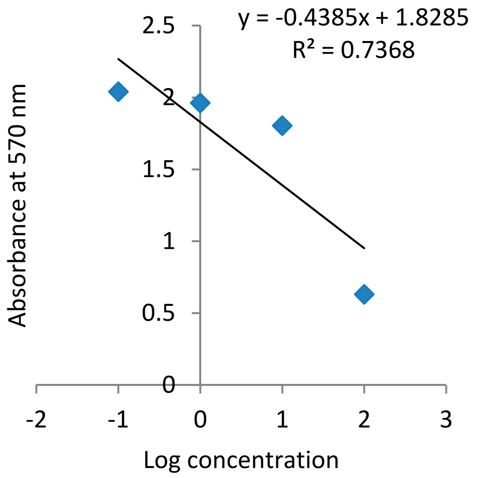

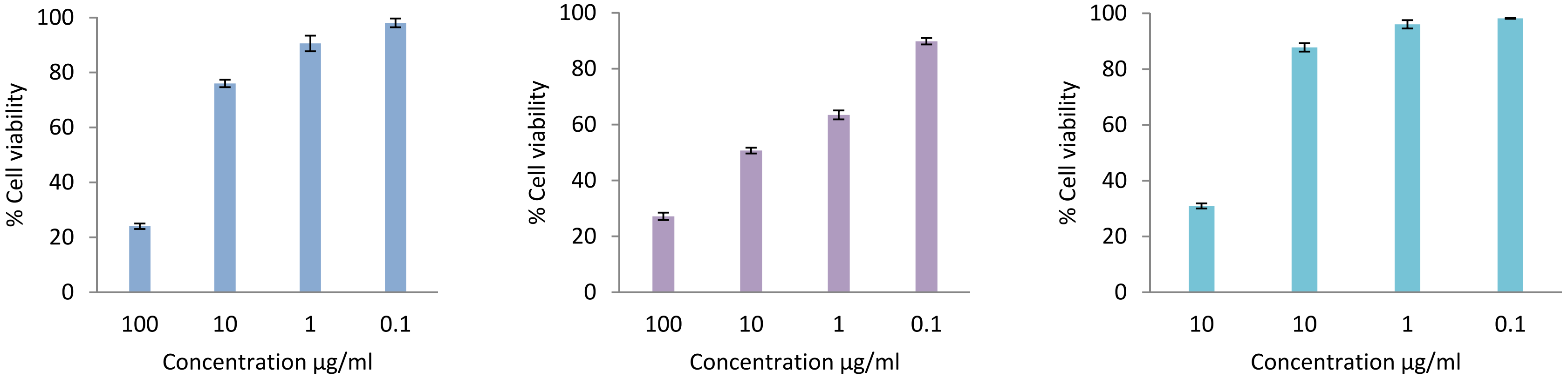

MCF-7 **HeLa** **A549**

Figure S76. Linear regression plots and percentage cell viability graphs of MCF-7, HeLa and A549 cells exposed to different concentrations of **6b.**

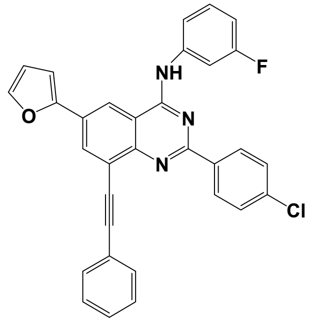

Figure S77. Chemical structure of **6c.**

Table S23. Percentage cell viability of MCF-7, HeLa and A549 cells exposed to different concentrations of **6c.**

Concentration (µg/mL)	Log Concentration	%Viability MCF-7	SD	%Viability HeLa	SD	%Viability A549	SD
100	2	53.26	2.31	53.16	1.33	66.96	0.41
10	1	58.52	2.67	53.64	0.74	78.88	1.80
1	0	66.26	3.87	58.80	0.91	86.16	1.05
0.1	-1	84.78	1.10	73.42	1.49	92.96	1.34

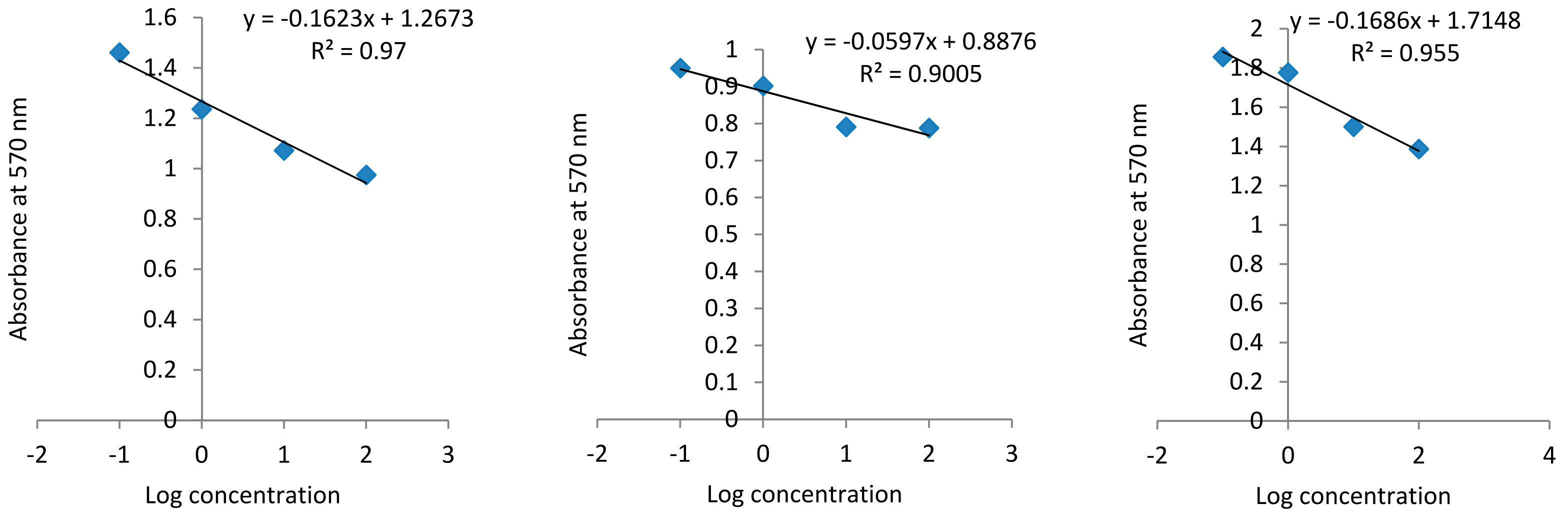

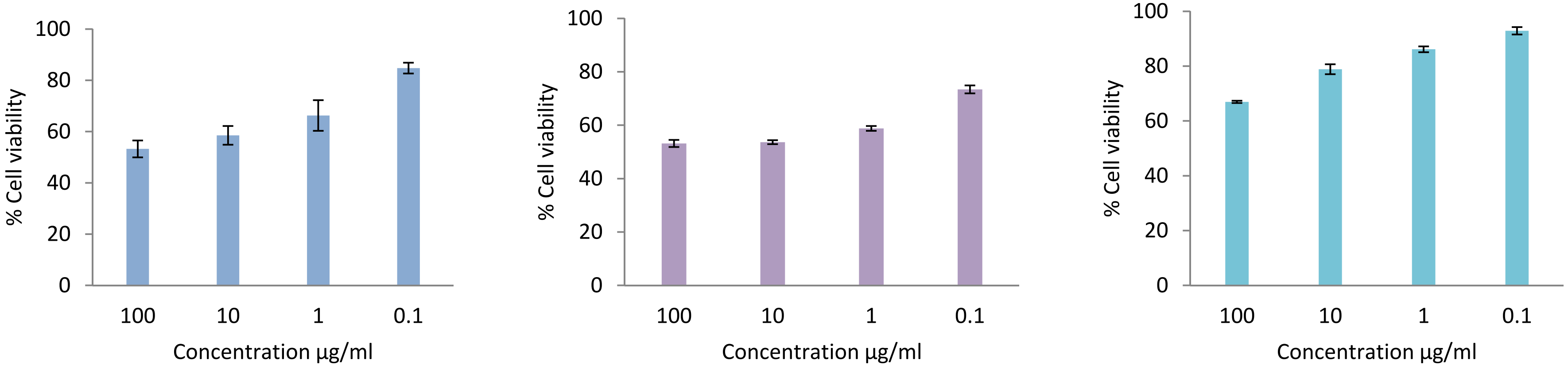

MCF-7 **HeLa** **A549**

Figure S78. Linear regression plots and percentage cell viability graphs of MCF-7, HeLa and A549 cells exposed to different concentrations of **6c.**

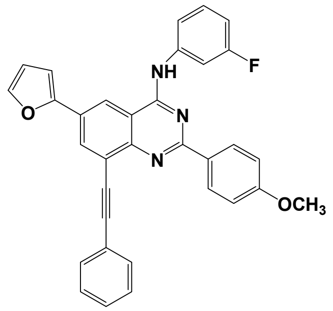

Figure S79. Chemical structure of **6d.**

Table S22. Percentage cell viability of MCF-7, HeLa and A549 cells exposed to different concentrations of **6d**.

Concentration (µg/mL)	Log Concentration	%Viability MCF-7	SD	%Viability HeLa	SD	%Viability A549	SD
100	2	72.18	1.29	77.66	1.62	67.10	1.96
10	1	76.62	1.09	87.33	0.23	93.07	1.34
1	0	83.84	2.26	93.49	1.56	95.51	1.60
0.1	-1	97.29	0.52	95.71	1.41	98.78	0.34

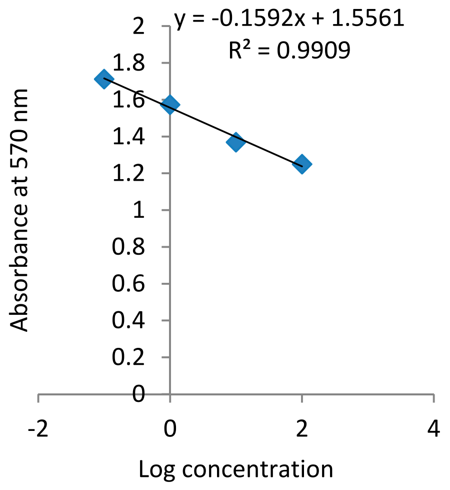

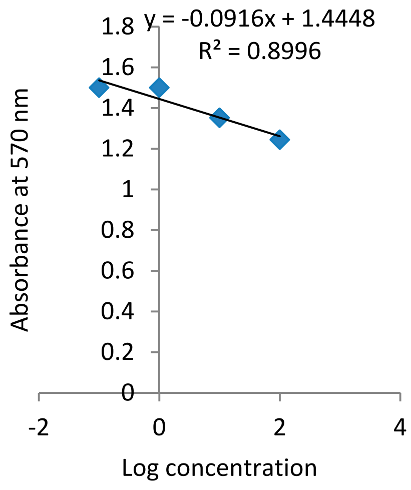

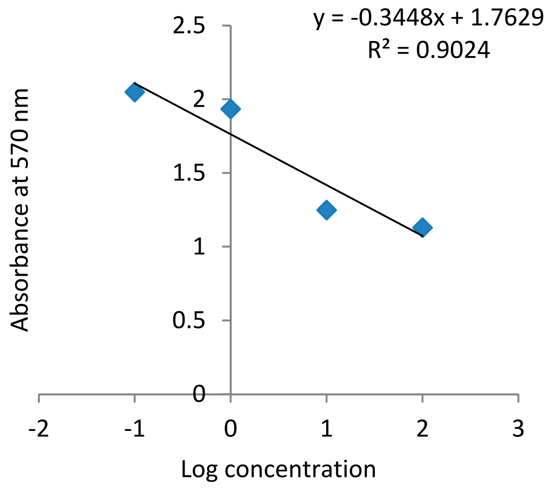

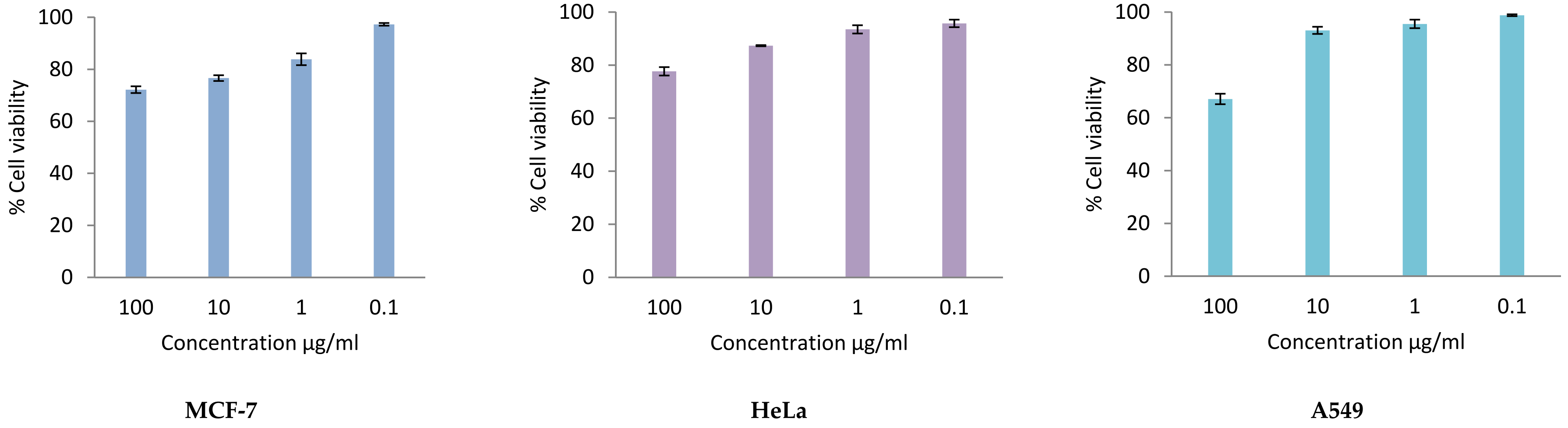

Figure S80. Linear regression plots and percentage cell viability graphs of MCF-7, HeLa and A549 cells exposed to different concentrations of **6d**.

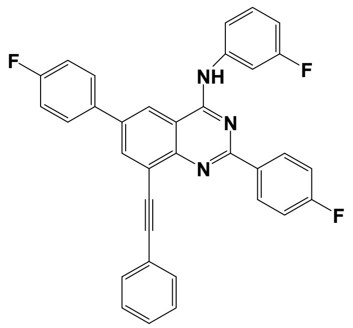

Figure S81. Chemical structure of **7a.**

Table S18. Percentage cell viability of MCF-7, HeLa and A549 cells exposed to different concentrations of **7a.**

Concentration (µg/mL)	Log Concentration	%Viability MCF-7	SD	%Viability HeLa	SD	%Viability A549	SD
100	2	41.31	0.28	38.68	1.23	57.44	0.48
10	1	46.27	0.36	44.52	1.78	62.37	1.23
1	0	52.70	1.51	62.6	1.60	68.50	1.90
0.1	-1	72.20	0.95	64.76	1.69	80.08	1.36

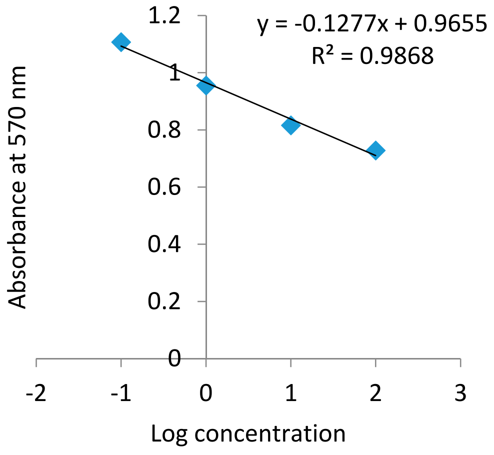

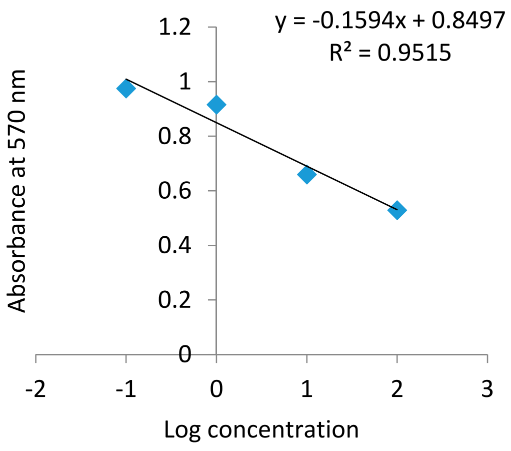

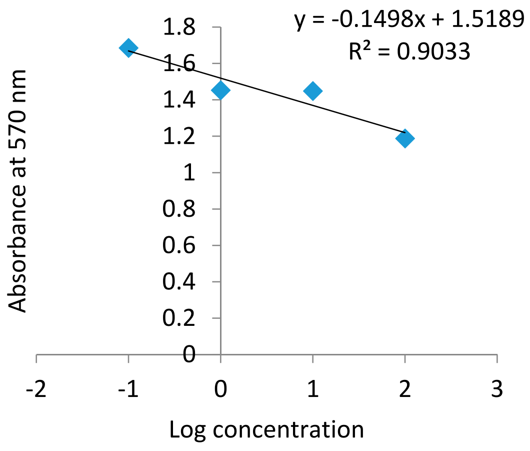

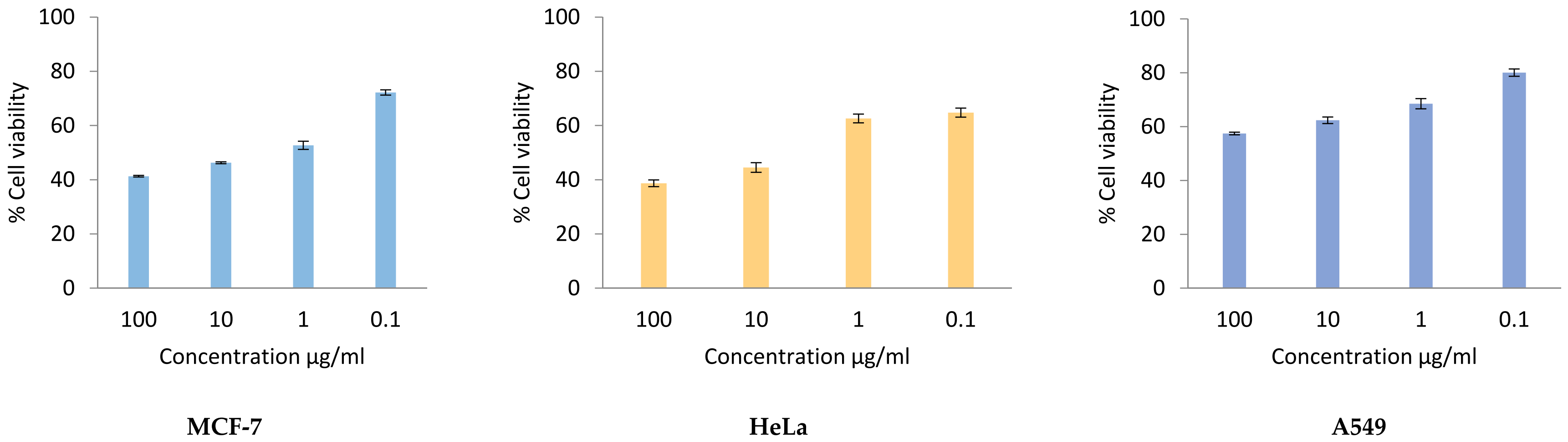

Figure S82. Linear regression plots and percentage cell viability graphs of MCF-7, HeLa and A549 cells exposed to different concentrations of **7a.**

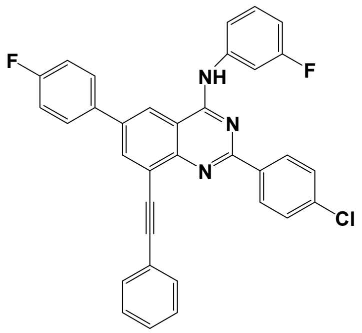

Figure S83. Chemical structure of **7b.**

Table S19. Percentage cell viability of MCF-7, HeLa and A549 cells exposed to different concentrations of **7b.**

Concentration (µg/mL)	Log Concentration	%Viability MCF-7	SD	%Viability HeLa	SD	%Viability A549	SD
100	2	64.42	1.61	53.80	1.23	66.48	1.36
10	1	65.68	0.71	55.03	2.14	70.92	1.03
1	0	67.45	1.29	74.47	1.01	76.21	1.57
0.1	-1	70.65	1.87	75.69	1.46	87.67	1.62

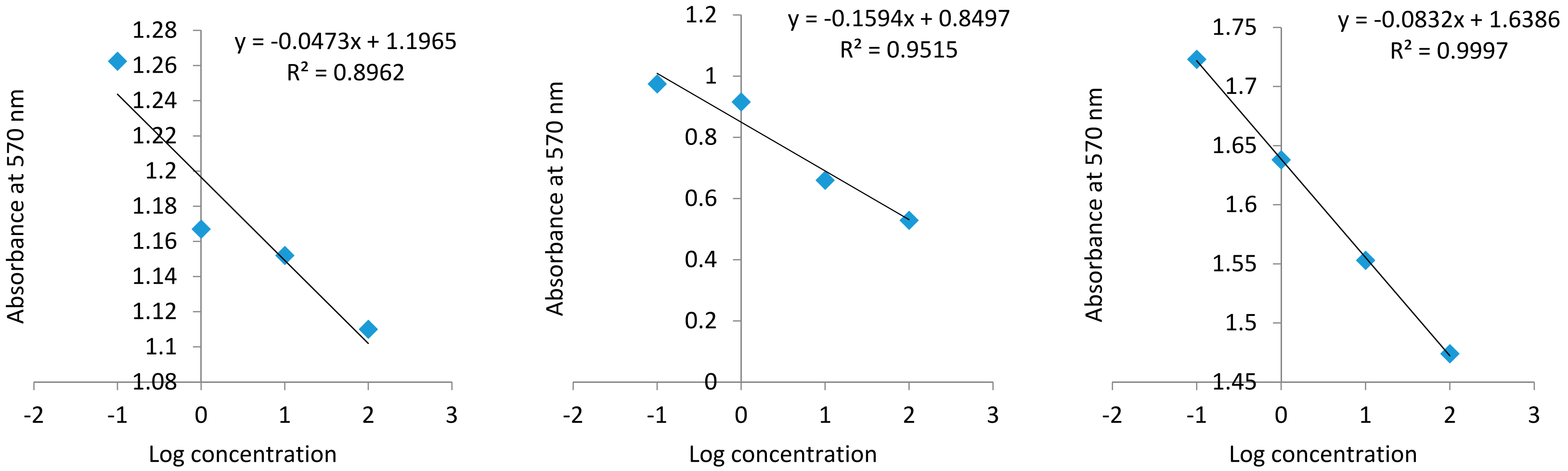

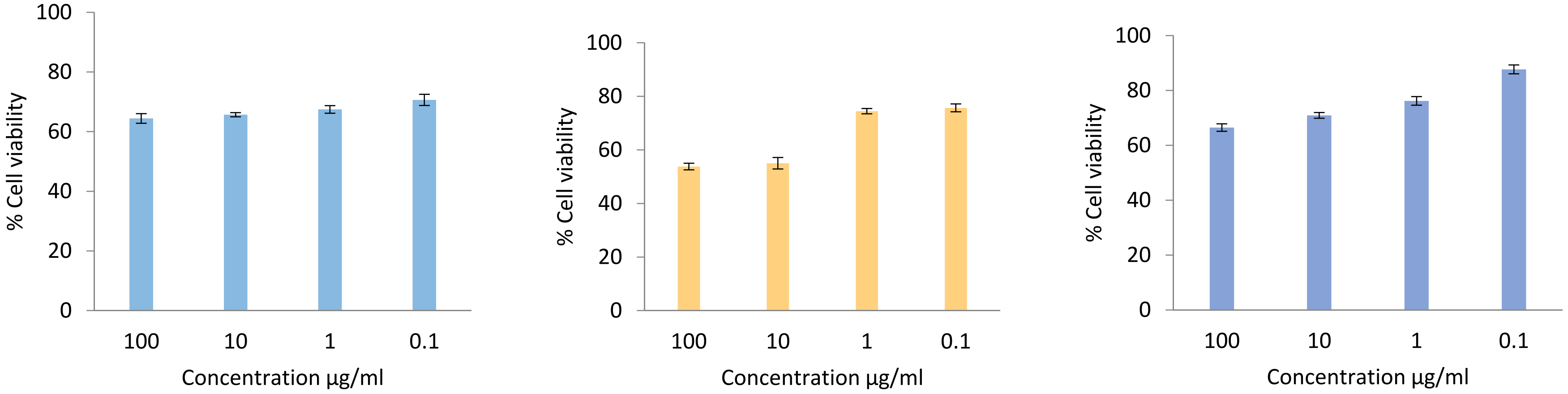

MCF-7 **HeLa** **A549**

Figure S84. Linear regression plots and percentage cell viability graphs of MCF-7, HeLa and A549 cells exposed to different concentrations of **7b.**

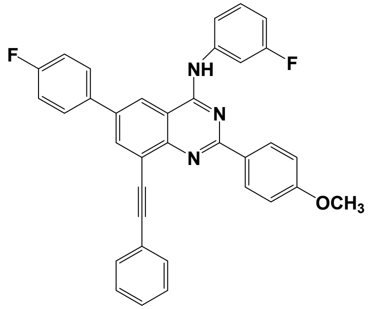

Figure S85. Chemical structure of **7c.**

Table S20. Percentage cell viability of MCF-7, HeLa and A549 cells exposed to different concentrations of **7c**

Concentration (µg/mL)	Log Concentration	%Viability MCF-7	SD	%Viability HeLa	SD	%Viability A549	SD
100	2	41.86	1.67	59.77	1.56	82.39	0.20
10	1	55.18	1.39	74.89	1.78	84.46	0.41
1	0	64.3	2.15	75.79	1.32	93.83	1.21
0.1	-1	87.88	2.01	81.01	1.78	97.82	0.34

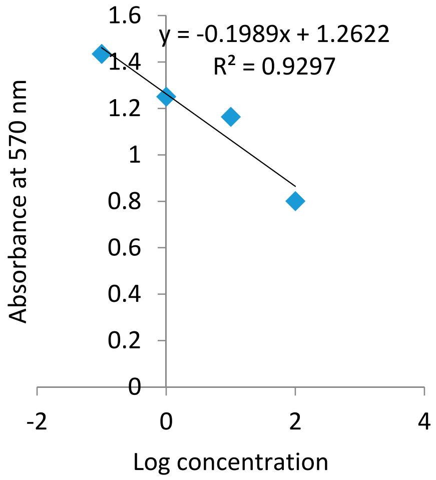

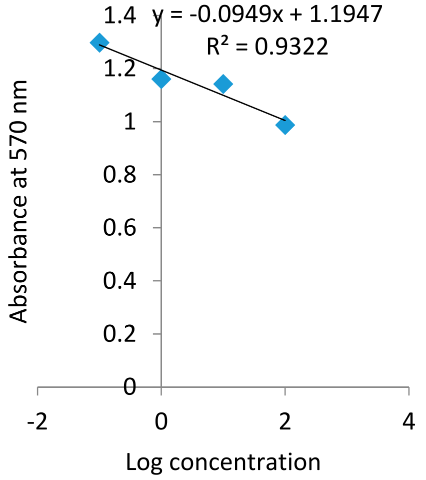

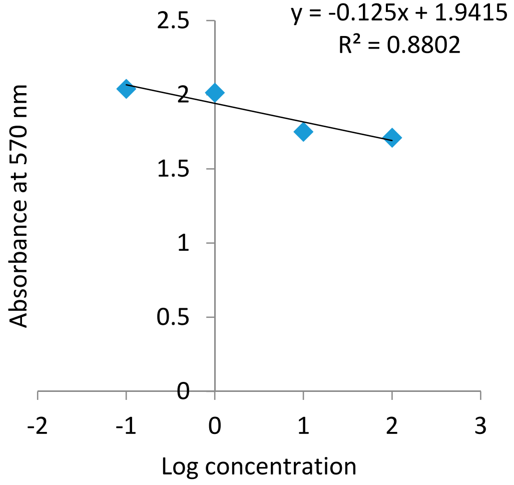

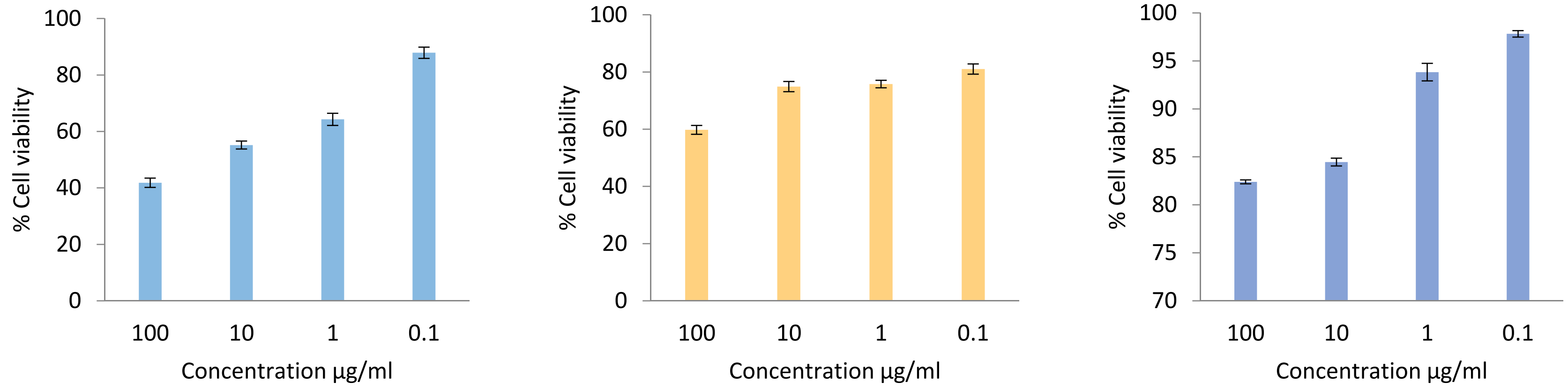

MCF-7 **HeLa** **A549**

Figure S86. Linear regression plots and percentage cell viability graphs of MCF-7, HeLa and A549 cells exposed to different concentrations of **7c**.
